# Magnitude of Cardiovascular Risk Factors in Rural and Urban Areas in Benin: Findings from a Nationwide Steps Survey

**DOI:** 10.1371/journal.pone.0126441

**Published:** 2015-05-06

**Authors:** Yessito Corine Nadège Houehanou, Philippe Lacroix, Gbedecon Carmelle Mizehoun, Pierre-Marie Preux, Benoit Marin, Dismand Stephan Houinato

**Affiliations:** 1 INSERM UMR 1094, Tropical Neuroepidemiology, Limoges, France; 2 Health ministry of Benin, National Non-communicable Diseases Control Program, Porto-Novo, Benin; 3 University hospital of Limoges (France), Department of vascular medicine, Limoges, France; 4 University of Limoges (France), School of Medicine, Institute of Neuroepidemiology and Tropical Neurology, CNRS FR 3503 GEIST, Limoges, France; 5 University hospital of Limoges (France), Functional unit of Clinical Research and Biostatistics, Limoges, France; 6 Faculty of health sciences of Cotonou (Benin), Laboratory of non-communicable and neurologic diseases epidemiology, Cotonou, Benin; University of Tennessee, UNITED STATES

## Abstract

Data on variations in the frequency of cardiovascular risk factors (CVRF) in sub-Saharan populations are limited, particularly with regard to Benin.

**Objective:**

To describe and compare the prevalences of CVRF in urban and rural populations of Benin.

**Methods:**

Subjects were drawn from participants in the Benin Steps survey, a nationwide cross-sectional study conducted in 2008 using the World Health Organisation (WHO) stepwise approach to surveillance of chronic disease risk factors. Subjects aged above 24 and below 65 years were recruited using a five-stage random sampling process within households. Sociodemographic data, behavioral data along with medical history of high blood pressure and diabetes mellitus were collected in Step 1. Anthropometric parameters and blood pressure were measured in Step 2. Blood glucose and cholesterol levels were measured in Step 3. CVRF were defined according to WHO criteria. The prevalences of CVRF were assessed and the relationships between each CVRF and the area of residence (urban or rural), were evaluated using multivariable logistic regression models.

**Results:**

Of the 6762 subjects included in the study, 2271 were from urban areas and 4491 were from rural areas. High blood pressure was more prevalent in urban than in rural areas, 29.9% (95% confidence intervals (95% CI): 27.4, 32.5) and 27.5% (95% CI: 25.6, 29.5) respectively, p = 0.001 (p-value after adjustment for age and gender). Obesity was more prevalent in urban than in rural areas, 16.4% (95% CI: 14.4, 18.4) and 5.9% (95% CI: 5.1, 6.7), p<0.001. Diabetes was more prevalent in urban than in rural areas, 3.3% (95% CI: 2.1, 4.5) and 1.8% (95% CI: 1.2, 2.4), p = 0.004. Conversely, daily tobacco smoking was more prevalent in rural than in urban areas, 9.3% (95% CI: 8.1, 10.4) and 4.3% (95% CI: 3.1, 5.6), p<0.001. No differences in raised blood cholesterol were noted between the two groups.

**Conclusion:**

According to our data, CVRF are prevalent among adults in Benin, and variations between rural and urban populations are significant. It may be useful to take account of the heterogeneity in the prevalence of CVRF when planning and implementing preventive interventions.

## Introduction

Cardiovascular diseases (CVD) are the leading cause of disability and premature death in the world. In 2010, they were associated with 15.6 million deaths [1]. About 80% of these CVD occur in the low or middle income countries. Sub-Saharan Africa is today faced with a double burden of communicable and non-communicable diseases (NCDs). The epidemiological transition is associated with parallel increases in CVD and their risk factors [2–10]. Some of them are preventable, such as tobacco smoking, obesity, high blood pressure (HBP), diabetes mellitus and raised blood LDL cholesterol. Simple and effective strategies are available for their primary prevention [11] based on identification of subjects “at risk”, and control of the main risk factors within populations. Reliable information is valuable to those planning health services, and in determining public health priorities. The World Health Organization (WHO) recommends in low-income countries standardized collection of data on the risk factors for NCDs according to a stepwise approach. Step 1 involves behavioral factors. The next two include anthropometric (Step 2) and biological (Step 3) factors, all of which are adaptable depending on resources [12].

In Benin, a lack of information on risk factors common to the NCDs in the population prompted a national survey in 2008, conducted according to The WHO Stepwise approach to surveillance of NCDs risk factors. The investigation was supported by the health ministry and WHO. To date, this is the only extended database available in Benin on risk factors common to NCDs.

Differences in the prevalences of cardiovascular risk factors (CVRF) have been reported between rural and urban areas in several tropical countries. Higher prevalences of obesity and raised blood glucose have been described in urban compared to rural areas [13–18]. Regarding HBP, differences between rural and urban settings varied between studies [19–22]. In Benin, data are limited on the variation of these risk factors between the urban and rural populations.

The objective of this study was to describe and compare the prevalences of CVRF in Benin between urban and rural populations, using the Steps survey database.

## Materials and Methods

### Benin description

Located in Sub-Saharan Africa, Benin covers an area of 114,763 sq. km. Its population was estimated at 9,983,884 inhabitants in the 2013 census [23]. Children under 15 years of age comprise 49% of the population, and women 51.2%. Life expectancy at birth is 59.2 years. Benin is a low-income country where the main economic activities are agriculture, craft industries and informal trade. The minimum monthly wage is approximately 40 €. The average monthly income of Beninese households is approximately 200 € [24]. There is no compulsory sickness insurance scheme. Infectious diseases such as malaria, acute respiratory infections and diarrhea are the main causes of morbidity [25].

### Benin Steps survey: design, population, recruitment, data collection

Benin Steps survey was conducted within the national NCDs Control Program. Ethical approval was granted by the “scientist and ethic committee for Benin Steps survey 2008” of the health ministry of Benin. Authorization was obtained from the health ministry of Benin before the study began.

This was a cross-sectional survey of adults aged above 24 and below 65 years living in Benin for at least 6 months prior to the date of the survey. Pregnant women and people who could not complete the survey were excluded. Written consent was obtained from all participants.

Recruitment was based on a random five-stage sampling frame. Sixty of 546 districts were randomly selected according to the sizes of their populations. In each district retained, a list of neighborhoods or villages was drawn up and half were selected. In each neighborhood retained, dwellings, households, and then subjects were randomly selected. An investigator went to the center of each neighborhood or village and randomly chose a direction to go before entering one out of every two dwellings. In the dwellings retained, he listed the households and randomly selected one out of two. Within each household, the participant was identified using the Kish method [26]. This procedure was followed until the predetermined sample was obtained for the neighborhood or village concerned.

The sample size was determined by selecting a 1.5% precision level, a 5% type I error level, and a theoretical HBP prevalence of 27.8% [27]. It was multiplied by two considering that a district corresponded to a cluster of individuals and selecting a cluster effect of 2. A sample size of 6853 subjects was found to be mandatory. Recruitment of 116 subjects was provided by district. The number of subjects investigated by village or neighborhood was proportional to population size.

The data were collected from 1 July to 24 August 2008. A French version of the WHO Steps survey tool was adapted and printed for the purpose. It included sociodemographic and behavioral variables, health history and questions on medication for HBP and diabetes mellitus (S1 Text). Use of medication focused on the two weeks preceding the day of the interview. Oral translations were made in the four main local languages (Fon, Yoruba, Bariba and Dendi) and were validated by professional local translators. Interviewers, who administered the questionnaire in either French of local languages of the areas, were trained in questionnaire administration. In Step 1, a structured interview was conducted by the investigator with the participant. In Step 2, weight, height, waist and blood pressure (BP) were measured. Physical body measurements were documented with devices that meet the WHO standards, according to the recommended procedures for Steps survey. BP was measured in the seated position, on the right arm, after the participant had rested for 15 min, by a licensed nurse or a medical student using an OMRON electronic BP monitor with a cuff. Three measurements were taken at 5-min intervals; the mean of the last two measurements was used to define BP.

In Step 3, biological measures were systematically proposed to six subjects out of ten. Capillary blood glucose and total cholesterol were measured using an ACCUTREND R machine. Blood glucose was measured in the morning, after at least 8 hours of fasting. A drop of blood from a finger was examined with an adapted test strip. Blood total cholesterol was measured immediately after the glucose level using the same process.

### Authorization

Authorization to use the Steps survey database for a scientific purpose was granted by the national NCDs control program of the Benin health ministry.

### Definitions

Definitions of variables were based on WHO criteria [12]. Significant alcohol drinking was defined as consumption ≥ 4 days per week. Low fruit and vegetable intake was defined as consumption of ≤ 5 servings of fruits or vegetables per day.

HBP was defined as diastolic BP ≥ 90 mmHg or systolic BP ≥ 140 mmHg or current receipt of medication for hypertension. BMI (body mass index) obesity was defined as BMI ≥ 30.0 kg/m^2^. Abdominal obesity was defined as waist circumference > 88 cm in women and > 102 cm in men. Overall obesity was defined as BMI kg/m^2^ ≥ 30.0 or presence of abdominal obesity. Raised blood glucose was defined as fasting capillary blood glucose level ≥ 6.1 mmol/L or current receipt of medication for diabetes mellitus. Raised blood cholesterol was defined as blood total cholesterol level ≥ 6.5 mmol/L.

The classification of areas of residence was based on the list of villages and neighborhoods used in the Benin Steps survey. Urban and rural areas were defined according to Benin’s Statistical and Economic Analysis Institute (INSAE) list. The definition of urban areas was based on a population of at least 10,000 inhabitants or the presence of at least four of the following elements of infrastructure: a post office, an electricity network, a drinking water supply network, middle/ high school, a bank, and a representative office of the city hall.

### Data management and statistical analysis

Steps data have been managed using Epi-data 3.1. Double entry data have been obtained. For this study, the Benin Steps database was exported in ACESS 2010 software, including only current study variables. The completeness and consistency of this database was verified. Subjects for whom there were missing anthropometric data or BP were excluded from the analysis. A weight was assigned to each individual that corresponded to the inverse of the probability of inclusion in the sample using the method described by Bennett [28]. A weighted analysis of the data was performed using the survey procedure of SAS software 9.3. The Wilk-Shapiro test was performed in addition to the QQ plots to check whether the quantitative variables were normally distributed. Means were presented with their standard errors and the proportions with the 95% confidence intervals. The means of BP and blood glucose levels were calculated for the untreated subjects. Means were compared between two groups using Student's t-test and proportions using the Chi-square test.

Multivariable logistic regressions were conducted to assess the relationship between each CVRF and the area of residence, adjusting for potential confounding factors. Socio-demographic differences were identified between rural and urban populations. It was searched if these variables (age, gender, occupation) were confounders or involved in a significant interaction with the main independent variable “area of residence”. Forward method was used sequentially and manually. Age was classified in four groups (10-year intervals). The reference group was those aged 25 to 34 years. The other age groups were used as dummy variables in the model of regression. First, the main independent variable “area of residence" was included in the model. Then, other independent variables were added one by one to the model. At each step, the full and the reduced models were compared. Possible interactions between the main independent and the other independent variable were tested by including proper cross-product terms in the regression model. Likelihood ratio test was used to assess the significance of the new variable. The variable’s effect was significant if the p-value of the statistic was less than 0.05. The goodness-of-fit of the model was assessed by Hosmer and Lemeshow test (p-value of the test was upper than 0.05). The effects of variables “age” and “gender” were significant in each model except for the model regarding raised blood cholesterol. The effect of variable “occupation” was not significant. At the end of the process, the variables age and gender were retained in the fitted models. They have been forced in the model regarding area of residence and raised blood cholesterol. Crude and adjusted odds ratios (OR) were estimated and presented with their 95% confidence intervals. The analysis and presentation of results of statistical analysis are in accord with the STROBE recommendations [29].

## Results

**Fig 1:**
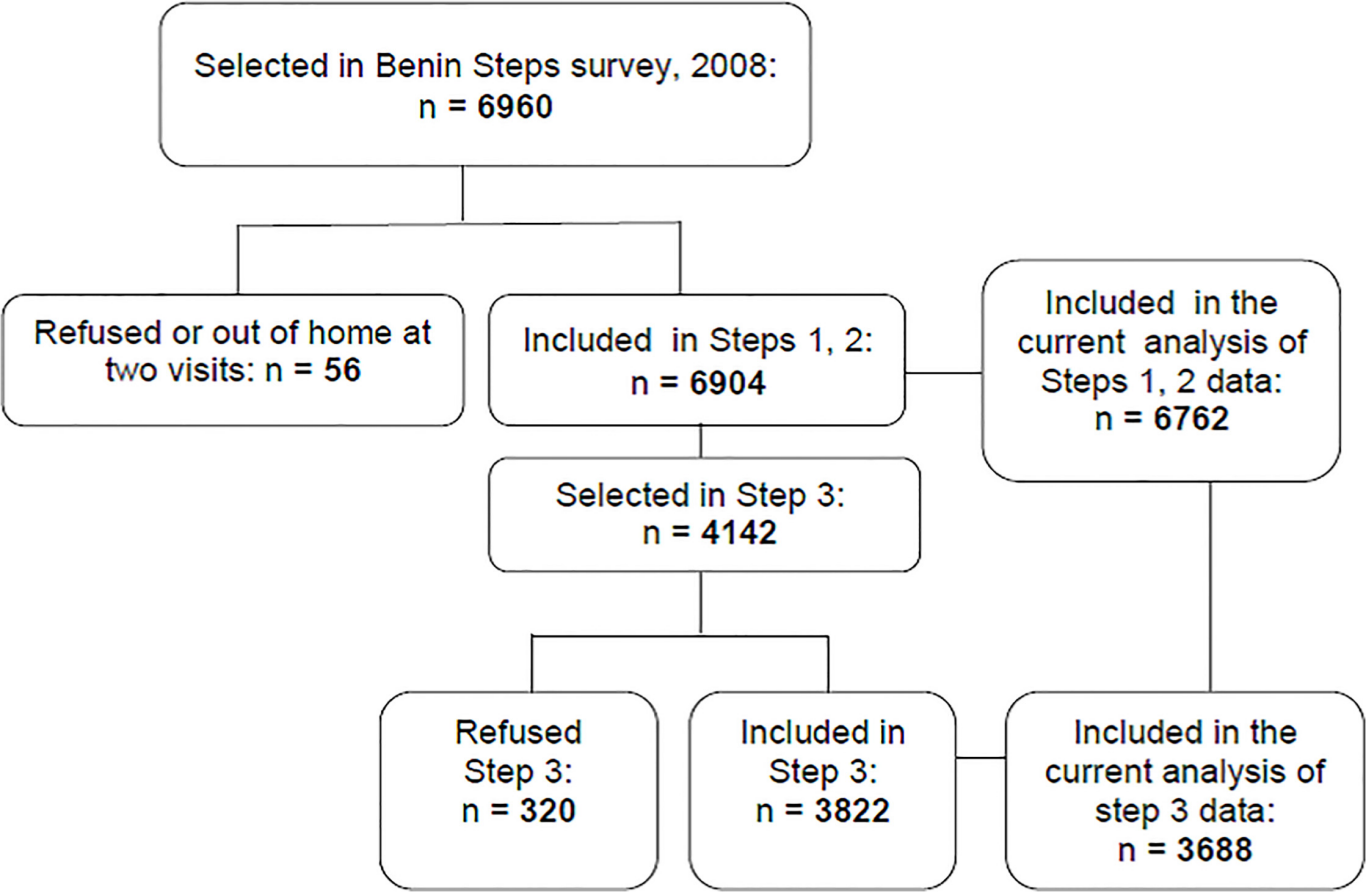
Flow chart of subjects included in Benin Steps survey and in the current analysis. Of 6960 subjects selected in the Benin Steps survey, 6904 participated, 3822 had Step 3 data, 6762 included in the current study 3688 had Step 3 data.

Of 6960 people selected in the Benin Steps survey, 6904 participated. The response rate was 99% (Fig 1). Among them, 3822 were included in Step 3. Of 6762 subjects included in the present study, 2271 were from urban areas and 4491 were from rural areas. Among them, biological data were available for 3688.

### General Characteristics

The male/female sex ratio was 1.02 (Table 1). Mean age was 42.8 ± 0.3 years. The 25–34 years age group was the most represented (44.5%). Almost two-thirds had no formal education. People working for themselves were the most numerous (78.9%).

The characteristics of the subjects included in the overall sample were comparable to those of the subjects in the Step 3 sub-sample. The sociodemographic characteristics of subjects differed depending on the area of residence. The proportion of men was significantly higher in rural than in urban areas (52.7 vs 42.7%, p<0.001). Mean age was significantly higher in rural compared to urban areas (43.5 years vs 42.8 years, p<0.001). Subjects were more likely to have no formal education in rural rather than urban areas (73.5% vs 40.9%, p<0.001). Median, monthly income of participants was 43 €, (Interquartile range, IQR: 23 €; 72 €. In urban areas, it was 54 €, (IQR: 31 €; 92 €) and in rural areas, 37 €, (IQR: 23 €; 61 €).

**Table 1:** Sociodemographic characteristic of Steps participants compared to the 25–64 years old population in Benin (Benin population from National Health statistic 2008).

Variables	Category	Steps 1,2 Sample (N = 6762)		Steps 3 Subsample (N = 3688)		25–64 years old Benin population 2008* (N = 2461543)	
		n	%	n	%	n	%
Gender	Male	3417	50.5	1797	48.7	1137208	46.2
	Female	3345	49.5	1891	51.3	1324335	53.8
Age (years)	25–34	2127	31.5	1075	29.1	1095775	44.5
	35–44	1645	24.3	906	24.6	700761	28.5
	45–54	1150	17.0	670	18.2	421481	17.1
	54–64	1840	27.2	1037	28.1	243526	9.9
Area	Urban	2271	33.6	1238	33.6	1009233	41.0
	Rural	4491	66.4	2450	66.4	1452310	59.0
Formal education	No formal schooling	4226	62.5	2278	61.8	1403080	57.0
	Primary school and above	2536	37.5	1410	38.2	1058453	43.0
Occupation	Self-employed	5333	78.9	2897	78.6	—	—
	Homemaker	635	9.4	348	9.4	—	—
	Employee	476	7.0	251	6.8	—	—
	Retired	108	1.6	72	1.9	—	—
	Apprentice/student	92	1.4	47	1.3	—	—
	Other	118	1.7	73	2.0	—	—

N: total size; n: subgroup size;

*Benin population from National Health statistic 2008

The majority of subjects (82.2%) consumed fewer than five servings of fruits and vegetables per day (Table 2).

**Table 2:** Behaviour characteristics in 25–64 years population in Benin stratified by area of residence and gender (weighted prevalences from Steps Benin 2008 sample).

Variables	Gender	Area of residence						
		Urban and Rural		Urban		Rural		
		N	% (95% CI)	n	% (95% CI)	N	% (95% CI)	p
Low fruits and vegetables intake (< 5 portions* /day)	M and F	6762	82.2 (79.9–84.4)	2371	88.8 (85.8–91.9)	4491	78.8 (75.7–81.9)	<0.001
	M	3417	82.0 (79.3–84.5)	1058	89.1 (85.2–92.9)	2359	78.7 (75.4–82.2)	<0.001
	F	3345	82.4 (79.9–84.8)	1213	88.6 (85.8–91.4)	2132	78.9 (75.4–82.3)	<0.001
Significant alcohol consumption (≥4days/week)	M and F	6762	16.4 (14.9–17.9)	2371	10.7 (9.0–12.5)	4491	19.2 (17.2–21.4)	<0.001
	M	3417	23.8 (21.6–26.0)	1058	17.2 (14.3–20.1)	2359	26.8 (23.9–29.7)	<0.001
	F	3345	8.7 (7.2–10.1)	1213	4.8 (3.4–6.3)	2132	10.8 (8.7–12.9)	<0.001
Sedentary behaviour (> 12 hours during the day)	M and F	6762	8.2 (7.0–9.4)	2371	10.2 (7.6–12.8)	4491	7.2 (5.9–8.5)	0.03
	M	3417	6.6 (5.6–7.7)	1058	8.9 (6.9–10.9)	2359	5.6 (4.4–6.8)	0.003
	F	3345	8.1 (7.5–11.6)	1213	11.4 (7.5–15.3)	2132	9.0 (7.3–10.7)	ns

*One portion = 80g; N: total size; CI: confidence interval; ns: no statistically significant difference (urban and rural proportions); M: male; F: female

Nearly a fifth (16.4%) reported significant alcohol consumption (≥ 4 days per week). Means of standard alcohol drinks were respectively 1.8 ± 0.1/day and 12.7 ± 0.4/week. Nearly one in ten (8.2%) were sedentary for more than 12 hours per day. Means of standard alcohol drinks were 1.4 ± 0.1/day and 9.9 ± 0.6/week in urban areas, and 2.1 ± 0.3/day and 9.9 ± 0.6/week in rural areas. Significant alcohol consumption was more frequent in rural compared to urban areas (p<0.001). On the other hand, sedentary behavior and low fruit and vegetable intake were significantly less frequent in rural than urban areas (respectively, p<0.001 and p<0.001).

Means of BP, anthropometric parameters and biological measures are displayed in Table 3. Means of BMI, blood glucose and blood total cholesterol levels were lower in rural than in urban areas (p<0.001, p = 0.009 and p = 0.002 respectively).

**Table 3:** Means of anthropometrics and biological parameters in 25–64 year old population in Benin, stratified by area of residence and gender.

Variables	Gender	Area of residence						
		Urban and rural		Urban		Rural		
		N	Mean (SE)	n	Mean (SE)	n	Mean (SE)	p
SBP (mm Hg)*	M and F	6471	127.9 (0.4)	2129	127.6 (0.6)	4342	128.1 (0.5)	ns
	M	3318	129.7 (0.4)	1015	129.8 (0.7)	2303	129.6 (0.5)	ns
	F	3153	126.0 (0.5)	1114	125.5 (0.6)	2039	126.3 (0.5)	ns
DBP (mm Hg) *	M and F	6471	78.6 (0.2)	2129	79.4 (0.4)	4342	78.3 (0.3)	<0.001
	M	3318	78.8 (0.3)	1015	79.7 (0.5)	2039	78.4 (0.3)	<0.001
	F	3153	78.5 (0.3)	1114	79.0 (0.4)	2039	78.2 (0.3)	ns
Pulse (beats/min)	M and F	6762	78.3 (0.2)	2271	77.9 (0.3)	4491	78.5 (0.3)	0.04
	M	3417	75.9 (0.3)	1058	75.4 (0.5)	2359	76.1 (0.4)	ns
	F	3345	80.8 (0.3)	1213	80.1 (0.3)	2132	81.1 (0.4)	0.01
BMI (kg/m^2^)	M and F	6762	23.7 (0.1)	2271	25.3 (0.2)	4491	22.8 (0.1)	<0.001
	M	3417	22.8 (0.1)	1058	23.7 (0.2)	2359	22.4 (0.1)	<0.001
	F	3345	24.5 (0.2	1213	26.8 (0.3)	2132	23.3 (0.1)	<0.001
Waist (cm)	M and F	6762	79.8 (0.2)	2271	82.4 (0.5)	4491	78.7 (0.3)	<0.001
	M	3417	79.8 (0.2)	1058	82.4 (0.5)	2359	78.7 (0.3)	<0.001
	F	3345	83.2 (03)	1213	86.9 (0.7)	2132	81.1 (0.3)	<0.001
FBG (mmol/l)*	M and F	3688	3.8 (0.1)	1238	3.9 (0.1)	2450	3.8 (0.1)	0.002
	M	1797	3.9 (0.1)	542	3.9 (0.1)	1255	3.8 (0.1)	ns
	F	1891	3.8 (0.1)	696	3.9 (0.1)	1195	3.7 (0.1)	<0.001
Blood total cholesterol (mmol/l)	M and F	3688	3.9 (0.1)	1238	4.0 (0.1)	2450	3.8 (0.1)	0.002
	M	1797	3.8 (0.1)	542	3.9 (0.1)	1255	3.8 (0.1)	ns
	F	1891	3.9 (0.1)	696	4.0 (0.1)	1195	3.8 (0.1)	0.001

(Weighted means from Steps Benin 2008 sample, N = 6762)

* Among untreated subjects; N: total size; SE: standard error; SBP: systolic blood pressure; DBP: diastolic blood pressure; BMI: body mass index; FBG: fasting blood glucose; ns: no statically difference (urban vs rural proportions); M: male; F: female

### Prevalences of CVRF in rural and urban areas in Benin

Prevalences of daily tobacco smoking and HBP were respectively, 7.7% and 2.3% (Table 4). Prevalences of obesity and raised blood glucose were respectively, 18.0% and 2.3%. Prevalence of raised serum total cholesterol was 1.6%.

Prevalences of obesity and raised blood glucose were higher in urban than in rural areas, at 16.4% vs 5.9% (p<0.001) and 3.3% vs 1.8%, (p<0.001). Conversely, the prevalence of daily tobacco smoking was higher in rural than in urban areas, at 9.3% vs 7.6% (p<0.001). There was no difference in prevalence of either HBP or raised blood total cholesterol between rural versus urban areas (respectively 29.9% vs 27.5% (p = 0.14), 1.6% vs 1.6% (p = 0.88)) (Table 4).

**Table 4:** Magnitude of cardiovascular risk factors among 25–64 year old population in Benin stratified by area of residence and gender (weighted prevalences from Steps Benin 2008 sample).

Variables	Gender	Area of residence						
		Urban and rural		Urban		Rural		
		N	% (95% CI)	n	% (95% CI)	n	% (95% CI)	p
Daily tobacco smoking (during previous 12 months)	M and F	6762	7.6 (6.8–8.5)	2271	4.3 (3.1–5.6)	4491	9.3 (8.1–10.4)	<0.001
	M	3417	13.8 (12.3–15.4)	1058	9.1 (6.5–11.6)	2359	16.1 (13.4–18.9)	<0.001
	F	3345	1.2 (0.7–1.7)	1213	0.3 (0.1–0.4)	2132	1.8 (1.1–2.6)	<0.001
HBP or currently receiving medication	M and F	6762	28.4 (26.9–29.9)	2271	29.9 (27.4–32.5)	4491	27.5 (25.6–29.5)	ns
	M	3417	28.3 (26.3–30.3)	1058	29.5 (25.7–33.4)	2359	27.8 (25.5–30.1)	ns
	F	3345	28.4 (26.6–30.2)	1213	30.4 (27.5–33.2)	2132	27.3 (24.9–29.7)	ns
Obesity (BMI ≥30 kg/m2)	M and F	6762	9.4 (8.6–10.2)	2271	16.4 (14.4–18.4)	4491	5.9 (5.1–6.7)	<0.001
	M	3417	4.6 (5.5–8.9)	1058	7.2 (5.5–8.9)	2359	3.5 (2.5–4.5)	<0.001
	F	3345	14.3 (12.8–15.9)	1213	24.6 (21.5–27.7)	2132	8.6 (7.3–9.9)	<0.001
Abdominal obesity	M and F	6762	16.1 (14.9–17.3)	2271	24.0 (21.4–26.6)	4491	12.2 (11.0–13.5)	<0.001
	M	3417	3.1 (3.4–6.1)	1058	4.8 (3.4–6.1)	2359	2.3 (1.6–3.0)	<0.001
	F	3345	29.7 (27.6–31.8)	1213	41.2 (37.3–45.1)	2132	23.3 (20.9–25.7)	<0.001
Overall obesity (BMI or abdominal criteria)	M and F	6762	18.0 (16.8–19.2)	2271	26.7 (24.0–29.3)	4491	13.6 (12.3–14.9)	<0.001
	M	3417	5.3 (4.4–6.1)	1058	8.3 (6.4–10.1)	2359	4.0 (3.0–5.0)	<0.001
	F	3345	31.1 (29.0–33.3)	1213	43.1 (39.1–47.1)	2132	24.4 (22.0–26.8)	<0.001
FBG or currently receiving medication	M and F	3688	2.3 (1.7–2.9)	1238	3.3 (2.1–4.5)	2450	1.8 (1.2–2.4)	< 0.001
	M	1797	3.0 (2.1–3.9)	542	3.7 (1.8–5.7)	1255	2.6 (1.6–3.6)	ns
	F	1891	1.7 (1.1–2.2)	696	2.9 (1.8–4.2)	1195	0.9 (0.5–1.4)	<0.001
Raised blood total cholesterol	M and F	3688	1.6 (1.2–2.0)	1238	1.6 (1.1–2.0)	2450	1.6 (1.1–2.1)	ns
	M	1797	1.5 (0.9–2.0)	542	1.3 (0.3–2.3)	1255	1.5 (0.8–2.2)	ns
	F	1891	1.7 (1.1–2.3)	696	1.8 (0.9–2.6)	1195	1.7 (0.9–2.6)	ns
HBP or currently receiving medication, and obesity	M and F	6762	7.9 (7.1–8.7)	2271	11.7 (10.3–13.2)	4491	6.0 (5.0–7.0)	<0.001
	M	3417	2.7 (2.1–3.3)	1058	4.4 (3.0–5.7)	2359	1.9 (1.3–2.6)	<0.001
	F	3345	13.4 (11.8–14.9)	1213	18.3 (15.8–20.9)	2132	10.6 (8.6–12.6)	<0.001

N: total size; CI: confidence interval; HBP: high blood pressure; BMI: body mass index; FBG: fasting blood glucose; ns: no statistically significant difference (urban vs rural proportions); M: male; F: female

However after adjustment for age and gender, the prevalence of HBP was significantly higher in urban than in rural areas (adjusted OR = 1.4, 95% CI (1.2, 1.6); p = 0.001) (Table 5). There was an independant link between each CVRF and the area of residence, with the exception of raised blood total cholesterol.

**Table 5:** Association (adjusted for age group and gender) between cardiovascular risk factors and area of residence: multivariables logistics regressions (data from Steps Benin 2008 sample).

Variables	Area of residence (reference = rural)*		
	Crude OR (95%CI)	Adjusted** OR (95%CI)	p**
Daily tobacco smoking	0.4 (0.3–0.6)	0.5 (0.3–0.7)	< 0.001
HBP or currently receiving medication	1. 1 (0.9–1.3)	1.4 (1. 2–1.6)	0.001
Obesity (BMI≥30kg/m^2^ or abdominal criteria)	2.3 (1.9–2.7)	2.6 (2.1–3.1)	< 0.001
Raised fasting blood glucose or currently receiving medication	1.9 (1.1–3.1)	2.1 (1.3–3.5)	0.004
Raised blood total cholesterol	0.9 (0.6–1.6)	1.0 (0.6–1.6)	ns

HBP: high blood pressure; BMI: body mass index; ns: no statistically significant difference; CI: confidence interval

* Rural area was reference;

** adjusted for age group and gender

## Discussion

This work was done using the databasis of the Benin Steps survey, which was carried out according to the methodology recommended by the WHO. A representative sample of the Beninese population adult aged 25 to 64 years was recruited, allowing extrapolation of the results to the Beninese population as a whole. The knowledge on the magnitude and the variations of CVRF within populations in sub-Saharan Africa, particularly in Benin, are enriched through this work. Our results could direct prevention policies at the local level. Target interventions could be proposed in Benin order to promote fruits and vegetables intake in towns and decrease tobacco use in villages. The information could also be useful in the planning of other research into CVD epidemiology in Benin.

The study showed the high prevalences of several CVRF in the adult Beninese population aged above 24 and below 65. Close to one subject in ten smoked daily, more than a quarter had HBP, and close to one subject in five was obese. Four percent of subjects had raised blood glucose, and a similar proportion had raised blood total cholesterol. Similarities have been noted between the urban and rural populations. There was no clinically relevant difference between their mean systolic and diastolic BP, nor between their mean levels of blood glucose and cholesterol. The prevalence of HBP was high in urban and rural areas.

Our results support data of epidemiological transition in sub-Saharan Africa particularly in Benin, and are consistent with the findings of Steps surveys in other countries of the same region [13]. The prevalence reported in Sub-Saharan Africa in the population aged 25 to 64 years, varied between 5% and 15% for tobacco smoking, between 25% and 50% for HBP, and between 5% and 15% for obesity [13, 27, 30–35]. Prevalences of HBP in Benin and other sub-Saharan African countries were also as high as those in developed countries.

Differences in sociodemographic characteristics (age, sex ratio) were noted between rural and urban areas. That there were fewer young people in the countryside than in towns is probably linked to the rural exodus of the young. The comparative analysis of the prevalence of CVRF according to the area of residence, adjusted for age and gender, showed some differences between urban and rural areas. The prevalences of HBP, obesity and diabetes were significantly higher in urban compared to rural areas. Conversely, the prevalences of daily tobacco smoking and excessive alcohol consumption were significantly higher in rural than urban areas. These results are similar to those of several authors who had already noted a positive gradient of prevalence of HBP, obesity and diabetes mellitus between the urban and rural areas in sub-Saharan Africa [14–18, 36]. These observations were also reported in India in a similar study in 2005 [37]. They could be linked in sub-Saharan Africa to differences in lifestyle. People in rural areas would be expected to have a traditional way of life, with a more healthy diet than among town dwellers. For example, in our study, the low intake of fruits and vegetables was less prevalent in rural than urban areas.

The higher prevalence of tobacco smoking in rural compared to urban areas does not seem to be specific to Benin [38–40]. However, environmental factors could explain the observation in Benin, such as the culture of tobacco in certain communities and the possibility of limited information about the health risks associated with tobacco. Several studies have shown that there is a relationship between quantity of alcohol consumption and atherosclerosis. Light to moderate consumption should reduce risk of atherosclerosis vascular diseases while heavy consumption may increase them [41–44]. Interventions to reduce excessive alcohol consumption could be assessed in rural communities for cardiovascular health promotion.

With regard to the prevention of HBP in this country, it would be interesting to have data on the sodium consumption of the populations studied. Interventions to reduce excessive consumption of salt in food could be assessed in these communities if necessary. The magnitude of the CVRF in Benin supports the hypothesis of a significant frequency of CVD in the Beninese population. Sufficient data on cardiovascular disease epidemiology could confirm this hypothesis.

### Strengths and limitations of the study

Some subjects were excluded from the study because of missing data which were due to errors while collection of data in the databasis or while filling questionnaire during Steps survey. Few subjects were in this case, only 2%. Hence we can consider that a selection bias is unlikely. Sociodemographic characteristics (age, gender) of subjects excluded from the study due some missing data were not different from those retained in the study. Probably there is no link between their data status and their exclusion from our analysis.

The questionnaire were in French, but were performed either in French or in local languages. Errors could have been introduced while administration of the questionnaire in local languages. This information bias could have affected study findings regarding estimation of tobacco smoking or alcohol consumption prevalence (over or under estimation). Differences in bias distribution between rural and urban groups could have affected the assessment of the relationship between tobacco use and the area of residence or alcohol consumption and the area of residence. Nevertheless this source of bias has been controled by the validation of the translation from French in local language, the choice of bilingual interviewers and the training of the interviewers.

The prevalences of HBP and raised blood glucose could have been over-estimated because these CVRF were evaluated only once (no reevaluation during another visit). These biases were limited for HBP by taking three consecutive measures according to WHO standards. Conversely, the prevalence of HBP may have been under-estimated due to restriction of measurement to a single arm, particularly among the cases of subclavian stenosis. However, given the mean age of the subjects, and taking into account the low prevalences of these anomalies in the general population in sub-Saharan Africa [32], it is reasonable to think that the impact of this source error (measurement BP to a single arm) on the results is unlikely. Analysis of the relationship between the presence of a CVRF and area of residence was limited to the comparison of prevalences between urban versus rural areas, because it was based on data from a cross-sectional study. It was not possible to take into account the duration of residence in this analysis because of the nature of data collection.

## Conclusion

Our study confirms the significant prevalence of CVRF in Benin, and notes variations in the distribution of CVRF according the area of residence. The prevalence of tobacco smoking was significantly higher in rural areas, whereas obesity and HBP predominated in towns. These data confirm the need to develop target strategies for CVRF prevention; it might be useful to consider the population characteristics in order to develop tailored interventions.

## Supporting Information

S1 Text Benin Steps survey 2008 data collection tool. (PDF)

S2 Text Benin Steps survey 2008 data collection tool coding. (PDF)

S3 Text Health ministry of Benin report of Steps survey 2008. (PDF)

S1 Datasets Database of subjects included in Benin Steps survey and in the current analysis. (XLSX)

## References

[1] Lozano R, Naghavi M, Foreman K, Lim S, Shibuya K, Aboyans V, et al Global and regional mortality from 235 causes of death for 20 age groups in 1990 and 2010: a systematic analysis for the Global Burden of Disease Study 2010. The Lancet. 2012;380(9859): 2095–2128. 10.1016/S0140-6736(12)61728-0 23245604 PMC10790329

[2] Mensah GA. Epidemiology of stroke and high blood pressure in Africa. Heart. 2008; 94(6): 697–705. 10.1136/hrt.2007.127753 18308869

[3] Mensah GA. Ischaemic heart disease in Africa. Heart. 2008; 94(7):836–843. 10.1136/hrt.2007.136523 18552223

[4] Opie LH, Seedat YK. Hypertension in sub-Saharan African populations. Circulation. 2005; 112(23): 3562–3568. 16330697 10.1161/CIRCULATIONAHA.105.539569

[5] Bosu WK. Epidemic of hypertension in Ghana: a systematic review. BMC Public Health. 2010;10: 418 10.1186/1471-2458-10-418 20626917 PMC2910685

[6] World Health Organization. The World Health Statistic 2012 Geneva, Switzerland: WHO, 2012. Available: http://www.who.int/gho/publications/world_health_statistics/FR_WHS2012_Full.pdf. Accessed 4 March 2014.

[7] Fezeu L, Kengne AP, Balkau B, Awah PK, Mbanya JC. Ten-year change in blood pressure levels and prevalence of hypertension in urban and rural Cameroon. J Epidemiol Community Health. 2010;64(4): 360–365. 10.1136/jech.2008.086355 19692732 PMC3094348

[8] Dalal S, Beunza JJ, Volmink J, Adebamowo C, Bajunirwe F, Njelekela M, et al Non-communicable diseases in sub-Saharan Africa: what we know now. Int J Epidemiol. 2011;40(4): 885–901. 10.1093/ije/dyr050 21527446

[9] Cossi MJ, Gobron C, Preux PM, Niama D, Chabriat H, Cossi M-J, Gobron C, Preux P-M, Niama D, Chabriat H, Houinato D. Stroke: Prevalence and Disability in Cotonou, Benin. Cerebrovasc Dis. 2012;33(2): 166–172. 10.1159/000334195 22222467

[10] Houinato DS, Gbary AR, Houehanou YC, Djrolo F, Amoussou M, Segnon-Agueh J, et al Prevalence of hypertension and associated risk factors in Benin. Rev Epidemiol Santé Publique. 2012;60(2): 95–102. 22436411 10.1016/j.respe.2011.09.010

[11] World Health Organization. Preventing chronic disease: a vital investment Geneva, Switzerland: WHO, 2005.

[12] Organisation mondiale de la Santé. Le Manuel de Surveillance STEPS de l’OMS: l’approche STEPWISE de l’OMS pour la surveillance des facteurs de risque des maladies chroniques Suisse, Genève: OMS, 2005.

[13] World Health Organization STEPS Country Report. Geneva, Switzerland: WHO Available: http://www.who.int/chp/steps/reports/en/. Accessed 16 January 2014.

[14] Aspray TJ, Mugusi F, Rashid S, Whiting D, Edwards R, Alberti KG, et al Rural and urban differences in diabetes prevalence in Tanzania: the role of obesity, physical inactivity and urban living. Trans R Soc Trop Med Hyg. 2000;94(6): 637–644. 11198647 10.1016/s0035-9203(00)90216-5

[15] Kasiam Lasi On'kin JB, Longo-Mbenza B, Nge Okwe A, Kangola Kabangu N. Survey of abdominal obesities in an adult urban population of Kinshasa, Democratic Republic of Congo. Cardiovasc J Afr. 2007; 18: 300–307. 17985031 PMC3975547

[16] Abubakari A, Lauder W, Agyemang C, Jones M, Kirk A, Bhopal RS. Prevalence and time trends in obesity among adult West African populations: a meta-analysis. Obes Rev. 2008;9: 297–311. 10.1111/j.1467-789X.2007.00462.x 18179616

[17] Abubakari AR, Bhopal RS. Systematic review on the prevalence of diabetes, overweight/obesity and physical inactivity in Ghanaians and Nigerians. Public Health. 2008;122(2): 173–182. 18035383 10.1016/j.puhe.2007.06.012

[18] Mathenge W, Foster A, Kuper H. Urbanization, ethnicity and cardiovascular risk in a population in transition in Nakuru, Kenya: a population-based survey. BMC Public Health. 2010;10: 569 10.1186/1471-2458-10-569 20860807 PMC2956724

[19] Nair P, Nyamphisi M, Yarnell JW. Lack of difference in blood pressure between the urban and rural population in Lesotho, Africa. Cent Afr J Med. 1994;40(10): 278–281. 7828179

[20] Mufunda J, Mebrahtu G. The prevalence of hypertension and its relationship with obesity: results from a national blood pressure survey in Eritrea. J Hum Hypertens. 2006;20: 59–66. 16151443 10.1038/sj.jhh.1001924

[21] Agyemang C. Rural and urban differences in blood pressure and hypertension in Ghana, West Africa. Public Health. 2006;120(6): 525–533. 16684547 10.1016/j.puhe.2006.02.002

[22] Damasceno A, Azevedo A, Silva-Matos C, Prista A, Diogo D, Lunet N. Hypertension prevalence, awareness, treatment, and control in Mozambique: urban/rural gap during epidemiological transition. Hypertension. 2009;54(1): 77–83. 10.1161/HYPERTENSIONAHA.109.132423 19470872

[23] Institut national de la statistique et de l’analyse économique du Bénin. Résultats provisoires du 4e recensement général de la population et de l’habitat au Bénin en 2013 Cotonou, Bénin: INSAE, 2013 Avalaible: www.insae-bj.org/recensement-population.html. Accessed 2014 Apr 10.

[24] Institut national de la statistique et de l’analyse économique (INSAE). Enquête modulaire intégrée sur les conditions de vie des ménages, 2è édition (EMICOV 2011): principaux indicateurs Cotonou, Bénin: INSAE, 2011.

[25] Ministère de la santé du Bénin. Annuaire des statistiques 2010 Cotonou, Bénin: MS, 2011.

[26] Berthier C, Caron N, Neros B. La méthode de Kish: les problèmes de réalisation du tirage et de son extrapolation INSEE, France, Série Méth Stat 1998;n89810.

[27] Akoha E. Prévalence de l’hypertension artérielle en population générale à Cotonou en 2007. Thèse de médecine, Faculté des Sciences de la Santé, Cotonou, Bénin, 2007;no 1367: 79p.

[28] Bennett S, Woods T, Liyanage W, Smith D. A simplified general method for cluster-sample surveys of health in developing countries. World Health Statist Quart. 1991;44: 98–106. 1949887

[29] Von Elm E, Altman DG, Egger M, Pocock SJ, Gøtzsche PC, Vandenbroucke JP, et al The Strengthening the Reporting of Observational Studies in Epidemiology (STROBE) statement: guidelines for reporting observational studies. The Lancet. 2007; 370(9596): 1453–1457. 18064739 10.1016/S0140-6736(07)61602-X

[30] Sobngwi E, Mbanya JC, Unwin NC, Kengne AP, Fezeu L, Minkoulou EM, et al Physical activity and its relationship with obesity, hypertension and diabetes in urban and rural Cameroon. Int J Obes Relat Metab. Disord 2002; 25: 1009–1016. 10.1038/sj.ijo.0802008 12080456

[31] Msyamboza KP, Ngwira B, Dzowela T, Mvula C, Kathyola D, Harries AD, et al The burden of selected chronic non-communicable diseases and their risk factors in Malawi: nationwide STEPS survey. PLoS One. 2011;6(5): e20316 10.1371/journal.pone.0020316 21629735 PMC3100352

[32] Baragou S, Djibril M, Atta B, Damorou F, Pio M, Balogou A. Prevalence of Cardiovascular Risk Factors in an Urban Area of Togo: A WHO STEPS-Wise Approach in Lome, Togo. Cardiovascular Journal of Africa. 2012; 23(6): 309–312. 10.5830/CVJA-2011-071 22836151 PMC3734750

[33] Ngoungou EB, Aboyans V, Kouna P, Makandja R, Ecke Nzengue JE, Allogho CN, et al Prevalence of cardiovascular disease in Gabon: A population study. Arch Cardiovasc Dis. 2012;105(2): 77–83. 10.1016/j.acvd.2011.12.005 22424325

[34] Adebayo RA, Balogun MO, Adedoyin RA, Obashoro-John OA, Bisiriyu LA, Abiodun OO, et al Prevalence of Hypertension in Three Rural Communities of Ife North Local Government Area of Osun State, South West Nigeria. Int J Gen Med 2013;6: 863–868. 10.2147/IJGM.S51906 24348064 PMC3857150

[35] Pessinaba S, Mbaye A, Yabeta GA, Kane A, Ndao CT, Ndiaye MB, et al « Prevalence and Determinants of Hypertension and Associated Cardiovascular Risk Factors: Data from a Population-Based, Cross-Sectional Survey in Saint Louis, Senegal ». Cardiovascular Journal of Africa. 2013;24(5): 180–183. 10.5830/CVJA-2013-030 24217165 PMC3748453

[36] Chow CK, Teo KK, Rangarajan S, Islam S, Gupta R, Avezum A, et al Prevalence, Awareness, Treatment, and Control of Hypertension in Rural and Urban Communities in High-, Middle-, and Low-Income Countries. JAMA. 2013;310 (9): 959–968. 10.1001/jama.2013.184182 24002282

[37] Mohan V, Mathur P, Deepa R, Deepa M, Shukla DK, Menon GR, et al Urban rural differences in prevalence of self-reported diabetes in India—the WHO-ICMR Indian NCD risk factor surveillance. Diabetes Res Clin Pract. 2008;80(1): 159–168. 10.1016/j.diabres.2007.11.018 18237817

[38] Mumford EA, Levy DT, Gitchell JG, Blackman KO. Smokeless tobacco use 1992–2002: trends and measurement in the Current Population Survey-Tobacco Use Supplements. Tob Control. 2006;15(3): 166–171. 16728746 10.1136/tc.2005.012807 PMC2564653

[39] Vander Weg MW, Cunningham CL, Howren MB, Cai X. Tobacco use and exposure in rural areas: Findings from the Behavioral Risk Factor Surveillance System. Addict Behav. 2011;36(3): 231–236. 10.1016/j.addbeh.2010.11.005 21146318

[40] Almeida L, Szklo A, Sampaio M, Souza M, Martins LF, Szklo M, et al Global Adult Tobacco Survey Data as a Tool to Monitor the WHO Framework Convention on Tobacco Control (WHO FCTC) Implementation: The Brazilian Case. Int J Environ Res Public Health. 2012;9(7): 2520–2536. 10.3390/ijerph9072520 22851957 PMC3407918

[41] Mukamal KJ, Rimm EB. Alcohol's Effects on the Risk for Coronary Heart Disease. Alcohol Res Health. 2001;25(4): 255–261. 11910702 PMC6705710

[42] Rouillier P, Boutron-Ruault MC, Bertrais S, Arnault N, Daudin JJ, Bacro JN, et al Alcohol and Atherosclerotic Vascular Disease Risk Factors in French Men: Relationships Are Linear, J-Shaped, and U-Shaped. Alcohol Clin Exp Res. 2005;29(1): 84–88 15654296 10.1097/01.alc.0000150005.52605.fa

[43] Pletcher MJ, Varosy P, Kiefe CI, Lewis CE, Sidney S, Hulley SB. Alcohol Consumption, Binge Drinking, and Early Coronary Calcification: Findings from the Coronary Artery Risk Development in Young Adults (CARDIA) Study. Am J Epidemiol. 2005;161(5): 423–433. 15718478 10.1093/aje/kwi062

[44] Brien SE, Ronksley PE, Turner BJ, Mukamal KJ, Ghali WA. Effect of alcohol consumption on biological markers associated with risk of coronary heart disease: systematic review and meta-analysis of interventional studies. BMJ. 2011;342: d636 10.1136/bmj.d636 21343206 PMC3043110

