# Supplementary material for: Magnitude of Cardiovascular Risk Factors in Rural and Urban Areas in Benin: Findings from a Nationwide Steps Survey
Source: PLoS One. 2015 May 6;10(5):e0126441. doi: 10.1371/journal.pone.0126441 (PMC4422555; doi:10.1371/journal.pone.0126441)
Supplement: S1 Text — (PDF) [file pone.0126441.s001.pdf]

Organisation Mondiale de la Santé

L'approche STEPwise sur les facteurs de risque des MNT

Instrument de saisie des données STEPS v2.0, STEPS BENIN 2008

ID : Numéro d'Identification du répondant ###########

Section I: Information sur le lieu et la date

I1 Code du district ###

I2 Nom du centre/village ______________________________

I3 Code du centre/village (1. urbain/2. rural) #

Depart : département

STEP 1: QUESTIONNAIRE

INFORMATION DEMOGRAPHIQUE

C1 Sexe (1. Masculin, 2. Féminin) #

C2 Quelle est votre date de naissance ?

<dd/mm/yyyy>

C3 Quel âge avez-vous ? ##

C4 En tout, combien d'années passé à l'école ou à suivre formation ? ##

C6 Quel est le plus haut niveau d'instruction que vous ayez atteint ? ##

1 "Aucune instruction officielle"
 2 "Moins que l'école primaire"
 3 "Fin de l'école primaire"
 4 "Fin de l'école secondaire"
 5 "Fin du lycée ou équivalent"
 6 "École supérieure, Université"
 7 "Diplôme post-universitaire obten"
 77 "ne sait pas"
 88 refuse
 99 "réponse manquante"

C7 Laquelle des catégories suivantes décrit le mieux votre activité prof. ? ##

 1 "Employé(e) de l'État"
 2 "Employé(e) dans le privé"
 3 Indépendant(e)
 4 Bénévole
 5 Étudiant(e)
 6 "Maître(sse de maison"
 7 Retraité(e)
 8 Chômeur(se)
 9 Invalide
 10 Autres
 77 "ne sait pas"
 88 refuse
 99 "réponse manquante"

C9(a-d) En prenant comme référence l'année passée, pouvez dire revenus moyens ?

C9a	Par semaine	######
C9b	ou par mois	######
C9c	ou par an	######
C9d	Refuse	##

C10 Si vous ne connaissez pas le montant, pouvez donner estimation revenu ##

 1 "<= 66.000"
 2 [67.000-132.000]
 3 [133.000-198.000]
 4 [199.000-264.000]
 5 "> 264.000"
 77 "ne sait pas"
 88 refuse
 99 "réponse manquante"

CONSOMMATION DE TABAC

T1 Fumez-vous actuellement des produits à base du tabac? ##
T2 Si oui, fumez-vous quotidiennement ? ##
T3 A quel âge avez-vous commencé à fumer quotidiennement ? ##
Si vous ne vous rappelez pas à quel âge, souvenez-vous depuis quand vous avez commencé à fumer quotidiennement ?

T4a	En années	##
T4b	En mois	##
T4c	En semaines	##

Quelle quantité des produits suivants fumez-vous en moyenne chaque jour ?

T5a	Cigarettes industrielles	##
T5b	Cigarettes roulées	##
T5c	Pipes	##
T5d	Cigares/cheroots	##
T5e	Autre	##
T5other	___________________________	

T6 Dans le passé, avez-vous déjà fumé quotidiennement ? ##
T7 Quel âge aviez-vous quand vous avez arrêté de fumer quotidiennement ? ##

Si vous ne vous rappelez pas de l'âge, souvenez-vous depuis quand vous avez arrêté de fumer quotidiennement ?

T8a	En années	##
T8b	En mois	##
T8c	En semaines	##

T9 Consommez-vous actuellement du tabac non fumé ? ##
T10 Si Oui: En consommez-vous quotidiennement ? ##

En moyenne, combien de fois par jour prenez-vous les produits indiqués:

T11a	Tabac à priser (voie orale)	##
T11b	Tabac à priser (voie nasale)	##
T11c	Tabac à mâcher	##
T11d	Feuilles de bétel	##
T11e	Autre	##
T11Other	___________________________	

T12 Dans le passé, avez-vous déjà pris quotidiennement du tabac non fumé ? ##

CONSOMMATION D'ALCOOL

A1 Avez-vous consommé une boisson alcoolisée dans les 12 derniers mois ? ##
A2 Durant les douze derniers mois, à quelle fréquence boisson alcoolisée ? ##
1 Quotidiennement
2 "5-6 jours par semaine"
3 "1-4 jours par semaine"
4 "1-3 jours par mois"
5 "Moins d'1 fois par mois"
7 "ne sait pas"
8 refuse
9 "réponse manquante"
A3 Quand vous buvez de l'alcool, combien de verres en moyenne par jour ? ##
A4 Avez-vous consommé une boisson alcoolisée ces trente derniers jours ? ##

A5(a-g) Au cours des 7 derniers jours, combien de verres standard d'alcool?

A5a	Lundi	##
A5b	Mardi	##
A5c	Mercredi	##
A5d	Jeudi	##
A5e	Vendredi	##
A5f	Samedi	##
A5g	Dimanche	##

A6 Dans les 12 derniers mois, le plus grand nombre de verres ? ##
A7 Homme: Combien de jours avez-vous consommé 5 verres ou + ? ###
A8 Femme: Combien de jours avez vous consommé 4 verres ou + ? ###

```
--------------------------------------------------------------------------------
HYGIENE ALIMENTAIRE

D1 Habituellement, au cours d'une semaine,combien de jours consommer fruits ?  ##
D2 Combien de portions de fruits mangez-vous lors d'une de ces journées ?      ##
D3 Habituellement, au cours d'une semaine,combien de jours consommer légumes ? ##
D4 Combien de portions de légumes mangez-vous lors d'une de ces journées ?     ##
--------------------------------------------------------------------------------
SEDENTARITE
 Combien de temps passez-vous en position assise lors d'une journée habituelle ?
        P16a                                        heures                    ##
        P16b                                        minutes                   ##
--------------------------------------------------------------------------------
ANTECEDENTS DE LA TENSION ARTERIELLE (TA)
H2 Est-ce qu'un profesionnel vous a dit que vous aviez TA élevée ?            ##

   H3(a-e) Suivez-vous actuellement les traitements suivants ?
H3a                             Médicaments                                   ##
H3b                             Régime spécial prescrit                       ##
H3c                             Conseil pour perdre du poids                  ##
H3d                             Conseil pour arrêter de fumer                 ##
H3e                             Conseil pour activité physique                ##


ANTECEDENTS DE DIABETE
H6 A-t-on mesuré votre glycémie ces 12 derniers mois ?                        ##
H7 Est-ce qu'un profesionnel vous a déjà dit que avez du diabète ?            ##

   H8(a-f)  Suivez-vous actuellement les traitements suivants ?
H8a                            Insuline                                       ##
H8b                            Traitement oral                                ##
H8c                            Régime spécial prescrit                        ##
H8d                            Conseil pour perdre du poids                   ##
H8e                            Conseil pour arrêter de fumer                  ##
H8f                            Conseil pour activité physique                 ##
--------------------------------------------------------------------------------
                             STEP 2:  MESURES PHYSIQUES
--------------------------------------------------------------------------------
POIDS ET TAILLE
M3   Taille                                                                ###.#
M4   Poids                                                                 ###.#

TOUR DE TAILLE
M7   Tour de taille                                                        ###.#

PRESSION ARTERIELLE
M11a Mesure systolique 1                                                   ###
M11b Mesure diastoloque 1                                                  ###
M12a Mesure systolique 2                                                   ###
M12b Mesure diastoloque 2                                                  ###
M13a Mesure systolique 3                                                   ###
M13b Mesure diastoloque  3                                                 ###
M14  Suivi d'un traitement?                                                ##

TOUR DE HANCHE
M15  Tour de hanche                                                        ###.#

RYTHME CARDIAQUE
M16a  Mesure 1                                                             ###
M16b  Mesure 2                                                             ###
M16c  Mesure 3                                                             ###
--------------------------------------------------------------------------------
                             STEP 3:  MESURES BIOCHIMIQUES
--------------------------------------------------------------------------------
B5   Glycémie à jeun                  ##.##
B7   Cholesterol Total                ##.##
```
