# Supplementary material for: Magnitude of Cardiovascular Risk Factors in Rural and Urban Areas in Benin: Findings from a Nationwide Steps Survey
Source: PLoS One. 2015 May 6;10(5):e0126441. doi: 10.1371/journal.pone.0126441 (PMC4422555; doi:10.1371/journal.pone.0126441)
Supplement: S2 Text — (PDF) [file pone.0126441.s002.pdf]

REPUBLIQUE DU BENIN

World Health Organization

MINISTERE DE LA SANTE

Direction Nationale de la Protection Sanitaire

Programme National de Lutte contre les Maladies Non Transmissibles

RAPPORT FINAL DE L'ENQUETE STEPS AU BENIN

Juin 2008

EQUIPE DE REDACTION

Pr. HOUINATO Dismand	Coordonnateur National / PNLMNT
Dr SEGNON AGUEH Judith A.	Médecin Epidémiologiste / PNLMNT
Pr. DJROLO François	Point focal diabète /PNLMNT
Dr DJIGBENNOUDE Oscar	Médecin Santé Publique/ PNLMNT

Sommaire

RESUME 1
1 INTRODUCTION 2
2 OBJECTIFS 5
3 CADRE DE L'ETUDE: (étendue géographique) 7
4 METHODE 16
5 RESULTATS 23
6 Références bibliographiques 83
7 Annexes

Liste des tableaux

Tableau I: Caractéristiques sociodémographiques des sujets enquêtés au Bénin en 2008..... 23
Tableau II: Répartition des sujets enquêtés en fonction de leur niveau d'instruction, activité professionnelle et département au Bénin en 2008. 24
Tableau III : Répartition des consommateurs de tabac par tranche d'âge au Bénin en 2008. 26
Tableau IV: Répartition des consommateurs de tabac en fonction de leur fréquence de consommation et de leur sexe au Bénin en 2008.. 27
Tableau V: Répartition des consommateurs de tabac fumé par tranche d'âge au Bénin en 2008... 27
Tableau VI : Nombre moyen de cigarettes fumées par jour au Bénin en 2008. 28
Tableau VII: Répartition des consommateurs de tabac non fumé par tranche d'âge au Bénin en 2008. 29
Tableau VIII: Répartition des consommateurs de tabac en fonction du niveau d'instruction, de l'appartenance socioculturelle et du milieu de provenance au Bénin en 2008. 30
Tableau IX: Répartition des consommateurs de tabac par département au Benin en 2008. .. 31
Tableau X : Répartition des sujets selon leur consommation d'alcool au cours des douze derniers mois en fonction de l'âge au Bénin en 2008. 32
Tableau XI: Répartition des consommateurs quotidiens d'alcool par sexe au cours des douze derniers mois au Bénin... 33
Tableau XII: Répartition des consommateurs d'alcool par sexe et par fréquence de consommation au cours des douze derniers mois au Bénin. 33
Tableau XIII : Répartition des consommateurs actuels selon l'importance de la consommation au cours de la semaine écoulée en fonction de l'âge au Bénin en 2008.......... 35
Tableau XIV: Répartition des consommateurs actuels par degré du risque (dangereux ou nocif) en fonction de l'âge au cours des sept derniers jours, Bénin, 2008............................. 36
Tableau XV: Répartition des sujets ayant une consommation nocive d'alcool en fonction du niveau d'instruction, de l'appartenance socioculturelle et du milieu de provenance au Bénin en 2008. 39
Tableau XVI: Répartition des sujets ayant une consommation nocive d'alcool par département au Bénin en 2008. 40
Tableau XVII: Consommation nocive d'alcool chez les consommateurs de tabac et les non consommateurs de tabac. 40
Tableau XVIII: consommation de tabac chez les sujets ayant une consommation nocive d'alcool et chez les abstinents. 41
Tableau XIX : Répartition des sujets selon le niveau d'activité et l'âge au Bénin en 2008... 42
Tableau XX: Répartition des sujets ayant un niveau d'activité physique limite selon le département au Bénin en 2008 43
Tableau XXI : Répartition des sujets selon la pratique de l'activité physique au travail, le sexe et l'âge au Bénin en 2008 44
Tableau XXII : Répartition des sujets qui pratiquent une activité physique au travail en fonction du lieu de provenance au Bénin en 2008... 44

Tableau XXIII: Répartition des sujets selon la pratique de l'activité physique pour les déplacements, le sexe et l'âge au bénin en 2008. 45
Tableau XXIV: Répartition des sujets inactifs aux déplacements selon le lieu de provenance au Bénin en 2008 45
Tableau XXV: Répartition des sujets selon la pratique ou non de l'activité physique pour les loisirs, le sexe et l'âge au Bénin en 2008 46
Tableau XXVI: Répartition des sujets qui pratiquent une activité physique au loisir en fonction du lieu de provenance au Bénin en 2008 46
Tableau XXVII : Temps moyen en minutes consacre a chaque type d'activité physique selon l'âge au bénin en 2008 47
Tableau XXVIII : Temps moyen consacré chaque jour à des comportements sédentaires selon le sexe et l'âge au Bénin en 2008 49
Tableau XXIX : Répartition des sujets selon la consommation insuffisante de fruits et de légumes et l'âge au Bénin en 2008 50
Tableau XXX : Répartition des sujets selon la consommation de fruits et légumes et le département au Bénin en 2008 51
Tableau XXXI: Répartition des sujets selon la consommation de fruits et légumes et le lieu de provenance au Bénin en 2008 51
Tableau XXXII : Répartition des sujets selon la consommation insuffisante de fruits et légumes et le niveau d'instruction au Bénin en 2008 52
Tableau XXXIII : Répartition des sujets selon la consommation insuffisante de fruits et légumes et l'ethnie au Bénin en 2008 52
Tableau XXXIV : Répartition des sujets selon la consommation insuffisante de fruits et de légumes et la catégorie professionnelle au Bénin en 2008 53
Tableau XXXV: Nombre moyen de portion de fruits et/ ou de légumes consommés par jour selon l'âge au Bénin en 2008 54
Tableau XXXVI : Nombre moyen de jours de consommation de fruits ou de légumes par semaine selon l'âge au Bénin en 2008 54
Tableau XXXVII : Prévalence de la TA élevée en fonction du sexe, du milieu de résidence et de l'âge au Bénin en 2008 55
Tableau XXXVIII : Prévalence de la tension artérielle élevée en tenant compte de la prise d'antihypertenseur en fonction de l'âge au Bénin en 2008 56
Tableau XXXIX : prévalence de la tension artérielle élevée en fonction du niveau d'instruction, de l'activité professionnelle et de l'ethnie au Bénin en 2008 57
Tableau XL : prévalence de la tension artérielle élevée en fonction des différents départements du Bénin en 2008 58
Tableau XLI : Répartition des sujets enquêtés selon les différents stades de la TA élevée de l'OMS au Bénin en 2008 58
Tableau XLII: Répartition des sujets ayant une TA élevée en fonction de la consommation de fruits et légumes (FL) au Bénin en 2008 59
Tableau XLIII : Répartition des sujets ayant une TA élevée en fonction de la consommation nocive d'alcool au Bénin en 2008 59
Tableau XLIV: Répartition des sujets ayant une TA élevée en fonction de l'obésité au Bénin en 2008 60

Tableau XLV: Répartition des sujets enquêtés ayant une TA élevée en fonction de leurs antécédents au Bénin en 2008 61
Tableau XLVI: Prévalence de l'obésité en fonction de l'âge au Bénin en 2008 62
Tableau XLVII: prévalence de l'obésité en fonction du niveau d'instruction, de l'activité professionnelle et de l'ethnie au Bénin en 2008 63
Tableau XLVIII: prévalence de l'obésité en fonction des départements au Bénin en 2008 .. 64
Tableau XLIX: prévalence de la surcharge pondérale en fonction de l'âge au Bénin en 2008 65
Tableau L: Prévalence de la surcharge pondérale en fonction de l'activité professionnelle et de l'ethnie au Bénin en 2008 66
Tableau LI: Prévalence de l'hyperglycémie et de la glycémie anormale a jeun en fonction de l'âge au Bénin en 2008 69
Tableau LII : Prévalence de la glycémie à jeun anormale et de l'hyperglycémie de type diabétique en fonction du département au Bénin en 2008. 70
Tableau LIII: Prévalence de la glycémie anormale à jeun et de l'hyperglycémie de type diabétique au Bénin en 2008 71
Tableau LIV: Prévalence de la glycémie à jeun anormale et de l'hyperglycémie de type diabétique selon le niveau d'instruction au bénin en 2008. 72
Tableau LV: Prévalence de la glycémie à jeun anormale et de l'hyperglycémie de type diabétique selon l'activité professionnelle au Bénin en 2008. 73
Tableau LVI: Prévalence de l'hypercholestérolémie et de cholestérolémie totale élevée en fonction de l'âge au Bénin en 2008 74
Tableau LVII: Prévalence l'hypercholestérolémie par département au Bénin en 2008 75
Tableau LVIII: Prévalence de l'hypercholestérolémie au Bénin selon l'ethnie 76
Tableau LIX: Prévalence de l'hypercholestérolémie selon le niveau d'instruction au Bénin en 2008 76
Tableau LX: Prévalence de l'hypercholestérolémie selon l'activité professionnelle 77

LIiste des figures

Figure 1: Prévalence de la consommation d'alcool par niveau de risque au cours des sept derniers jours. 36
Figure 2 : Répartition des sujets enquêtés selon qu'ils aient reçu des conseils relatifs au mode de vie par un médecin ou un agent de santé pour une TA élevée.. 60
Figure 3: Répartition des sujets enquêtés en fonction de l'indice de masse corporelle au Bénin en 2008.. 67

RESUME

Les pays en développement sont confrontés à l'émergence des Maladies Non Transmissibles (MNT) dont les conséquences sont très importantes en termes de morbidité et de mortalité. Le rôle prépondérant de l'accroissement de certains facteurs de risque communs à ces affections n'est plus à démontrer. Face à cette situation, l'absence de données récentes et de système de surveillance concernant les facteurs de risque communs aux MNT, le Bénin se propose de réaliser l'enquête nationale STEPS recommandée par l'OMS. L'objectif général est d'étudier les facteurs de risque communs aux MNT.

L'étude explorera le STEPS 1, le STEPS 2 et le STEPS 3. Il s'agit d'une étude transversale, descriptive et analytique couvrant une période de 2 mois. Elle s'est déroulée dans les douze départements du Bénin et a porté sur une population d'étude composée d'adultes de 25 à 64 ans. Le sondage en grappes à plusieurs degrés proposé par l'OMS a été adopté en supposant que la population d'étude est homogène. La base de sondage est constituée par les listes des 12 départements et des 546 arrondissements du Bénin. La sélection des participants dans les ménages a été faite selon la méthode Kish recommandée par l'OMS. L'outil de collecte est constitué de l'instrument STEPS proposé par l'OMS. La saisie et l'analyse des données ont été faite de façon standardisée selon les recommandations du STEPS.

Au terme de l'étude, une prévalence des facteurs de risque comportementaux et biologiques des MNT est connue.

1 INTRODUCTION

L'Afrique, qui continue de supporter le poids impressionnant des maladies infectieuses, se trouve confrontée, à une explosion des maladies non transmissibles (MNT) depuis quelques décennies. Le rôle prépondérant des facteurs de risque n'est plus à démontrer dans la survenue des MNT. De nos jours, l'urbanisation des pays en développement et la mondialisation du marché alimentaire contribuent aux changements de comportement et du mode de vie des populations. Ce changement du mode de vie entraîne l'émergence et l'aggravation de ces facteurs de risque. Le seul moyen pour y remédier est la lutte contre ces facteurs de risque communs aux MNT. Les conséquences de ces MNT sont très importantes en termes de morbidité et de mortalité. Selon l'Organisation Mondiale de la Santé (OMS), les MNT sont responsables de 60% de décès et de 47% de la charge mondiale de morbidité [1].

En 2001, plus de 16,5 millions de décès dus aux maladies cardiovasculaires (MCV) ont été répertoriés dans le monde et 80% de ces décès se retrouvent dans les pays en développement (PED) [2]. Il est évident que la charge de morbidité due aux MNT aura des conséquences considérables au niveau social et économique. Selon les projections de l'OMS dans son rapport 2003, les MCV constitueront la première cause de mortalité d'ici l'an 2010 [1]. Aussi, une projection de la Banque Mondiale montre t-elle qu'en Afrique, le pourcentage de décès dû aux seuls désordres cardio-circulatoires passera de 19% en 1995 à 35% en 2015 [3].

Par ailleurs selon les estimations de l'OMS, le nombre d'adultes affectés par le diabète sucré augmentera de 170% dans les pays en développement versus 41% dans les pays développés entre 1995 à 2025 [4].

Au Bénin, diverses enquêtes ont été réalisées sur les facteurs de risque des MNT. Ainsi en 2001, la prévalence des facteurs de risque [5] était de :

- 13,6 à 20,2 % pour l'HTA
- 2,9 % pour le diabète.
- 7,5 à 19 % pour la consommation de tabac
- 10 à 20 % pour l'obésité.
- environ 20% des adultes présentent une cholestérolémie totale supérieure à 2,50 g/l.

En 2007, dans le département du Littoral, elle était de :

- 27,3 % pour l'HTA ;
- 4,6 % pour le diabète ;
- 30 % pour l'obésité chez les femmes ;
- 2,6 % pour le tabagisme ;

Au vu de ces données, le Bénin n'est guère épargné de cet accroissement des MNT. Or, la prise en charge onéreuse de ces maladies grève les dépenses de santé individuelles et collectives, dégradant davantage les conditions de vie déjà précaires de la population béninoise.

En l'absence de données récentes et de système de surveillance concernant les facteurs de risque communs aux MNT, le Bénin se propose de réaliser l'enquête nationale STEPS recommandée par l'OMS suite à l'Assemblée Mondiale de la Santé de Mai 2000. En effet, au cours de cette Assemblée, la résolution WHA/53.17 sur la stratégie mondiale de prévention intégrée des maladies non transmissibles a été adoptée ; elle s'appuie essentiellement sur l'identification et la réduction du niveau d'exposition des populations aux facteurs de risque de ces maladies chroniques.

Au Bénin, il existe déjà une structure à partir de laquelle l'enquête STEPS pourrait être développée. En effet, depuis Janvier 2007, il a été créé un Programme National de Lutte contre les Maladies Non Transmissibles (PNLMNT) animé par du personnel qualifié, compétent et disponible. De plus, un groupe de professionnels de santé a été formé sur la planification du

protocole STEPS de l'OMS, ces derniers n'attendent que l'étape de la mise en œuvre pour mettre les compétences acquises au service du PNLMNT. Des ressources matérielles pourront être obtenues grâce à l'appui de l'OMS (Tensiomètre, pèse-personne, ruban métrique, Accutrend, toise, etc.)

L'importance de la mise en place d'un système de surveillance des facteurs de risque des MNT n'est plus à démontrer, elle permettra de:

- constituer une base de données sur les facteurs de risque des MNT au Bénin ;
- disposer d'arguments irréfutables pour un plaidoyer en direction des décideurs pour une meilleure allocation de ressources pour la prévention et la lutte contre ces maladies ;
- déterminer les groupes à risque, cibles des campagnes d'IEC/CC (Information, Education et Communication pour un changement de comportement) ;
- suivre dans le temps l'évolution des facteurs de risque des MNT au Bénin ;
- prévenir les épidémies de ces maladies ;
- élaborer un plan stratégique de lutte contre les MNT ;
- évaluer à long terme, les interventions sanitaires mises en œuvre à l'issue des premières enquêtes sur les facteurs de risque des MNT ;
- aider les services de santé à déterminer et planifier les priorités de santé en matière de MNT.

2 OBJECTIFS

2.1 Objectif général

Etudier les facteurs de risque communs aux MNT.

2.2 Objectifs spécifiques

- Déterminer la prévalence des facteurs de risque comportementaux.
- Déterminer la prévalence des facteurs de risque biologiques.
- Identifier les facteurs associés à la survenue des maladies non transmissibles.

CADRE DE L'ETUDE

3 CADRE DE L'ETUDE: (étendue géographique)

Notre enquête s'est déroulée sur toute l'étendue du territoire du Bénin.

3.1 Description et caractéristique du département de l'Atacora

3.1.1 Présentation du département

Situé dans la partie septentrionale à l'extrême Ouest du Bénin, le département de l'Atacora compte neuf communes que sont : Natitingou (Chef-lieu du Département), Kérou, Kouandé, Péhunco, Cobly, Boukoumbé, Matéri, Toucountouna, Tanguiéta et regroupe 384 villages. Avec une superficie totale de 20499 km2, l'Atacora est le troisième département par son étendue, après l'Alibori (26 242 km2) et le Borgou (25 856 km2).

3.1.2 Les infrastructures sociocommunautaires

La couverture sanitaire des villages en termes de présence d'infrastructures sanitaires est mal assurée. En effet, pour 384 villages que compte le département, on a dénombré : 21 dispensaires, 27 maternités, 40 complexes de santé.

L'accès à l'eau potable semble poser encore d'énormes difficultés aux populations.

Sur le plan des infrastructures scolaires, on note 369 écoles primaires, 10 collèges à 1er cycle et 2 à 2ème cycle.

3.2 Description et caractéristique du département de la Donga

3.2.1 Présentation du département de la Donga

Le département de la Donga occupe la zone sud de l'ancien département de l'Atacora. Il s'étend sur une superficie de 11 126 km2. Il compte 177 villages ou quartiers de ville répartis dans quatre communes à savoir les communes de Djougou, de Bassila, de Copargo et de Ouaké. Le département de la Donga est composé de 26 arrondissements.

3.2.2 Infrastructures sociocommunautaires

Que se soit sur le plan sanitaire, de l'accès à l'eau potable ou de l'éducation, le département de la Donga a encore beaucoup de défis à relever.
On y dénombre pour 177 villages: 14 maternités, 21 Centres de Santé, 15 dispensaires.

Pour ces 177 villages, on compte 246 écoles primaires, 13 collèges à 1er cycle et 2 à 2ème cycle.

3.3 Description et caractéristique du département de l'Atlantique

3.3.1 Présentation du département

Le département de l'Atlantique est l'un des plus petits des douze (12) départements du Bénin et s'étend sur près de 100 km de la côte vers l'intérieur du pays. Il a une superficie de 3 233 km² et regroupe 500 villages et huit communes que sont Abomey-Calavi, Allada, Kpomassè, Ouidah, So-Ava, Toffo, Torri-Bossito et Zè.

3.3.2 Infrastructures socio- communautaires

L'accès à l'eau potable constitue encore un véritable problème. En effet, sur 500 villages que compte le département, moins d'un tiers (150) seulement dispose d'adduction d'eau et de forages publics.
Au plan sanitaire, pour cinq cents (500) villages regroupés au sein de huit communes on dénombre : 61 Centres de Santé, 15 maternités, et 12 dispensaires ;

Sur le plan de l'éducation, on dénombre 487 écoles primaires, 51 collèges à 1er cycle et 11 à 2ème cycle. Rapportées à l'effectif de la population du département, ces infrastructures laissent encore des disparités à combler. Il faut noter que le département abrite l'Université d'Abomey- Calavi.

3.4 Description et caractéristique du département du Littoral

3.4.1 Présentation du département

Le département du Littoral est la ville de Cotonou érigée en département par le dernier découpage administratif. Il le plus petit des douze (12) départements du Bénin en termes de superficie.

Contrairement à tous les autres départements, celui du Littoral est constitué d'une seule commune, Cotonou, d'une superficie de 79 Km². Il est composé de 13 arrondissements et de 140 quartiers de ville. C'est la capitale économique du Bénin.

3.4.2 Infrastructures sociocommunautaires

Le Littoral demeure le département qui bénéficie de plus d'infrastructures à cause de son statut de capitale économique du Bénin.

La couverture sanitaire est la meilleure du pays. On dénombre plusieurs centres de formation de référence.

L'accès à l'eau potable semble être assuré, mais des quartiers entiers demeurent encore sans adduction d'eau.

Sur le plan de l'éducation, même si la couverture ne semble pas être réglée par l'Etat, les nombreux centres de formation privés comblent le vide. On dénombre 235 écoles primaires publiques, 18 collèges à 1er cycle et 24 collèges à 2ème cycle.

3.5 Description et caractéristique du département du Mono

3.5.1 Présentation du département

Situé au sud-ouest de la République du Bénin, le département du Mono couvre une superficie de 1605 km². Six communes composent ce département. Il s'agit de: Athiémé, Bopa, Comè, Grand-Popo, Houéyogbé et Lokossa. On y dénombre 276 villages regroupés en 35 Arrondissements..

3.5.2 Infrastructures sociocommunautaires

Les infrastructures sociocommunautaires sont insuffisantes et n'arrivent pas à satisfaire le besoin de la population dont l'effectif est en pleine croissance.

Sur le plan sanitaire, on dénombre pour une population de 360 037 personnes 74 formations sanitaires se décomposant comme suit : 37 maternités, 21 Centres de Santé, 31dispensaires

La plupart des populations de ce département ont accès à l'eau potable mais des efforts importants restent à faire.

Sur le plan scolaire, l'ensemble du département du Mono dispose de 237 écoles primaires, 23 collèges à 1er cycle et 10 collèges à 2ème cycle.

3.6 Description et caractéristique du département du Couffo

3.6.1 Présentation du département

Situé au sud-ouest de la République du Bénin, le département du Couffo couvre une superficie de 2404 km² et regroupe les six communes d'Aplahoué, de Djakotomey, de Dogbo de Klouékanmè, de Lalo, et de Toviklin, qui totalisent 367 villages.

3.6.2 Infrastructures sociocommunautaires

Pour une population totale de 524 586 personnes réparties dans 367 villages.

Dans le domaine sanitaire, on dénombre : 14 dispensaires, 42 Centres de Santé, 12 maternités.

Sur le plan de l'éducation on dénombre 269 écoles primaires, 18 collèges à 1er cycle et 5 à 2ème cycle.

3.7 Description et caractéristique du département du Zou

3.7.1 Présentation du département

Le département du Zou partage avec le département des Collines la partie centrale de la République du Bénin. Il comprend les neuf (9) communes

d'Abomey, d'Agbangnizoun, de Bohicon, de Covè, de Djidja, de Ouinhi, de Zangnanado, de Za-kpota et de Zogbodomey.

S'étendant sur une superficie de 5 243 km2, le nouveau département du Zou occupe la zone Sud de l'ancien département du Zou, compte 423 villages regroupés en soixante seize (76) Arrondissements.

3.7.2 Infrastructures sociocommunautaires

D'énormes difficultés existent en matière d'approvisionnement en eau potable. En effet, sur un effectif de 423 villages, moins d'un tiers (126 villages) disposent d'eau et forages.

Dans le domaine de l'éducation on dénombre 333 écoles primaires, 16 collèges à 1er cycle et 8 collèges à 2ème cycle. Dans le domaine de la santé, on dénombre : 10 maternités, 56 Centres de Santé, 28 dispensaires, 01 hôpital départemental.

3.8 Description et caractéristique du département des collines

3.8.1 Présentation du département

Le département des Collines est limité au sud par le département du Zou, au nord-ouest par la Donga et au nord-est par le Borgou. Il couvre une superficie de 13 931 km² et est constitué de six communes que sont Bantè, Dassa-Zoumè, Glazoué, Ouèssè, Savalou et Savè, toutes traversées par des collines, d'où son nom symbolique de département des Collines. On y dénombre un total de 297 villages et quartiers de ville répartis dans 60 arrondissements.

3.8.2 Infrastructures sociocommunautaires

Le département des Collines dispose du point de vue des infrastructures sanitaires, de 38 maternités, 4 Centres de Santé de la Commune, 33 Centres de Santé d'Arrondissement et 82 dispensaires pour les 297 villages.

L'approvisionnement en eau de boisson pose encore beaucoup de problèmes.

On dénombre sur le plan de l'éducation, 145 écoles primaires publiques, 21 collèges à 1er cycle et 5 à 2ème cycle.

3.9 Description et caractéristique du département de l'Alibori

3.9.1 Présentation du département

Le département de l'Alibori couvre les communes de Malanville, Karimama, Sègbana, Gogounou, Banikoara et Kandi, soit six communes regroupant au total 229 villages Il a une superficie de 26 242 km² (23% du territoire national).

3.9.2 Infrastructures sociocommunautaires

La couverture sanitaire des villages en termes de présence d'infrastructures sanitaires est mal assurée. En effet, on dénombre : 26 dispensaires, 23 maternités, 27 centres de Santé.

L'accès à l'eau potable constitue un véritable problème dans ce département où c'est à peine que chaque arrondissement du département dispose d'adduction d'eau et forages publics.

Sur le plan de l'éducation, le département dispose de 215 écoles primaires et 15 collèges à 1er cycle c'est-à-dire de la 6ème en 3ème et 3 collèges à 2 cycles (de la 6ème en Terminale).

3.10 Description et caractéristique du département du Borgou

3.10.1 Présentation du département

Le département du Borgou s'étend sur huit (8) communes. Il s'agit des communes de Kalalè, N'dali, Pèrèrè, Nikki, Sinendé, Bembèrèkè, Tchaourou et de Parakou. Le Borgou couvre une superficie de 25 856 km² (23% du territoire national) dont 13 962 km² de terres cultivables (54% de la superficie totale du département). On dénombre 310 villages.

3.10.2Infrastructures sociocommunautaires.

Au plan sanitaire, on dénombre pour une population de 724 171 personnes répartie dans 310 villages, 46 Centres de Santé, 45 dispensaires.
Sur le plan scolaire, dans ce département, on dénombre 401 écoles primaires publiques pour 310 villages, 28 collèges à 1er cycle et 10 à 2ème cycle. Le département abrite l'Université de Parakou.

3.11 Description et caractéristique du département du Plateau

3.11.1Présentation du département

Le département du Plateau est situé dans la partie méridionale du Bénin. Il comprend cinq (5) communes (Kétou, Pobè, Adja-Ouèrè, Sakété et Ifangni), deux cent dix huit (218) villages regroupés en vingt (29) Arrondissements. Ce département couvre une superficie de 3 264km², soit environ 3% de la superficie nationale.

3.11.2Infrastructures sociocommunautaires

Le département du Plateau est faiblement couvert en adduction d'eau. Au total 217 écoles existent pour couvrir les besoins en infrastructures scolaires pour les 218 villages. On note pour l'ensemble du département 15 collèges à 1 cycle et un seul à 2 cycles, ce qui est loin de satisfaire aujourd'hui ses besoins réels. Sur le plan sanitaire, on dénombre : 05 maternités, 30 Centres de Santé, 08 dispensaires.

3.12 Description et caractéristique du département de l'Ouémé

3.12.1Présentation du département

Le département de l'Ouémé est situé au Sud-Est du Bénin. Avec 405 villages et une superficie de 1 281 km², le département de l'Ouémé est constitué des neuf (9) communes suivantes: d'Adjara, d'Akpro-Missérété, d'Avrankou, d'Adjohoun, de Bonou, de Dangbo, de Sèmè- Kpodji, des Aguégués et de Porto-

novo la capitale administrative du Bénin. Le département compte 52 Arrondissements.

3.12.2Infrastructures sociocommunautaires.

Les infrastructures sociocommunautaires dans le département sont encore loin de satisfaire la population de ce département dont l'effectif est en pleine croissance.

Sur le plan sanitaire, on dénombre pour une population de 730 772 personnes, 27 maternités, 08 Centres de Santé de Commune ,19 Centres de Santé, 22 dispensaires, 01 hôpital départemental.

L'accès à l'eau potable semble poser encore plus de problèmes. Aucun arrondissement du département n'a été entièrement couvert en matière d'adduction et de forages publics. Ainsi sur 405 villages, 162 seulement disposent d'adduction d'eau.

Sur le plan scolaire, en dehors du Littoral c'est le département qui dispose de plus d'infrastructures éducatives : 428 écoles primaires pour 405 villages, 44 collèges à 1er cycle et 21 à 2ème cycle.

METHODE DE L'ETUDE

4 METHODE

4.1 Type d'étude

Il s'est agit d'une étude transversale à visée descriptive et analytique.

4.2 Population d'étude

La population cible s'est composée des adultes des douze départements du Bénin.

Sont inclus dans l'enquête, les sujets âgés de 25 ans au moins et de 64 ans au plus, le jour de l'enquête et résidant au Bénin depuis 6 mois au moins.

Sont exclus de l'étude :

- Les sujets n'ayant pas donné leur consentement pour participer à l'enquête et/ ou ceux à qui on a rendu 02 visites infructueuses.
- Les personnes incapables de répondre aux questions
- Les femmes enceintes.

4.3 Echantillonnage

4.3.1 Taille échantillon

Le calcul de la taille de l'échantillon est effectué selon la formule de Schwartz [6]: n = kZα2 p.q. / i2

n = taille de l'échantillon

p = 27,8% (prévalence de l'HTA dans le département du littoral du Bénin : [5])

q = (1- p) = (1-0,278) = 0 ,722

α = 5 % d'où Zα = 1,96

k = effet grappe = 2

i = 1,5 % (précision désirée)

$$n = \frac{2x1,96^2 x0,278 x0,722}{(0,015)^2} = 6853$$

La taille obtenue est de : 6853

4.3.2 Technique d'échantillonnage

L'étude a été menée selon une technique de sondage en grappes telle que proposée par l'OMS pour l'enquête STEPS de dépistage des facteurs de risque des MNT. La base de sondage a été constituée par la liste des 546 arrondissements du Bénin [Voir Annexe 4]. Une grappe correspondait à un arrondissement.

- Choix des arrondissements

Pour choisir les arrondissements, nous avons inscrit sur une liste les douze départements dans un ordre tiré au hasard. Nous avons ensuite fait la liste de tous les arrondissements avec leur population respective de même que leurs populations cumulées [Voir Annexe 4]. Nous avons choisi de prendre 60 grappes, soit 60 arrondissements. La taille de chaque grappe était alors de 115 individus (6853/60). Le détail correspondant au choix des grappes figure en annexe I.

- Choix des quartiers et villages

Dans chaque arrondissement (grappe) sélectionné, nous avons fait la liste des quartiers et villages avec leurs populations respectives. La moitié des quartiers ou villages de chaque arrondissement retenu a été tirée au hasard.

- Choix des individus

L'enquêteur s'est placé au centre du quartier ou village et a tiré au hasard une direction. Dans cette direction, il a choisi de rentrer dans une concession sur deux. Dans chaque concession, les sujets à enquêter ont été identifiés selon la méthode de Kish. Pour ce faire, l'enquêteur a numéroté au sein de chaque concession tous les ménages et en a choisi au hasard la moitié. Par ménage sélectionné, il a recensé tous les individus par ordre décroissant d'âge, en commençant par les hommes. Il s'est ensuite référé à la table KISH des ménages (Voir annexe 1) pour identifier le Tableau Kish correspondant au numéro du ménage. La synthèse Kish des huit tableaux lui a alors permis d'identifier l'individu à enquêter à partir de son numéro.

L’étude a été menée selon une technique de sondage en grappes. La base de sondage est constituée des 546 arrondissements du Bénin.

Une liste des 12 départements du Bénin a été établie après un choix aléatoire des départements.

Une liste des arrondissements de ces départements ainsi que leurs populations cumulées a aussi été établie.

Nous avons choisi de prendre 60 grappes. Une grappe correspond à un arrondissement.

Un pas de grappe a été défini. (Population cumulée des adultes sur le nombre de grappes = 139 416).

Nous avons choisi un nombre au hasard entre 1 et le pas de grappe. C’est 312. Celui – ci nous a permis de choisir la 1ère grappe sur la liste de la population cumulée. A celle-ci nous ajouterons le pas de grappe pour déterminer la 2ème grappe, et il en sera ainsi jusqu’à choisir les 60 grappes ou arrondissements.

La taille de chaque grappe est de : 115 individus (6853/60).

Nous avons choisi d’enquêter la moitié des quartiers et villages des arrondissements sélectionnés, faute de moyens matériels et financiers.

Les 115 individus à enquêter ont été choisis dans ces quartiers et villages selon la méthode de Kish recommandée par l’OMS pour l’enquête STEPS.

Ces informations (population) ont été obtenues à partir des données du troisième recensement général de la population et de l’habitat au Bénin en 2002 [7].

(Méthode Kish : voir en annexe1)

4.4 Collecte des données

4.4.1 L’outil de collecte

Il est constitué par la partie de l’instrument STEPS de l’OMS relative aux informations démographiques et à la consommation de tabac et d’alcool. Cet

outil est un guide d'entretien qui couvre trois niveaux ou *Steps* différents dans l'évaluation des facteurs de risque (voir Annexe 2).

Le *step* 1 se rapporte aux mesures comportementales dont la consommation de tabac et d'alcool.

Le *step* 2 se rapporte aux mesures physiques.

Le *step* 3 aux mesures biochimiques.

4.4.2 Le matériel

- Des tables de conversion permettant de convertir des volumes de boissons alcoolisées en nombre de verre standard [Annexe 3].
- Des bols gradués
- Tensiomètre électronique OMRON
- Toise métallique
- Mètre ruban
- Pèse-personne HANA

4.4.3 Equipe de collecte

Elle était composée de 40 enquêteurs repartis en 2 groupes de 10 binômes. Chaque groupe a été encadré par 2 superviseurs.

Aux binômes était affecté au besoin un traducteur (un relais communautaire du quartier).

4.4.4 Durée de la collecte

La collecte des données a duré 6 semaines.

4.4.5 Déroulement de l'enquête

Les enquêteurs ont été formés pendant 48 heures sur la technique d'échantillonnage et l'outil de collecte. Un pré-test des outils a ensuite été fait dans un des quartiers non retenus (Xwlacodji) pendant 24 heures le jour suivant.

L'enquête s'est déroulée simultanément dans quatre départements pendant 15 jours. Elle a commencé par les départements du Sud et s'est achevée par ceux

du Nord: Ouémé –Plateau et Atlantique-Littoral (01-13 juillet 2008) ; Mono-Couffo et Zou- Collines (21juill- 03aout) puis Borgou- Alibori et Atacora-Donga (11-24août).

En fin de journée, les superviseurs de la collecte des données faisaient le point avec le chef d'équipe. Ils ont veillé à l'exhaustivité des données, au bon remplissage du questionnaire et la complétude des données manquantes.

4.4.6 Technique de collecte

La technique utilisée a été celle d'une entrevue individuelle structurée avec la personne à enquêter.

4.4.7 Difficultés rencontrées

- Certains villages étaient difficiles d'accès de par leur situation géographique et la disposition des maisons dans d'autres avait rendu difficile l'application de notre technique d'échantillonnage.
- La réticence de certaines personnes et l'indisponibilité d'autres nous a obligé à nous conformer à leur emploi du temps.
- La barrière linguistique lors de l'entretien nous a contraints à utiliser des traducteurs.

4.4.8 Considérations éthiques et consentement éclairé

Conformément au principe du secret professionnel la confidentialité a été rigoureusement respectée lors de l'enquête. Le consentement verbal ou écrit du patient a été obtenu avant l'administration du guide.

4.5 Analyse des données et test statistiques

Une double saisie des données a été faite dans le logiciel Epi data. L'analyse des données a été faite selon les recommandations du STEP en utilisant le logiciel Epi info version 3.3.2.

Des tableaux et figures ont été réalisés pour voir la distribution du phénomène dans la population de référence. Le test de Khi-2 a été utilisé pour

comparer les fréquences et le test de Student pour comparer les moyennes. La différence était statistiquement significative pour un p égale à 0,05.

RESULTATS

5 RESULTATS

5.1 Description de la population d'étude

Les 6904 sujets inclus dans l'étude étaient répartis en 3485 sujets de sexe masculin et 3419 de sexe féminin, soit un sex-ratio de 1,02. La moyenne d'âge était de 42,7 ans ± 12,4 ans. Plus d'un sujet sur deux n'avait aucune instruction officielle et ils étaient majoritairement d'ethnie Fon (40,1%). Près de deux sujets sur trois appartenaient au milieu rural.

Le tableau I résume la répartition des sujets de l'étude en fonction de l'âge, du sexe, du milieu socioculturel et du milieu d'appartenance et le tableau II les informations relatives à leur niveau d'instruction, à leur activité professionnelle et à leur département.

Tableau I: Caractéristiques sociodémographiques des sujets enquêtés au Bénin en 2008.

Variables	Fréquence (n)	Pourcentage (%)
**Age**		
25-34	2167	31,4
35-44	1677	24,3
45-54	1179	17,1
55-64	**1881**	**27,2**
**Sexe**		
Masculin	**3485**	**50,5**
Féminin	3419	49,5
**Milieu socioculturel**		
Adja	1050	15,2
Fon	**2769**	**40,1**
Bariba	904	13,1
Dendi	335	4,8
Yom	250	3,6
Peuhl	275	4
Otamari	243	3,5
yoruba	978	14,3
Autres	100	1,4
**Milieu**		
Urbain	2359	34,2
Rural	**4545**	**65,8**

Tableau II: Répartition des sujets enquêtés en fonction de leur niveau d'instruction, activité professionnelle et département au Bénin en 2008.

Variables	Fréquence (n)	Pourcentage (%)
**Niveau d'instruction**		
Aucune	**4290**	**62,1**
Primaire	1761	25,5
Secondaire	679	9,9
Université	174	2,5
**Activité professionnelle**		
Indépendant	**5410**	**78,4**
Maître (esse) de maison	646	9,3
Employé dans le privé	269	3,9
Employé de l'état	225	3,3
Chômeur	137	2
Retraité (e)	115	1,6
Etudiant (e)	102	1,5
**Départements**		
Plateau	467	6,8
Atlantique	**914**	**13,2**
Alibori	464	6,7
Zou	687	9,9
Littoral	584	8,5
Mono	345	5
Ouémé	693	10,0
Couffo	573	8,3
Collines	571	8,3
Borgou	687	10,0
Atacora	574	8,3
Donga	345	5

STEP 1

5.2 Prévalence du tabagisme

Parmi les 6904 sujets de l'étude, 1108 consommaient du tabac, soit une prévalence de 16,0% [IC95%: 15,2% – 16,9%].

Parmi les 3485 sujets de sexe masculin, 877 en consommaient, soit une prévalence de 25,2%. Dans le groupe des 3419 sujets de sexe féminin, 231 en consommaient, soit une prévalence de 6,7%. La prévalence du tabagisme était significativement plus élevée chez les hommes ($p < 10^{-7}$).

Le tableau III montre la prévalence du tabagisme en fonction de l'âge. Cette prévalence augmentait significativement avec l'âge ($p < 10^{-8}$).

Tableau III : Répartition des consommateurs de tabac par tranche d'âge au Bénin en 2008.

**Tranches d'âge (ans)**	**Consommateurs**		**Non consommateurs**		**Total**
	**n**	**%**	**n**	**%**	
25-34	252	11,6	1915	88,4	2167
35-44	254	15,1	1423	84,9	1677
45-54	201	17,0	978	83	1179
55-64	401	**21,3**	1480	78,7	1881
**25-64**	1108	16,0	5796	84	6904

5.2.1 Prévalence de la consommation quotidienne de tabac

Parmi les 6904 sujets, 989 consommaient quotidiennement du tabac, soit une prévalence de 14,3% [IC95%: 13,5% - 15,2%].

Parmi les 3485 sujets de sexe masculin, 785 en consommaient quotidiennement, soit une prévalence de 22,5%. Dans le groupe des 3419 sujets de sexe féminin, 204 en consommaient quotidiennement, soit une prévalence de 5,9%. La prévalence de la consommation quotidienne de tabac était significativement plus importante chez les hommes ($p < 10^{-7}$).

Le tableau IV montre la répartition des consommateurs de tabac en fonction de leur fréquence de consommation.

Tableau IV: Répartition des consommateurs de tabac en fonction de leur fréquence de consommation et de leur sexe au Bénin en 2008.

Sexe	Quotidienne		Non quotidienne		Total
	n	%	n	%	
M	785	**89,5**	92	10,5	877
F	204	88,3	27	11,7	231
**Total**	989	89,2	119	10,8	1108

M=Masculin F= Féminin

5.2.2 Prévalence de la consommation de tabac fumé

Parmi les 6904 sujets, 599 consommaient du tabac fumé, soit une prévalence de 8,7% [IC95%: 8,0% - 9,4%].

Parmi les 3485 sujets de sexe masculin, 542 fumaient, soit une prévalence de 15,5%. Dans le groupe des 3419 femmes, 57 fumaient soit une prévalence de 1,7%. La prévalence de la consommation de tabac fumé était significativement plus élevée chez les hommes ($p < 10^{-7}$).

Le tableau V montre la prévalence de la consommation de tabac fumé en fonction de l'âge. Cette prévalence paraissait plus élevée chez les sujets de 45 à 54 ans, mais la différence n'était pas significative (p = 0,09).

Tableau V: Répartition des consommateurs de tabac fumé par tranche d'âge au Bénin en 2008.

Tranches d'âge (ans)	Fumeurs		Non fumeurs		Total
	n	%	n	%	
25-34	168	7,8	1999	92,2	2167
35-44	151	9,0	1526	91	1677
45-54	121	**10,3**	1058	89,7	1179
55-64	159	8,4	1722	91,6	1881
**25-64**	599	8,7	6305	91,3	6904

Parmi les 6904 sujets, 522 fumaient quotidiennement, soit une prévalence de 7,6% [IC95%: 6,9% - 8,2%].

Parmi les 3485 sujets de sexe masculin, 480 fumaient quotidiennement, soit une prévalence de 13,8%. Dans le groupe des 3419 femmes, 42 fumaient

quotidiennement, soit une prévalence de 1,2%. La prévalence de la consommation quotidienne de tabac fumé était significativement plus importante chez les hommes que chez les femmes ($p < 10^{-7}$).

La plupart de ces sujets ont commencé par fumer quotidiennement à l'âge de 20 ans (20 ans chez les hommes et 18 ans chez les femmes). L'âge minimal de début était de 10ans.

Presque tous les fumeurs quotidiens utilisaient la cigarette industrielle. En effet, parmi les 522 fumeurs quotidiens, 421 utilisaient la cigarette industrielle, soit une proportion de 80,6%.

Le tableau VI montre le nombre moyen de cigarettes fumé chaque jour. Ce nombre était significativement plus élevé chez les hommes ($p < 10^{-4}$).

Tableau VI : Nombre moyen de cigarettes fumées par jour au Bénin en 2008.

**Sexe**	**Nombre moyen**
Masculin	**5,4**
Féminin	1,5
**Total**	5,2

5.2.3 Prévalence de la consommation de tabac non fumé

Parmi les 6904 sujets, 636 consommaient du tabac non fumé, soit une prévalence de 9,2% [IC95%: 8,5% -9,9%].

Parmi les 3485 sujets de sexe masculin, 442 en consommaient, soit une prévalence de 12,7%. Dans le groupe des 3419 femmes, 194 en consommaient, soit une prévalence de 5,7%. La prévalence de la consommation de tabac non fumé était significativement plus élevée chez les hommes ($p <10^{-7}$).

Le tableau VII montre la prévalence de la consommation de tabac non fumé en fonction de l'âge. Cette prévalence augmentait significativement avec l'âge ($p < 10^{-8}$).

Tableau VII: Répartition des consommateurs de tabac non fumé par tranche d'âge au Bénin en 2008.

**Tranches d'âge (ans)**	**Consommateurs**		**Non consommateurs**		**Total**
	**n**	**%**	**n**	**%**	
25-34	107	4,9	2060	95,1	2167
35-44	126	7,5	1551	92,5	1677
45-54	113	9,6	1066	90,4	1179
55-64	290	**15,4**	1591	84,6	1881
**25-64**	636	9,2	6268	90,8	6904

Parmi les 6904 sujets, 577 consommaient quotidiennement du tabac non fumé, soit une prévalence de 8,3% [IC95%: 7,7% -9,0%].

Parmi les 3485 sujets de sexe masculin, 396 en consommaient quotidiennement, soit une prévalence de 11,4%. Dans le groupe des 3419 femmes, 181 en consommaient quotidiennement, soit une prévalence de 5,3%.

La consommation quotidienne de tabac non fumé était significativement plus élevée chez les hommes ($p < 10^{-8}$).

La prévalence de la consommation du tabac diminuait significativement avec le niveau d'instruction ($p < 10^{-8}$). Elle était significativement plus faible en milieu urbain qu'en milieu rural ($p < 10^{-7}$). La plus grande prévalence a été enregistrée dans l'Atacora. Le groupe socioculturel le plus touché était les Otamari (42%) et le moins touché les fon (7%). Les tableaux VIII et IX montrent la prévalence de la consommation de tabac en fonction des caractéristiques sociodémographiques des sujets.

5.2.4 Prévalence du tabagisme et facteurs socioculturels

Tableau VIII: Répartition des consommateurs de tabac en fonction du niveau d'instruction, de l'appartenance socioculturelle et du milieu de provenance au Bénin en 2008.

**Variables**	**Consommateurs de tabac**		**p**
	**n**	**%**	
**Niveau d'instruction**			
Aucun	813	**18,9**	< 10-8
Primaire	239	13,6	
Secondaire	49	7,2	
Universitaire	7	4,0	
**Milieu socioculturel**			
Adja	148	14,1	
Fon	193	7	
Bariba	277	30,6	
Dendi	53	15,8	
Yom	102	40,8	< 10-8
Peulh	66	24	
Otamari	102	**42**	
Yoruba	145	14,8	
Autres	22	22	
**Milieu de provenance**			
Rural	886	**19,5**	
Urbain	222	9,4	< 10-7

Tableau IX: Répartition des consommateurs de tabac par département au Benin en 2008.

Départements	Tabac fumé		Tabac non fumé	
	**n**	**%**	**n**	**%**
Plateau	45	9,6	32	6,8
Atlantique	60	6,5	6	0,6
Alibori	38	8,2	70	15,1
Zou	59	8,6	24	3,5
Littoral	17	2,9	6	1,0
Mono	35	10,1	25	7,2
Ouémé	25	3,6	6	0,9
Couffo	59	10,3	55	9,6
Collines	52	9,1	27	4,7
Borgou	74	10,8	147	21,4
Atacora	87	**15,1**	156	**27,1**
Donga	48	13,9	82	23,8

5.3 Prévalence de la consommation d'alcool

5.3.1 Consommation d'alcool au cours des douze derniers mois

Parmi les 6904 sujets de l'étude, 4097 avaient consommé au moins une fois une boisson alcoolisée au cours des douze derniers mois qui ont précédé l'étude, soit une prévalence de 59,3% [IC95%: 58,2% -60,5%].

Parmi les 3485 sujets de sexe masculin, 2394 en avaient consommé, soit une prévalence de 68,7%. Dans le groupe des 3419 sujets de sexe féminin, 1703 en avaient consommé, soit une prévalence de 49,8%. La prévalence de la consommation d'alcool était significativement plus élevée chez les hommes ($p < 10^{-7}$).

Par ailleurs, Le nombre moyen de verres consommé par jour était de 3,9 verres ± 3,2 verres chez les hommes versus 2,6 verres ± 1,8 verre chez les femmes ($p < 10^{-6}$). Le plus grand nombre de verre consommé par jour était de 40 chez les hommes versus 20 chez les femmes.

Le tableau X montre la prévalence de la consommation d'alcool par tranches d'âge. Cette prévalence était significativement plus élevée chez les sujets de 45 à 54 ans ($p < 10^{-5}$).

Tableau X : Répartition des sujets selon leur consommation d'alcool au cours des douze derniers mois en fonction de l'âge au Bénin en 2008.

**Tranches d'âge (ans)**	**Consommateurs**		**Non consommateurs**		**Total**
	**n**	**%**	**n**	**%**	
25-34	1283	59,2	884	40,8	2167
35-44	1047	62,4	630	37,6	1677
45-54	744	**63,1**	435	36,9	1179
55-64	1023	54,4	858	45,6	1881
**25-64**	4097	59,3	2807	40,7	6904

Parmi les 6904 sujets, 4045 avaient eu une consommation ponctuelle excessive (28 jours en moyenne au cours des douze derniers mois), soit une prévalence de 58,6% [IC95%: 57,4% – 59,7%].

Parmi les 3485 sujets de sexe masculin 2364 avaient eu une consommation ponctuelle excessive soit 67,8%. Parmi les 3419 sujets de sexe féminin, 1681 avaient eu un tel comportement soit une prévalence de 49,2%.La prévalence de la consommation ponctuelle excessive d'alcool était significativement plus élevée chez les hommes que chez les femmes ($p < 10^{-7}$).

Le tableau XI donne la prévalence de la consommation quotidienne d'alcool au cours des douze derniers mois par sexe. Cette prévalence était significativement plus élevée chez les hommes. ($p < 10^{-7}$).

Tableau XI: Répartition des consommateurs quotidiens d'alcool par sexe au cours des douze derniers mois au Bénin.

**Sexe**	**Consommateurs quotidiens**		**Non consommateurs quotidiens**		**Total**
	**n**	**%**	**n**	**%**	
M	576	**16,5**	2909	83,5	3485
F	186	5,4	3233	94,6	3419
**Total**	762	11,0	6142	89	6904

Le tableau XII montre la répartition des consommateurs d'alcool en fonction de leur fréquence de consommation. Plus d'un homme sur trois consommaient de l'alcool 1 à 4 jours dans la semaine, alors que plus d'une femme sur quatre en consommaient 1 à 3 jours par mois.

Tableau XII: Répartition des consommateurs d'alcool par sexe et par fréquence de consommation au cours des douze derniers mois au Bénin.

**Fréquences**	**Hommes**		**Femmes**		**Hommes et femmes**	
	**n**	**%**	**n**	**%**	**n**	**%**
Quotidienne	576	24,1	186	10,9	762	18,6
5-6 jours/semaine	241	10,0	115	6,8	356	8,7
1-4 jours/semaine	822	**34,3**	460	27,0	1282	31,3
1-3 jours/mois	502	21	499	**29,3**	1001	24,4
< 1jour/mois	253	10,6	443	26,0	696	17
**Total**	2394	100,0	1703	100,0	4097	100,0

5.3.2 Consommation d'alcool au cours des trente derniers jours

Au cours des 30 derniers jours qui ont précédé l'étude, 3308 sujets parmi les 6904 avaient consommé de l'alcool au moins une fois, soit une prévalence de 47,9% pour la consommation actuelle [IC95%: 46,7% – 49,1%].

Parmi les 3485 sujets de sexe masculin, 2064 en avaient consommé au cours de cette période, soit une prévalence de 59,2%. Parmi les 3419 sujets de sexe féminin, 1244 en avaient consommé au cours de la même période, soit une prévalence de 36,4%. La prévalence de la consommation actuelle d'alcool était significativement plus élevée chez les hommes ($p < 10^{-7}$).

Parmi ces consommateurs actuels, 1197 avaient bu au moins 4 jours au cours de la semaine écoulée avant l'enquête, soit une proportion de 36,2% pour la consommation importante.

Parmi les 2064 consommateurs actuels de sexe masculin, 885 avaient eu une consommation importante d'alcool, soit une proportion de 42,9%. Chez les sujets de sexe féminin, cette proportion était de 25,1% (312 sujets sur 1244). La proportion des consommateurs actuels ayant eu une consommation importante au cours des sept derniers jours qui ont précédés l'enquête était significativement plus élevée chez les hommes ($p < 10^{-7}$).

Le tableau XIII montre la proportion des consommateurs actuels ayant eu une consommation importante au cours de la semaine écoulée avant l'enquête en fonction de l'âge. Cette prévalence augmentait significativement avec l'âge ($p < 10^{-6}$).

Le tableau XIII montre la répartition des consommateurs actuels par degré de risque en fonction de l'âge au cours des sept derniers jours qui ont précédé l'étude. La consommation nocive d'alcool augmentait significativement avec l'âge ($p = 0,002$).

Tableau XIV: Répartition des consommateurs actuels par degré du risque (dangereux ou nocif) en fonction de l'âge au cours des sept derniers jours, Bénin, 2008.

**Tranches d'âge (ans)**	**Consommation dangereuse**		**Consommation nocive**		**Total**
	**n**	**%**	**n**	**%**	
25-34	76	7,6	40	4	1003
34-44	91	10,6	48	5,6	855
45-54	47	7,7	41	6,7	607
55-64	98	**11,6**	74	**8,8**	843
**Total**	312	9,4	203	6,1	3308

Sur les 6904 sujets de l'étude, 203 avaient eu une consommation nocive d'alcool au cours des sept derniers jours qui ont précédé l'étude, soit une prévalence de 2,9% [IC95%: 2,6% – 3,4%].

La figure 1 montre les prévalences de la consommation d'alcool au cours des sept derniers jours par niveau de risque

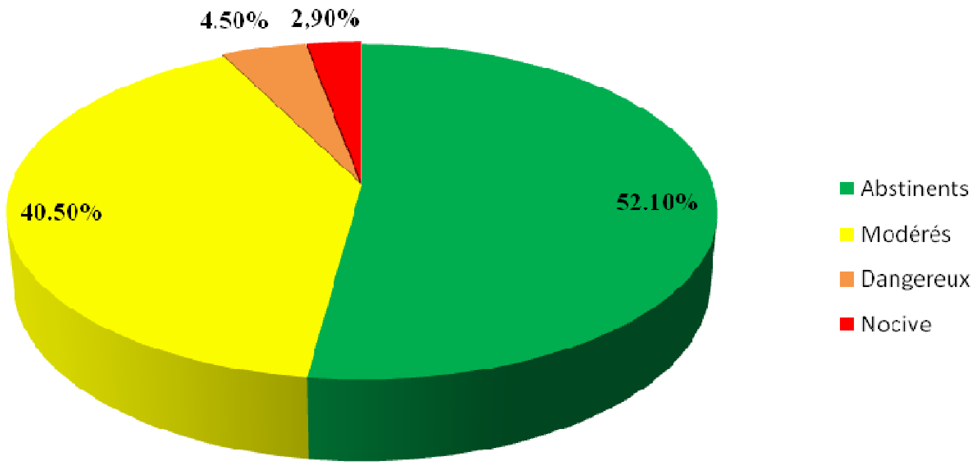

Figure 1: Prévalence de la consommation d'alcool par niveau de risque au cours des sept derniers jours.

La prévalence de la consommation à risque était de 9,3% chez les hommes (326 sur les 3485 de l’étude) et 5,5% chez les femmes (189 femmes sur les 3419 de l’étude). La prévalence de la consommation à risque chez les hommes était significativement plus élevée que chez les femmes ($p < 10^{-7}$).

La prévalence de la consommation nocive était de 4,6% chez les hommes (162 hommes sur les 3485). Chez les femmes, elle était de 1,2% (41 femmes sur les 3419). La prévalence de la consommation nocive était également significativement plus élevée chez les hommes ($p < 10^{-7}$).

La prévalence de la consommation nocive était significativement plus élevée chez les sujets ayant peu ou pas d’instruction. Elle paraissait plus élevée en milieu rural qu’en milieu urbain; mais la différence n’était pas significative.

La plus forte prévalence de consommation nocive était rencontrée en milieu Otamari (6,2%) alors que la plus faible était rencontrée en milieu peulh (0,4%). Elle était de 5,2% dans le Mono (département le plus touché) et de 1,2% dans le Littoral (département le moins touché).

Les tableaux **XV** et XV résument ces informations.

Tableau XVI: Répartition des sujets ayant une consommation nocive d'alcool en fonction du niveau d'instruction, de l'appartenance socioculturelle et du milieu de provenance au Bénin en 2008.

Variables	Consommation nocive (+)		p
	n	%	
**Niveau d'instruction**			
Aucun	131	3,1	
Primaire	65	**3,7**	**< 0,001**
Secondaire	7	1,0	
Universitaire	0	0	
**Milieu socioculturel**			
Adja	41	3,9	
Fon	65	2,3	
Bariba	33	3,6	
Dendi	7	2,1	
Yom	8	3,2	**0,002**
Peulh	1	0,4	
Otamari	15	**6,2**	
Yoruba	32	3,3	
Autres	1	1	
**Milieu de provenance**			
Rural	146	3,2	**NS**
Urbain	57	2,4	

Tableau XV: Répartition des sujets ayant une consommation nocive d'alcool par département au Bénin en 2008.

Départements	Consommation nocive (+)	
	**n**	**%**
Plateau	23	4,9
Atlantique	23	2,5
Alibori	11	2,4
Zou	16	2,3
Littoral	7	1,2
**Mono**	**18**	**5,2**
Ouémé	13	1,9
Couffo	18	3,1
Collines	17	3
Borgou	19	2,8
Atacora	28	4,9
Donga	10	2,9

Par ailleurs, la consommation nocive d'alcool était plus fréquente chez les consommateurs de tabac que chez les non consommateurs de tabac.

En effet, parmi les 1108 consommateurs de tabac de notre étude, 105 avaient eu une consommation nocive d'alcool les sept derniers jours qui ont précédé l'enquête, soit une prévalence de 9,5%, alors que parmi les 5796 sujets non consommateurs de tabac, 100 avaient eu une consommation nocive au cours de la même période, soit une prévalence de 1,7%. Le tableau XVI montre cette différence entre les consommateurs de tabac et les non consommateurs face à l'alcool.

TableauXVI: Consommation nocive d'alcool chez les consommateurs de tabac et les non consommateurs de tabac.

	Nocive (+)		Nocive (-)		Total
	**n**	**%**	**n**	**%**	
Consommateurs de tabac	105	**9,5**	1003	90,5	1108
Non consommateurs de tabac	100	1,7	5696	98,3	5796

Réciproquement, la consommation de tabac était plus fréquente chez les sujets ayant eu une consommation nocive d'alcool au cours des sept derniers jours qui ont précédé l'enquête que chez les abstinents.

En effet, parmi les 205 sujets ayant eu une consommation nocive au cours de la semaine écoulée avant l'enquête, 105 consommaient du tabac, soit une prévalence de 51,2%, alors que sur les 2807 abstinent, 246 consommaient du tabac, soit une prévalence de 8,8%.

Le tableau XVII montre cette différence entre les abstinents et les sujets ayant une consommation nocive face au tabac.

TableauXVII: consommation de tabac chez les sujets ayant une consommation nocive d'alcool et chez les abstinents.

	**Fumeurs (+)**		**Fumeurs (-)**	
	**n**	**%**	**n**	**%**
Nocive (+)	105	**51,2**	100	48,8
Abstinents	246	8,8	2561	91,2

5.4 Prévalence de l'inactivité physique

5.4.1 Prévalence de l'activité physique selon le niveau d'activité

Sur les 6904 sujets, 5485 avaient un niveau élevé d'activité physique (79,45%); 794 avaient un niveau modéré d'activité physique (11,50%) et 625 avaient un niveau bas (9,05%).

Parmi les 3485 sujets de sexe masculin, 243 ont un niveau limité d'activité physique (6,97%) et 382 sujets de sexe féminin parmi les 3419 (11,17%).

Le tableau XVIII montre la prévalence de l'activité physique selon le niveau d'activité et l'âge.

Tableau XVII : Répartition des sujets selon le niveau d'activité et l'âge au Bénin en 2008

Tranche d'âge	niveau d'activité limité		niveau d'activité moyen		niveau d'activité élevé	
	**n**	**%**	**n**	**%**	**n**	**%**
25-34	158	7,29	211	9,73	1798	82,98
35-44	96	5,72	176	10,49	1405	83,79
45-54	104	8,82	112	9,50	963	81,68
55-64	267	**14,19**	295	15,68	1319	70,13
**Total**	625	9,05	794	11,50	5485	79,45

La plus grande prévalence de l'inactivité physique était obtenue dans le département du Littoral (19,52%) et la plus faible prévalence dans le département de l'Atacora (2,09%).

Le tableau XIX montre la prévalence de l'inactivité physique selon le département.

Tableau XVIII: Répartition des sujets ayant un niveau d'activité physique limite selon le département au Bénin en 2008

Départements	n	%	Total
Plateau	46	9,85	467
Atlantique	92	10,06	914
Alibori	17	3,66	464
Zou	46	6,69	687
**Littoral**	**114**	**19,52**	**584**
Mono	34	9,85	345
Ouémé	133	19,19	693
Couffo	46	8,02	573
Collines	40	7,00	571
Borgou	33	4,80	687
Atacora	12	2,09	574
Donga	12	3,47	345
**Total**	625	9,05	6904

5.4.2 Prévalence de l'inactivité physique selon le type d'activité

5.4.2.1 Prévalence de l'inactivité physique au travail

Parmi les 6904 sujets, 1223 ne pratiquaient aucune activité physique dans le cadre de leur travail, soit une prévalence de 17,71% [IC95% :16,82%-18,64%].

Cinq cent vingt sujets sur les 3480 sujets de sexe masculin ne pratiquaient pas une activité physique dans le cadre de leur travail soit une prévalence de 14,94%. Elle était de 20,53% chez les sujets de sexe féminin (703/ 3424). La prévalence de l'inactivité physique au travail était significativement plus élevée chez les femmes ($p < 10^{-6}$).

Le tableau XX montre la prévalence de l'inactivité physique au travail selon le sexe et l'âge.

Tableau XX : Répartition des sujets selon la pratique de l'activité physique au travail, le sexe et l'âge au Bénin en 2008

**Tranche d'âge**	**Hommes**		**Femmes**	
	**AP−**	**AP +**	**AP−**	**AP +**
	**n (%)**	**n (%)**	**n (%)**	**n (%)**
25-34	167(14,64)	973(85,36)	167(16,63)	837(83,37)
35-44	116(10,17)	787(69,83)	111(14,19)	671(85,81)
45-54	64(10,68)	535(89,32)	116(19,52)	478(80,48)
55-64	173(**20,64**)	665(79,36)	309(**29,59**)	735(70,41)
**25-64**	520(14,94)	2960(85,06)	703(20,53)	2721(79,47)

AP + = pratique de l'activité physique AP − = pas d'activité physique

Sur les 2359 sujets provenant du milieu urbain, 1291 ne pratiquaient pas une activité physique au travail, soit une prévalence de 54,72% versus 49,35% chez les sujets du milieu rural. Elle était significativement plus élevée en milieu urbain ($p < 10^{-6}$).

Le tableau XXI montre la prévalence de l'inactivité physique au travail en fonction du lieu de provenance.

Tableau XIX : Répartition des sujets qui pratiquent une activité physique au travail en fonction du lieu de provenance au Bénin en 2008

**Milieu**	**Travail**		**Total**
	**n**	**%**	
urbain	1291	54,72	2359
rural	2243	**49,35**	4545

5.4.2.2 Prévalence de l'inactivité physique pour les déplacements

Pour les déplacements, 308 sujets ne pratiquaient pas d'activité physique, soit une prévalence de 4,46% [IC95% :3,99%-4,98%].

Parmi les 3480 sujets de sexe masculin, 248 ne pratiquaient pas une activité physique dans le cadre de déplacements soit une prévalence de 7,12% versus 7,41 % (254/3424) chez les sujets de sexe féminin. Elle était significativement plus élevée chez les sujets de sexe féminin ($p< 10^{-6}$). Le

tableau XXII montre prévalence de l'inactivité physique pour les déplacements selon le sexe et l'âge.

Tableau XX: Répartition des sujets selon la pratique de l'activité physique pour les déplacements, le sexe et l'âge au bénin en 2008.

Tranche d'âge	Hommes		Femmes	
	AP–	AP +	AP–	AP+
	n (%)	n (%)	n (%)	n (%)
25-34	81(7,11)	1058(92,89)	58(5,77)	946(94,23)
35-44	68(**7,53**)	835(92,47)	47(6,00)	736(94,00)
45-54	37(7,18)	561(93,82)	35(5,90)	558(94,10)
55-64	62(7,38)	777(92,62)	114(**10,93**)	929(89,07)
**Total**	248(7,12)	3231(92,88)	254(7,41)	3170(92,59)

Sur les 2359 sujets provenant du milieu urbain, 188 ne pratiquaient pas une activité physique pour leurs déplacements soit une prévalence de 07,96% versus 04,79% chez les sujets du milieu rural. Cette prévalence était significativement plus élevée en milieu urbain qu'en milieu rural ($p < 10^{-6}$).

Le tableau XXIII montre la prévalence de l'inactivité physique pour les déplacements en fonction du lieu de provenance.

Tableau: XXIII Répartition des sujets inactifs aux déplacements selon le lieu de provenance au Bénin en 2008

Milieu	Travail		Total
	n	%	
urbain	188	**7,96**	2359
rural	218	4,79	4545

5.4.2.3 Prévalence de l'inactivité physique au loisir

Au loisir, 5612 sujets ne pratiquaient aucune activité physique, soit 81,28% [IC95% :80,34%-82,19%].

Chez les 3480 sujets de sexe masculin, 2652 ne pratiquaient pas une activité physique au loisir soit 76,20% et 2960 sujets de sexe féminin ne pratiquaient aucune activité physique soit 86,44%. La prévalence de

l'inactivité physique aux loisirs est significativement plus élevée chez les femmes que chez les hommes ($p < 10^{-7}$).

Le tableau XXV montre la prévalence de l'inactivité physique aux loisirs selon le sexe et l'âge.

Tableau XXI: Répartition des sujets selon la pratique ou non de l'activité physique pour les loisirs, le sexe et l'âge au Bénin en 2008

**Tranche d'âge**	**Hommes**		**Femmes**	
	**AP–**	**AP+**	**AP–**	**AP+**
	**n (%)**	**n (%)**	**n (%)**	**n (%)**
25-34	749(65,70)	391(34,29)	828(82,47)	176(17,52)
35-44	691(76,52)	212(23,47)	660(84,39)	122(15,60)
45-54	476(79,59)	122(20,40)	513(86,50)	80(13,49)
55-64	736(**87,72**)	103(12,27)	959(**91,77**)	86(8,22)
**25-64**	2652(76,20)	828(23,79)	2960(86,44)	464(13,55)

Sur les 2359 sujets provenant du milieu urbain, 389 ne pratiquaient pas une activité physique dans le cadre de leur loisir, soit une prévalence de 16,49% versus 14,30% chez les sujets du milieu rural ($p < 10^{-6}$). La différence de prévalence était significative.

Le tableau XXV montre la prévalence de l'inactivité physique au loisir en fonction de la provenance des sujets.

Tableau XXV: Répartition des sujets qui pratiquent une activité physique au travail en fonction du lieu de provenance au Bénin en 2008

**Milieu**	**Travail**		**Total**
	**n**	**%**	
urbain	389	16,49	2359
rural	650	**14,30**	4545

5.4.3 Nombre moyen de minutes consacrées chaque jour à une activité physique

Globalement les sujets de l'étude consacraient en moyenne 208,26 minutes à une activité physique dans le cadre de leur travail. Les sujets de

sexe masculin y consacraient en moyenne 296,78 minutes versus 219,62 minutes chez les sujets de sexe féminin ($p<10^{-6}$). Cette différence était significative.

Pour leur déplacement, les sujets de l'étude consacraient en moyenne 112,61 minutes à une activité physique. Les sujets de sexe masculin y consacraient en moyenne 133,11 minutes versus 129,35 minutes chez les sujets de sexe féminin ($p<10^{-6}$). Cette différence était significative.

Dans le cadre des loisirs, les sujets de l'étude consacraient en moyenne 5,90 minutes à une activité physique. Les sujets de sexe masculin y consacraient en moyenne 13,27 minutes versus 7,35 minutes chez les sujets de sexe féminin ($p<10^{-6}$). Cette différence était significative.

Le temps moyen consacré à une activité physique au travail, pour les déplacements, au loisir diminue globalement et significativement avec l'âge ($p < 10^{-6}$).

Le tableau XXVI montre le temps moyen en minutes consacré à chaque type d'activité physique selon l'âge.

Tableau XXII : Temps moyen en minutes consacre a chaque type d'activité physique selon l'âge au bénin en 2008

Tranche d'âge	n	Travail	Déplacement	Loisir
25-34	2096	282,67	147,78	**14,93**
35-44	1637	276,41	134,97	11,38
45-54	**1143**	268,63	125,47	7,52
55-64	1833	**208,26**	**112,61**	5,90
**25-64**	6709	258,57	131,24	10,33

5.4.4 Nombre moyen de minutes consacrées chaque jour à des comportements sédentaires

Les 6904 sujets de l'étude consacraient en moyenne 413 minutes par jour à des comportements sédentaires. Les femmes y consacraient en moyenne 420,71 minutes versus 405,93 minutes chez les hommes. Ce temps variait significativement selon le sexe ($p< 10^{-4}$). Le temps moyen consacré

aux comportements sédentaires augmentait significativement avec l'âge jusqu'à 55 à 64 ans ($p < 0,05$).

Le tableau XXVII montre le temps moyen en minutes consacré par les sujets à des comportements sédentaires selon le sexe et l'âge.

Tableau XXIII : Temps moyen consacré chaque jour à des comportements sédentaires selon le sexe et l'âge au Bénin en 2008

**Tranche d'âge**	**hommes**		**femmes**		**Total**	
	**n**	**T**	**n**	**T**	**n**	**T**
25-34	1126	381,56	1006	379,94	2142	380,80
35-44	894	394,90	763	403,00	1657	398,63
45-54	586	399,09	576	421,60	1162	410,25
55-64	821	**456,53**	1037	**472,79**	1858	**465,60**
**25-64**	3427	405,93	3382	420,71	6819	413,26

5.5 Prévalence de la consommation insuffisante de fruits et légumes

Parmi les 6904 sujets de l'étude, 5389 consommaient moins de 5 portions de fruits et légumes par jour, soit une prévalence de 78,05% [IC95% :77,05%-79,02%]. Parmi les 3485 sujets de sexe masculin, 2646 consommaient moins de 5 portions de fruits et légumes, soit une prévalence de 75,92% versus 80,22% chez les 3419 sujets de sexe féminin ($p< 10^{-5}$). Cette différence était significative. Le tableau XXVIII montre la prévalence de la consommation insuffisante de fruits et de légumes en fonction de l'âge.

Tableau XXVIII : Répartition des sujets selon la consommation insuffisante de fruits et de légumes et l'âge au Bénin en 2008

**Tranche d'âge**	**< 5 portions**		**≥5 portions**		**Total**
	**n**	**%**	**n**	**%**	
25-34	1689	77,95	478	22,05	2167
35-44	1266	75,49	410	24,51	1677
45-54	923	78,28	266	21,72	1179
55-64	1511	**80,32**	361	19,68	1881
**Total**	5389	78,05	1515	21,95	6904

Prévalence de la consommation insuffisante de fruits et de légumes selon le département

Sur le plan national, la plus grande prévalence de consommation journalière insuffisante de fruits et légumes était observée dans le département du Mono (87,24%) et la plus faible prévalence était observée dans le département du Plateau.

Le tableau XXIX montre la prévalence de la consommation insuffisante de fruits et de légumes selon le département.

Tableau XXIV : Répartition des sujets selon la consommation de fruits et légumes et le département au Bénin en 2008

**Départements**	**< 5 portions**		**≥ 5 portions**		**Total**
	**n**	**%**	**n**	**%**	
Plateau	319	68,30	157	33,41	467
Atlantique	640	70,02	253	27,68	914
Alibori	401	86,42	67	14,43	464
Zou	497	72,34	174	25,32	687
Littoral	476	81,50	99	16,95	584
**Mono**	**301**	**87,24**	**53**	**15,36**	**345**
Ouémé	577	83,26	121	17,46	693
Couffo	457	79,75	118	20,59	573
Collines	457	80,03	109	19,08	571
Borgou	514	74,81	178	25,90	687
Atacora	453	78,91	128	22,29	574
Donga	297	86,08	58	16,81	345

Prévalence de la consommation insuffisante de fruits et de légumes selon le lieu de provenance

Sur les 2359 sujets du milieu urbain, 2008 consommaient moins de 5 portions de fruits et de légumes, soit 85,12% versus 3381(74,38%) chez les sujets du milieu rural. La prévalence était significativement plus élevée chez les sujets du milieu urbain ($p< 10^{-7}$).

Le tableau XXX montre la prévalence de la consommation insuffisante de fruits et de légumes selon le lieu de provenance.

Tableau XXX: Répartition des sujets selon la consommation de fruits et légumes et le lieu de provenance au Bénin en 2008

**Milieu**	**< 5 portions**		**≥5 portions**		**Total**
	**N**	**%**	**N**	**%**	
**urbain**	2008	**85,13**	351	14,87	2359
rural	3381	74,39	1164	25,61	4545
**Total**	5389	78,05	1515	21,95	6904

Prévalence de la consommation insuffisante de fruits et légumes selon le niveau d'instruction

La plus grande prévalence (87,35%) était retrouvée chez les sujets ayant un niveau d'étude universitaire. La plus faible prévalence (77,52%) était retrouvée chez les sujets n'ayant aucune instruction officielle. Cette prévalence variait significativement avec le niveau d'instruction ($p < 0,05$).

Le tableau XXXI montre la prévalence de la consommation insuffisante de fruits et de légumes selon le niveau d'instruction.

Tableau XXV : Répartition des sujets selon la consommation insuffisante de fruits et légumes et le niveau d'instruction au Bénin en 2008

Instruction	n	%	Total
Aucune	3326	77,52	4290
Primaire	1380	78,36	1761
Secondaire	531	78,20	679
**Universitaire**	152	**87,35**	174

Prévalence de la consommation insuffisante de fruits et de légumes selon l'ethnie

La plus grande prévalence de consommation journalière insuffisante de fruits et de légumes était retrouvée chez les sujets d'ethnie Dendi.

Le tableau XXXII montre la prévalence de la consommation insuffisante de fruits et de légumes selon l'ethnie.

Tableau XXVI : Répartition des sujets selon la consommation insuffisante de fruits et légumes et l'ethnie au Bénin en 2008

Ethnie	< 5 portions		≥ 5 portions		total
	n	%	n	%	
Adja	856	81,52	194	18,48%	1050
Fon	2093	75,58	676	24,42	2769
Bariba	728	80,53	176	19,47	904
**Dendi**	317	**94,62**	18	05,38	335
Yom	203	81,2	47	18,80	250
Peuhl	212	77 ,09	63	22,91	275
Otamari	174	71,60	69	28,40	243

Yoruba	743	75,97	235	24,03	978
Autres	63	63,0	37	37,00	100

Prévalence de la consommation insuffisante de fruits et de légumes selon la catégorie professionnelle

La plus grande prévalence de consommation journalière insuffisante de fruits et légumes était retrouvée chez les sujets retraités (96,52%).

Le tableau XXXIII montre la prévalence de la consommation insuffisante de fruits et de légumes selon la catégorie professionnelle.

Tableau XXXIII : Répartition des sujets selon la consommation insuffisante de fruits et de légumes et la catégorie professionnelle au Bénin en 2008

**Profession**	**< 5 portions**		**≥ 5 portions**		**Total**
	**n**	**%**	**n**	**%**	
E. Etat	200	88,88	25	11,12	225
E. Privé	230	85,50	39	14,5	269
Indépendant	4159	76,87	1251	23,12	5410
Etudiant	92	90,19	10	09,80	102
Maître. M	540	83,59	106	16,40	646
Retraité	111	**96,52**	4	03,47	115
Chômeur	57	41,60	80	41,60	137

5.5.1 Nombre moyen de portions de fruits ou de légumes consommées par jour

Chez les 6575 sujets qui consommaient des fruits, la moyenne de portions consommées par jour était de 2,10 portions. Chez les 6749 sujets qui consommaient des légumes, la moyenne de portions consommées par jour était de 1,58 portion. Chez les 6787 sujets qui consommaient les fruits et/ou les légumes, la moyenne de portions consommées par jour était de 3,10.

Le tableau XXXIV montre le nombre moyen de portions de fruits et/ou de légumes consommés par jour selon l'âge.

Tableau XXVII: Nombre moyen de portion de fruits et/ ou de légumes consommés par jour selon l'âge au Bénin en 2008

**Tranche d'âge**	**Fruits**		**Légumes**		**Fruits et légumes**	
	**n**	**M**	**n**	**M**	**n**	**M**
25-34	2088	2,20	2125	**1,52**	2137	3,67
35-44	1605	2,24	1644	1,68	1647	3,86
45-54	1121	2,11	1148	1,55	1160	3,58
55-64	1761	**1,85**	1832	1,56	1843	**3,32**
**25-64**	6575	2,10	6749	1,58	6787	3,60

M = nombre moyen de portion n = effectif

5.5.2 Nombre moyen de jours de consommation de fruits ou de légumes

Chez les 6575 sujets qui consommaient des fruits, la moyenne de jours de consommation hebdomadaire était de 2,90 jours. Chez les 6749 sujets qui consommaient des légumes, la moyenne de jours de consommation hebdomadaire était de 4,24 jours.

Le tableau XXXV montre le nombre moyen de jours de consommation hebdomadaire de fruits ou de légumes.

TableauXXXV : Nombre moyen de jours de consommation de fruits ou de légumes par semaine selon l'âge au Bénin en 2008

**Tranche d'âge**	**Fruits**			**Légumes**		
	**n**	**N**	**ect**	**n**	**N**	**ect**
25-34	2088	2,98	2,1058	2125	**4,15**	2,0823
35-44	1605	2,95	2,0684	1644	4,25	2,0428
45-54	1121	2,81	2,0786	1148	4,32	1,9982
55-64	1761	**2,78**	2,0148	1832	4,27	1,9679
**Total**	6575	2,89	2,0693	6749	4,24	2,0284

N = nombre moyen de jours ect. = écart-type

STEP II

5.6 Prévalence de la tension artérielle élevée au Bénin en 2008

Parmi les 6904 sujets enquêtés, 1899 avaient présenté une TA ≥ 140/90mmHg, soit une prévalence de 27,50 % [IC 95% :26,46% - 28 ,58%].

Le tableau XXXVI montre la prévalence de la TA élevée en fonction du sexe, du milieu de résidence et de l'âge.

Neuf cent quarante neuf (949) sujets avaient une tension artérielle élevée parmi les 3485 sujets de sexe masculin, soit une prévalence de 27,23 %. Dans le groupe des 3419 sujets de sexe féminin, 899 avaient une TA élevée, soit une prévalence de 26,29 %. La prévalence de la TA élevée parait plus élevée chez les hommes, mais cette différence n'est pas statistiquement significative (p=0,3).

Six cents quatre vingt dix sept (697) sujets avaient une TA élevée en milieu urbain (29,54%) versus 1202 en milieu rural (26,44%). La prévalence de la tension artérielle élevée était significativement plus élevée chez les sujets vivant en zone urbaine ($p < 10^{-2}$).

La prévalence de la tension artérielle élevée augmentait significativement avec l'âge (p <10-8).

Tableau XXVIII : Prévalence de la TA élevée en fonction du sexe, du milieu de résidence et de l'âge au Bénin en 2008

Variables	TA élevée		TA normale	Total
	n	%	n	
**Sexe**				
Masculin	949	**27,23**	2536	3485
Féminin	899	26,29	2520	3419
**Milieu de résidence**				
Rural	1202	26,44	3343	4545
Urbain	697	**29,54**	1662	2359
**Age**				
25-34	285	13,2	1867	2167
35-44	404	24,1	1258	1677
45-54	406	**34,4**	759	1179
55-64	804	2,7	1063	1881
**Total**	1899	27,51	5005	6904

Sur l'effectif des 6904 personnes enquêtées, 1899 sujets avaient une TA élevée et 62 prenaient des médicaments antihypertenseurs, soit une prévalence de 28,4% (1961 /6904). Cette prévalence était de 28,6 % chez les 3485 sujets de sexe masculin et de 28,3 p. cent chez les 3419 de sexe féminin. (p=0, 9).

Le tableau XXXVII montre la prévalence de la tension artérielle élevée en tenant compte du traitement antihypertenseur en fonction de l'âge. Cette prévalence augmentait significativement avec l'âge ($p<10^{-8}$).

Tableau XXIX : Prévalence de la tension artérielle élevée en tenant compte de la prise d'antihypertenseur en fonction de l'âge au Bénin en 2008

Age	TA élevée et traitement antiHTA		TA normale sans traitement antiHTA	Total
	n	%	n	
25-34	291	13,4	1876	2167
35-44	461	27,5	1216	1677
45-54	418	35,5	761	1179
55-64	791	**42,1**	1090	1881
**Total**	1961	28,4	4943	6904

Le tableau XXXVIII résume l'ensemble des informations relatives à la prévalence de la tension artérielle élevée en fonction du niveau d'instruction, de l'activité professionnelle et de l'ethnie.

Il n'y a pas d'association significative entre la TA élevée et le niveau d'instruction (p=0,3). La TA élevée était significativement plus importante chez les sujets invalides ($p<10^{-4}$) et chez les sujets d'ethnie yoruba ($p=10^{-8}$).

Tableau XXX : prévalence de la tension artérielle élevée en fonction du niveau d'instruction, de l'activité professionnelle et de l'ethnie au Bénin en 2008

Variables	TA élevée		TA normale	Total	p
	n	%	n		
**Niveau d'instruction**					
Aucune instruction	1200	28,0	3090	4290	0,3
primaire	498	**28,3**	1263	1761	
secondaire	166	24,4	513	679	
universitaire	35	20,1	139	174	
**Activité professionnelle**					
Employé de l'Etat	61	26,7	169	230	$< 10^{-4}$
Employé du Privé	72	25,9	206	278	
Indépendant	1466	27,1	3942	5408	
Bénévole	4	15,4	22	26	
Etudiant	17	16,8	84	645	
Maitre (esse)	193	29,9	452	114	
Retraité	47	41,2	67	101	
Chômeur	13	26,5	36	49	
Invalide	26	**49,1**	27	53	
**Ethnie**					
Adja	320	30,5	730	1050	$< 10^{-8}$
Fon	773	27,9	1996	2769	
Bariba	230	25,4	674	904	
Dendi	99	29,6	236	335	
Yom	53	21,2	197	250	
Peuhl	53	19,3	222	275	
Otamari	37	15,2	206	243	
Yoruba	316	**32,3**	662	978	
Autres	18	18,0	82	100	
**Total**	1899	27,51	5005	6904	

Prévalence de la tension artérielle élevée en fonction des départements du Bénin en 2008

Le tableau XXXIX résume la prévalence de la TA élevée en fonction des différents départements du Bénin. Cette prévalence était significativement plus élevée dans le département de l'Ouémé ($p=10^{-5}$).

Tableau XXXI : prévalence de la tension artérielle élevée en fonction des différents départements du Bénin en 2008

Départements	Effectif	TA élevée	Prévalence (%)
**Ouémé**	**693**	**269**	**38,82**
Plateau	467	150	32,12
Atlantique	914	220	24,07
Littoral	585	150	25,64
Mono	344	123	35,76
Couffo	574	170	29,62
Zou	687	158	23,00
Collines	572	164	28,67
Borgou	687	161	23,44
Alibori	462	112	24,24
Atacora	574	133	23,17
Donga	345	89	25,80

Répartition des sujets en fonction des différents stades de la tension artérielle élevée de l'OMS au Bénin en 2008

Le tableau XL résume la répartition des sujets enquêtés en fonction des différents stades de l'hypertension artérielle de l'OMS. Plus de deux sujets sur trois avaient une TA élevée au stade 1.

Tableau XXXII : Répartition des sujets enquêtés selon les différents stades de la TA élevée de l'OMS au Bénin en 2008

Stades de TA élevée	n	%
Stade 1	1352	**71,2**
Stade 2	439	23,1
Stade 3	108	5,7
**Total**	1899	100,0

Répartition des sujets ayant une TA élevée en fonction de la consommation de fruits et légumes au Bénin en 2008

Parmi les 1899 sujets ayant une TA élevée, 1507 consommaient moins de cinq portions de fruits et légumes, soit une proportion de 79,35%. Cette répartition est résumée dans le tableau XLII.

Tableau XXXIII: Répartition des sujets ayant une TA élevée en fonction de la consommation de fruits et légumes (FL) au Bénin en 2008

Variables	TA élevée	
	**n**	**%**
FL≥5	392	20,65
FL<5	1507	79,35
Total	100,0	1899

Répartition des sujets ayant une TA élevée en fonction de la consommation nocive d'alcool au Bénin en 2008

Parmi les 1899 sujets ayant une TA élevée, 27 consommaient une quantité nocive d'alcool, soit une proportion de 1,42%. Le tableau XLII résume les informations relatives à cette répartition.

Tableau XXXIV : Répartition des sujets ayant une TA élevée en fonction de la consommation nocive d'alcool au Bénin en 2008

Variables	TA élevée	
	**n**	**%**
Consommation nocive	27	1,42
Abstinents	1872	**98,58**
Total	1899	100,00

Répartition des sujets ayant une TA élevée en fonction de l'obésité au Bénin en 2008

Trois cents (300) sujets parmi les 1899 ayant une TA élevée étaient obèses, soit une proportion de 15,8%. Voir tableau XLIII.

Tableau XXXV: Répartition des sujets ayant une TA élevée en fonction de l'obésité au Bénin en 2008

Variables	TA élevée	
	**n**	**%**
Obèses	300	15,8
Non obèses	1599	84,2
total	1899	100,0

Répartition des sujets enquêtés au Bénin en 2008 selon qu'ils aient reçu des conseils relatifs au mode de vie par un médecin ou un agent de santé pour une TA élevée

Parmi les sujets enquêtés qui avaient une TA élevée, environ un sur deux (48,60%) avait reçu des conseils relatifs au régime alimentaire et chez environ 1 sujet sur 4 (23,70%) une perte de poids avait été conseillée. La figure2 résume l'ensemble de ces informations.

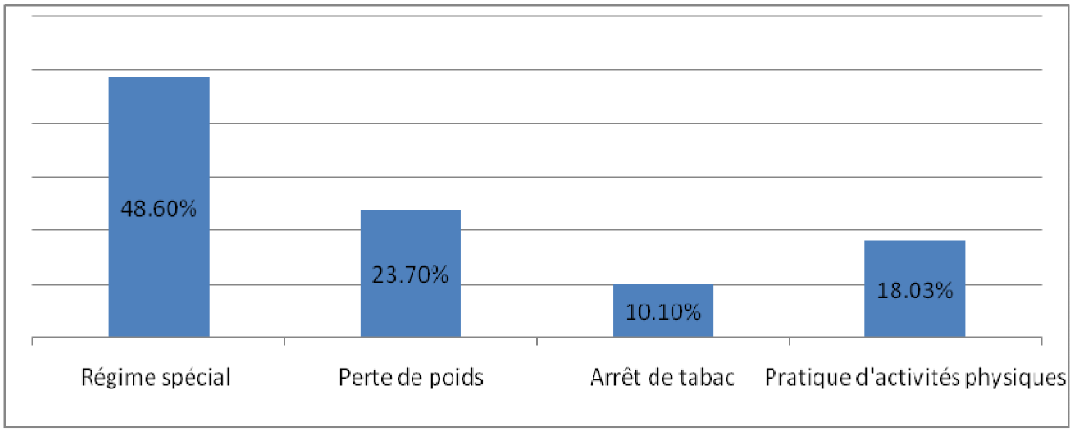

Figure 2 : Répartition des sujets enquêtés selon qu'ils aient reçu des conseils relatifs au mode de vie par un médecin ou un agent de santé pour une TA élevée.

Répartition des sujets enquêtés présentant une tension artérielle élevée en fonction de leurs antécédents au Bénin en 2008

Le tableau XLIV résume l'ensemble des informations relatives aux antécédents des sujets présentant une tension artérielle élevée. Plus de trois sujets sur quatre ne se savaient pas hypertendus (77,5%). Plus de quatre sur

cinq avaient rapporté que leur dernière prise de la TA a été effectuée depuis plus de 10 ans. 81,6% d'entre eux n'avaient pas pris leur médicament antihypertenseur durant les deux dernières semaines qui ont précédé l'enquête.

Tableau XXXVI: Répartition des sujets enquêtés ayant une TA élevée en fonction de leurs antécédents au Bénin en 2008

**Antécédents**	**n**	**%**
**Connaissance antérieure de la TA Elevée ces 12 derniers mois**		
Oui	416	22,5
Non	1483	**77,5**
**Dernière prise de la TA**		
≤ 12 mois	580	30,6
Entre 1et 5 ans	540	28,4
>10 ans	779	**41,0**
**Prise de médicaments antiHTA au cours des deux dernières semaines**		
Oui	349	18,4
Non	1550	**81,6**
**total**	1899	100,0

5.7 Prévalence de la surcharge pondérale et de l'obésité au Bénin en 2008

5.7.1 Prévalence de l'obésité

Sur l'effectif des 6904 sujets enquêtés, 649 étaient obèses soit une prévalence 9,40% [IC95% : 8,72%-10,12%]. Dans le groupe des 3485 sujets de sexe masculin, 151 étaient obèses, soit une prévalence de 4,33%. Parmi les 3419 sujets de sexe féminin, 479 avaient une obésité, soit une prévalence de 14,01%. Les femmes étaient significativement plus obèses que les hommes ($p=10^{-7}$).

Au sein des 4545 sujets résidant en milieu urbain, 533 étaient obèses (11,7%) versus (4,9%) en milieu rural (116/2359). La prévalence de l'obésité était significativement plus élevée en milieu urbain ($p< 10^{-8}$).

Parmi les 649 sujets obèses, 90 avaient une obésité morbide, soit une proportion de 13,8%, 253 avaient une obésité androïde, soit une proportion de 38,7%, 396 avaient une obésité gynoïde, soit une proportion de 61%

Dans l'étude, 2437 personnes sur les 6904 enquêtées avaient une obésité abdominale, soit une prévalence de 35,3%.

Le tableau XLV montre la prévalence de l'obésité en fonction de l'âge. Cette prévalence augmentait significativement avec l'âge jusqu'à 54 ans ($p<10^{-8}$).

Tableau XXXVII: Prévalence de l'obésité en fonction de l'âge au Bénin en 2008

Age	Obèses		Non obèses	Total
	n	%	n	
25-34	131	6,04	2036	2167
35- 44	125	7,45	1552	1677
45- 54	172	**14,58**	1007	1179
55- 64	221	11,75	1660	1881
**Total**	649	9,40	6255	6904

Prévalence de l'obésité en fonction du niveau d'instruction, de l'activité professionnelle et de l'ethnie au Bénin en 2008

La prévalence de l'obésité était significativement plus élevée chez les sujets ayant un niveau secondaire d'instruction (10^{-8}), les bénévoles (10^{-8}) et chez les sujets d'ethnie Fon (10-8). Voir tableau XLVI.

Tableau XXXVIII: prévalence de l'obésité en fonction du niveau d'instruction, de l'activité professionnelle et de l'ethnie au Bénin en 2008

Variables	Obèses		Non obèses	Total	p
	n	%	n		
**Niveau d'instruction**					
Aucun	325	7,6	3965	4290	10^{-8}
Primaire	208	11,8	1553	1761	
**Secondaire**	101	**14,8**	578	679	
Universitaire	15	8,6	159	174	
**Profession**					
Employé de l'Etat	21	9,1	209	230	10^{-8}
Employé du Privé	23	8,3	255	278	
Indépendant	501	9,3	4907	5408	
**Bénévole**	6	**23,1**	20	26	
Etudiant	5	5,0	96	101	
Maîtresse de maison	70	10,9	575	645	
Retraité	14	12,3	100	114	
Chômeur	3	6,1	46	49	
Invalide	6	11,3	47	53	
**Ethnie**					
Adja et apparentés	98	10,3	952	1050	10^{-8}
Fon et apparentés	305	11,0	2464	2769	
Bariba et apparentés	60	6,6	842	904	
Dendi et apparentés	26	7,8	309	335	
Yom	10	4,0	240	250	
Peuhl	11	4,0	264	275	
**Otamari**	27	**11,1**	216	243	
Yoruba	108	11,0	870	978	
Autres	4	4,0	96	100	
**Total**	649	9,4	6255	6904	

Prévalence de l'obésité en fonction des départements au Bénin en 2008

Le tableau XLVII résume la prévalence de l'obésité dans les différents départements du Bénin. Cette prévalence était significativement élevée dans le département du Littoral ($p=10^{-8}$).

Tableau XXXIX: prévalence de l'obésité en fonction des départements au Bénin en 2008

**Départements**	**obésité**		**Total**
	**n**	**%**	
Ouémé	90	13,0	693
Plateau	51	10,9	467
Atlantique	104	11,4	914
**Littoral**	115	**19,7**	585
Mono	19	5,5	344
Couffo	47	8,2	574
Zou	45	6,6	687
Collines	60	10,5	572
Borgou	55	8,0	687
Alibori	15	3,2	462
Atacora	29	5,1	574
Donga	19	5,5	345
Total	649	9,4	6904

5.7.2 Prévalence de la surcharge pondérale

Parmi les 6904 sujets enquêtés, 1415 étaient en surpoids, soit une prévalence de 20,50% [IC95% :19,55%-21,47%]. Au sein des 3485 sujets de sexe masculin, 587 avaient une surcharge pondérale soit une prévalence de 16,8%. Les 788 sujets de sexe féminin en surcharge pondérale représentaient 23,04 %. La prévalence de la surcharge pondérale était significativement plus élevée chez les sujets de sexe féminin ($p = 10^{-7}$).

Le tableau XLVIII montre la prévalence de la surcharge pondérale en fonction de l'âge. Cette prévalence augmentait significativement avec l'âge jusqu'à 54 ans ($p<10^{-4}$).

Tableau XL: prévalence de la surcharge pondérale en fonction de l'âge au Bénin en 2008

**Age**	**Surpoids +**		**Surpoids -**	**Total**
	**n**	**%**	**n**	
25-34	302	13,9	1865	2167
35- 44	401	23,9	1276	1677
**45- 54**	318	**27,0**	861	1179
55- 64	394	20,9	1487	1881
**Total**	1415			6904

Prévalence de la surcharge pondérale en fonction de l'activité professionnelle et de l'ethnie au Bénin en 2008

Le tableau XLIL résume les informations relatives à la prévalence de la surcharge pondérale en fonction de l'activité professionnelle et de l'ethnie. Elle était significativement plus élevée chez les sujets bénévoles ($p<10^{-8}$) et chez les sujets, d'ethnie dendi ($p<10^{-4}$).

Tableau XXLIIIL: Prévalence de la surcharge pondérale en fonction de l'activité professionnelle et de l'ethnie au Bénin en 2008

Variables	Surpoids+ (%)		Surpoids(-)	Total	p
	n	%	n		
**Profession**					10^{-8}
Employé de l'Etat	74	32,1	156	230	
Employé du Privé	75	26,9	203	278	
Indépendant	1050	19,4	4358	5408	
**Bénévole**	17	**65,4**	9	26	
Etudiant	11	10,9	90	101	
Maitre(sse) maison	134	26,2	511	645	
Retraité	30	26,3	84	114	
Chômeur	7	14,3	42	49	
Invalide	17	32,1	36	53	
**Ethnie**				1050	10^{-4}
Adja et apparentés	202	19,2	848		
Fon et apparentés	584	21,1	2185	2769	
Bariba	183	20,2	721	904	
**Dendi**	80	**23,9**	255	335	
Yom	35	14,0	215	250	
Peuhl	34	12,4	241	275	
Otamari	32	13,2	211	243	
Yoruba	209	21,4	769	978	
Autres	16	16,0	84	100	
**Total**	1415	20,5	5489	6904	

5.7.3 Répartition des sujets enquêtés en fonction de l'indice de masse corporelle au Bénin en 2008

La figure 3 montre la répartition des sujets enquêtés en fonction de leur indice masse corporelle.

Plus d'un sujet sur 5 (20,5%) avaient une surcharge pondérale et moins d'un sujet sur dix (8,10%) était maigre.

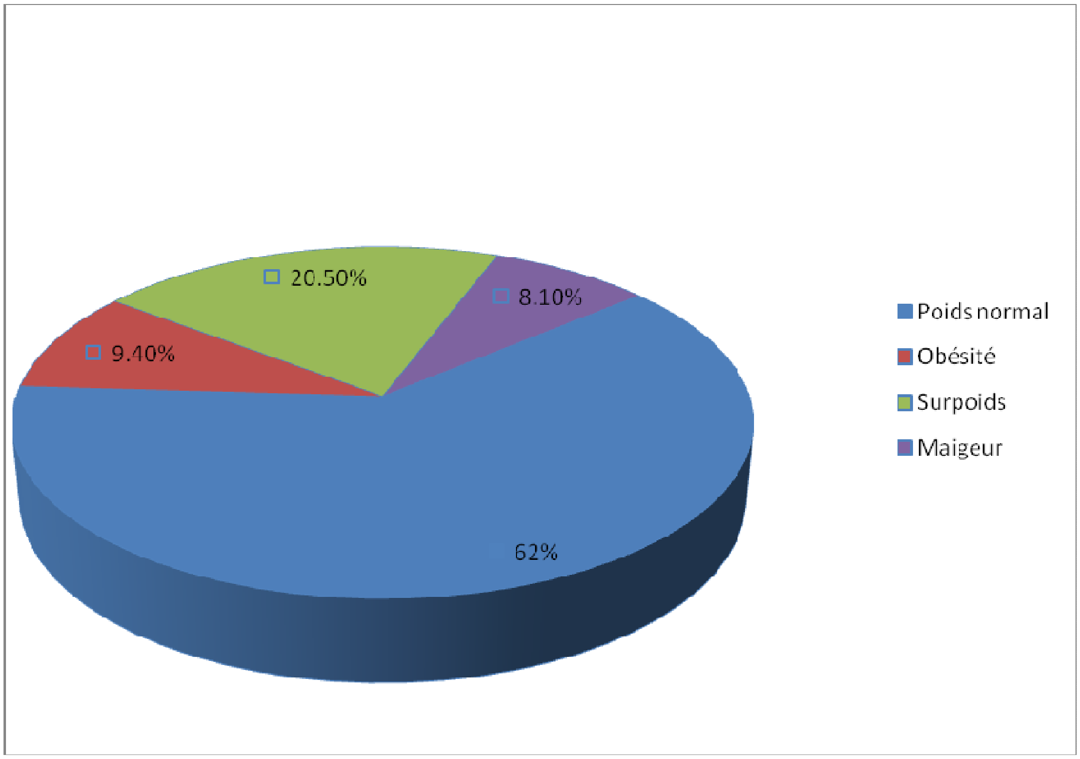

Figure 3: Répartition des sujets enquêtés en fonction de l'indice de masse corporelle au Bénin en 2008

STEP III

5.8 Prévalence de l'hyperglycémie

Sur les 3822 sujets prélevés, 101 avaient une hyperglycémie de type diabétique soit une prévalence de 2,6% [IC 95% = 2,2-3,2].

Parmi ces derniers, 74 soit 73,27% ne se savaient pas diabétiques. La glycémie à jeun anormale avait été retrouvée chez 110 sujets soit 2,9% [IC 95% ; 2,4-3,4].

Sur 1854 sujets de sexe masculin, 61 avaient une hyperglycémie de type diabétique soit 3,3% versus 2,0% (40 /1968) chez les sujets de sexe féminin ($p< 0,05$).

Sur 1332 sujets résidant en milieu urbain ; 51 avaient une hyperglycémie soit une prévalence de 3,8% versus 2,0% en milieu rural (50 sujets sur 2490) ;($p< 10^{-3}$).

Le Tableau XLIII montre la prévalence de la glycémie anormale à jeun et de l'hyperglycémie de type diabétique en fonction de l'âge. La prévalence de l'hyperglycémie de type diabétique est significativement plus élevée chez les sujets de 45 à54 ans ($p< 10^{-3}$). En ce qui concerne la glycémie anormale à jeun, il n'a pas pu être mis en évidence une différence significative entre le taux de glycémie et l'âge des patients (p=0,4)

Tableau XLIII: Prévalence de l'hyperglycémie et de la glycémie anormale a jeun en fonction de l'âge au Bénin en 2008.

**Tranche d'âge (ans)**	**Hyperglycémie jeun anormale**		**Hyperglycémie de type diabétique**		**Total**
	**n**	**%**	**n**	**%**	
25 – 34	28	2,5	19	1,7	1112
35 – 44	34	3,6	15	1,6	935
45 – 54	18	2,6	27	3,9	699
**55 – 64**	30	**2,8**	40	**3,7**	1076
**Total**	110	2,9	101	2,6	3822

Prévalence de l'hyperglycémie de type diabétique et de la glycémie anormale à jeun en fonction du département au Bénin en 2008

Le Tableau XLIV résume la prévalence de la glycémie à jeun anormale et de l'hyperglycémie de type diabétique dans les douze départements. La prévalence la plus élevée a été observée dans le département du Borgou (4,6%) et la plus faible a été étaient retrouvée dans les départements de l'Ouémé (0,9%).

Tableau XLIV : Prévalence de la glycémie à jeun anormale et de l'hyperglycémie de type diabétique en fonction du département au Bénin en 2008.

Départements	Hyperglycémie à jeun anormale		Hyperglycémie de type diabétique		Total
	n	%	n	%	
**Alibori**	26	**7,9**	8	2,4	330
Atacora	19	6,0	12	3,8	316
Atlantique	9	1,7	15	2,9	514
**Borgou**	17	4,6	17	**4,6**	373
Collines	8	2,2	9	2,5	358
Couffo	9	2,7	8	2,4	333
Donga	4	2,5	5	3,1	162
Littoral	26	7,9	8	2,4	330
Mono	2	1,2	2	1,2	172
Ouémé	5	1,2	4	0,9	422
Plateau	1	0,4	6	2,6	227
Zou	3	0,8	10	2,7	371
Total	110	2,9	101	2,6	3822

Prévalence de l'hyperglycémie au Bénin selon l'ethnie

Le tableau LII résume la prévalence de la glycémie à jeun anormale et de l'hyperglycémie selon l'ethnie. La prévalence d'hyperglycémie la plus forte a été observée chez les sujets d'ethnie Otamari (6,8%).

Tableau XLV: Prévalence de la glycémie anormale à jeun et de l'hyperglycémie de type diabétique au Bénin en 2008

**Ethnie**	**Glycémie à jeun anormale**		**Hyperglycémie de type diabétique**		**Total**
	**n**	**%**	**n**	**%**	
Adja	15	2,6	12	2,1	581
Fon	41	2,6	35	2,2	1582
Bariba	27	**5,6**	15	3,1	483
Dendi	5	2,9	6	3,4	173
Yom	4	3,3	4	3,3	120
Peulh	4	2,6	3	2,0	151
**Otamari**	7	5,2	8	**6,8**	133
Yoruba	7	1,2	13	2,3	558
Africain de l'ouest	0	0	0	0	41
**Total**	110	2,9	101	2,6	3822

Prévalence l'hyperglycémie selon le niveau d'instruction

Sur les 92 sujets de niveau universitaire, 6 avaient une hyperglycémie, soit 6,5% versus 1,8% chez les sujets n'ayant aucune instruction officielle. La prévalence de l'hyperglycémie croît significativement avec le niveau d'instruction

($p < 0,05$). La prévalence de l'hyperglycémie de type diabétique et de la glycémie anormale à jeun selon le niveau d'instruction est présentée dans le Tableau XLVI.

Tableau XLVI: Prévalence de la glycémie à jeun anormale et de l'hyperglycémie de type diabétique selon le niveau d'instruction au bénin en 2008.

**Niveau d'instruction**	**Glycémie à jeun anormal**		**Hyperglycémie de type diabétique**		**Total**
	**n**	**%**	**n**	**%**	
Aucune	56	2,4	43	1,8	2350
primaire	29	3,0	30	3,0	983
**Secondaire**	21	**4,8**	22	5,5	397
Université	4	4,3	6	**6,5**	92
**Total**	110	2,9	101	2,6	3822

Prévalence de l'hyperglycémie de type diabétique et de la glycémie anormale à jeun selon l'activité professionnelle

Le Tableau XLVII résume la prévalence de la glycémie à jeun anormale et de l'hyperglycémie de type diabétique selon l'activité professionnelle. Parmi les 34 sujets invalides sur le plan professionnel, 3 avaient une hyperglycémie ; soit une prévalence de 8,8% versus 0% chez les sujets bénévoles. La différence observée est significative ($p<10^{-3}$).

Tableau XLVII: Prévalence de la glycémie à jeun anormale et de l'hyperglycémie de type diabétique selon l'activité professionnelle au Bénin en 2008.

**Activité professionnelle**	**Glycémie anormale à jeun**		**Hyperglycémie de type diabétique**		**Total**
	**n**	**%**	**n**	**%**	
Employé(e) de l'état	7	5,1	10	7,2	138
Employé(e) du privé	1	0,8	4	3,1	130
Indépendant(e)	86	2,9	74	2,5	2984
Bénévole	0	0	0	0	13
Etudiant(e)	1	1,9	2	3,8	52
Maitre(sse)de maison	11	3,1	3	0,8	358
Retraité(e)	0	0	3	3,8	79
**Chômeur**	2	**5,9**	2	5,9	34
**Invalide**	1	2,9	3	**8,8**	34
**Total**	110	2,9	101	2,6	3822

5.9 Prévalence de l'hypercholestérolémie

Sur 3822 sujets prélevés, 68 sujets ont une hypercholestérolémie soit 1,8% [IC 95% =1,4-2,2]. La cholestérolémie totale élevée a été retrouvée chez 233sujets soit 6,1% [IC 95% =5,4-6,9].

Parmi les 1968 sujets de sexe féminin, 38 avaient une hypercholestérolémie soit 1,9% versus 1,6% chez les sujets de sexe masculin, (p=0,46).

Sur 1332 sujets résidant en zone urbaine, 26 avaient une hypercholestérolémie soit une prévalence de 1,9% versus 1,7% en zone rurale (42sujets sur 2490) p<10-3.

Prévalence de l'hypercholestérolémie et de la cholestérolémie totale élevée en fonction de l'âge au Bénin en 2008.

Sur 935 sujets de la tranche d'âge de 35 à 44 ans, 15 avaient une l'hypercholestérolémie soit une prévalence de 1,6%. La prévalence croît significativement avec l'âge (P= 0,02). Le Tableau XLVIII résume Prévalence de l'hypercholestérolémie et de la cholestérolémie totale en fonction de l'âge

Tableau XLVIII: Prévalence de l'hypercholestérolémie et de cholestérolémie totale élevée en fonction de l'âge au Bénin en 2008

**Tranches d'âges (ans)**	**CT élevé**		**hypercholestérolémie**		**Total**
	**n**	**%**	**n**	**%**	
25-34	30	2,7	10	0,9	1112
35-44	65	7,0	15	1,6	935
45-54	48	6,9	15	2,1	699
55-64	90	**8,4**	28	**2,6**	1076
**Total**	233	6,1	68	1,8	3822

Prévalence de l'hypercholestérolémie et de cholestérolémie totale élevée en fonction du département au Bénin en 2008

Le Tableau XLIX résume la prévalence de l'hypercholestérolémie en fonction du département. La prévalence la plus élevée a été observée dans le département du Zou (3,0 %).

Tableau XLIX: Prévalence l'hypercholestérolémie par département au Bénin en 2008

**Département**	**CT élevé**		**Hypercholestérolémie**		**Total**
	**n**	**%**	**n**	**%**	
Plateau	8	3,5	4	1,8	227
Atlantique	44	8,6	9	1,7	514
Alibori	4	1,6	5	2,0	244
**Zou**	12	3,2	11	**3,0**	371
Littoral	31	9,4	5	1,5	330
Mono	16	9,3	2	1,2	172
**Ouémé**	40	**9,5**	8	1,9	422
Couffo	24	7,2	6	1,8	333
Collines	18	5,0	9	2,5	358
Borgou	22	5,9	3	0,8	373
Atacora	5	1,6	2	0,6	316
Donga	9	5,6	4	2,5	162
**Total**	233	6,1	68	1,8	3822

Prévalence de l'hypercholestérolémie selon l'ethnie au Bénin en 2008

La prévalence de l'hypercholestérolémie selon l'ethnie est présentée dans le Tableau L . La prévalence la plus forte est observée chez les sujets d'ethnie Dendi.

Tableau L: Prévalence de l'hypercholestérolémie au Bénin selon l'ethnie

Ethnie	CT élevée		hypercholestérolémie		Total
	n	%	N	%	
Adja	41	7,1	8	1,4	581
**Fon**	115	**7,3**	31	2,0	1582
Bariba	20	4,1	5	1,0	483
Dendi	9	5,2	4	2,3	173
Yom	3	2,5	1	0,8	120
Peulh	2	1,3	2	1,3	151
Otamari	2	1,5	2	1,5	133
**Yorouba**	38	6,8	15	**2, 7**	558
Ouest-africain	3	**7,3**	0	0	41
Autres	0	0	0	0	0
**Total**	233	6,1	68	1,8	3822

Prévalence selon le niveau d'instruction au Bénin en 2008

La prévalence de l'hypercholestérolémie est de 1,6% chez les sujets sans instruction officielle. Cette prévalence croissait avec le niveau d'instruction mais une inflexion a été observée chez les sujets de niveau universitaire p= 0,5.

Le Tableau LI résume la prévalence de l'hypercholestérolémie selon le niveau d'instruction.

Tableau LI: Prévalence de l'hypercholestérolémie selon le niveau d'instruction au Bénin en 2008

Niveau d'instruction	CT élevé		Hypercholestérolémie		Total
	n	%	n	%	
Aucune	125	5,3	38	1,6	2350
Primaire	63	6,4	19	1,9	983
**Secondaire**	35	8,8	10	**2,5**	397
**Université**	10	**10,9**	1	1,1	92
**Total**	233	6,1	68	1,8	3822

Prévalence selon l'activité professionnelle

La prévalence est plus élevée chez les invalides (5,9 %) suivie par les retraités (3,8 %). Le Tableau LII résume la prévalence de l'hypercholestérolémie selon l'activité professionnelle (p=0,13).

Tableau LII: Prévalence de l'hypercholestérolémie selon l'activité professionnelle

**Activité professionnelle**	**CT élevé**		**Hypercholestérolémie**		**Total**
	**n**	**%**	**n**	**%**	
Employé de l'état	12	8,7	4	2,9	138
Employé du privé	7	5,4	2	1,5	130
Indépendant	172	5,8	46	1,5	2984
Bénévole	0	0	0	0	13
Etudiant	1	1,9	0	0	52
Maitre de maison	24	6,7	11	3,1	358
Retraité	8	10,1	3	3,8	79
**Chômeur**	5	**14,7**	0	0	34
**Invalide**	3	8,8	2	**5,9**	34
**Total**	233	6,1	68	1,8	3822

Conclusion

Au terme de notre étude, les conclusions suivantes peuvent être dégagées:

- Une proportion non négligeable de la population béninoise est exposée au tabagisme et à la consommation nocive d'alcool.
- Le sexe masculin, l'âge, le niveau d'instruction bas, le milieu rural et le milieu socioculturel sont des facteurs favorisant le tabagisme et la consommation nocive d'alcool au Bénin.
- Le tabagisme favorise la consommation nocive d'alcool et inversement, la consommation nocive d'alcool favorise le tabagisme.
- La prévalence de l'inactivité physique était de 8,3% et celle de la consommation insuffisante de fruits et légumes était de 78,05%.
- Un adulte sur quatre a une tension artérielle élevée et environ un sujet sur dix est obèse.

Le système sanitaire devra faire face au fardeau grandissant lié à ces facteurs de risque, en s'impliquant effectivement dans leur prévention qui constitue la 1ère arme contre les MNT. Il s'avère donc important de suivre l'évolution de ces FDR et d'élaborer des plans stratégiques pour les réduire.

Recommandations

A l'issue de notre étude nous formulons les suggestions suivantes :

A l'endroit des autorités politico-administratives

- **Ministère de la santé**

  - ✓ Introduire le dépistage des 8 principaux facteurs de risque des maladies non transmissibles dans les visites médicales chez les enseignants, les étudiants et les élèves.
  - ✓ Investir dans la formation des médecins spécialisés dans les maladies non transmissibles.
  - ✓ Créer des centres de sevrage pour les personnes ayant une dépendance alcoolo-tabagique et veiller à leur réinsertion socioprofessionnelle.
  - ✓ Sensibiliser la population sur les bienfaits de la consommation de fruits et de légumes et le caractère peu coûteux de l'activité physique.
  - ✓ Créer des environnements favorables à la pratique de l'activité physique de loisir ;
  - ✓ Associer les médecins nutritionnistes dans un proche avenir à la prévention des MNT ;
  - ✓ promouvoir des jardins scolaires pour la production des légumes ;
  - ✓ Veiller à l'accessibilité géographique et financière des fruits et légumes à tous.

- **Ministères de l'intérieur et de la santé**

- ✓ Œuvrer pour la mise en application de l'article 8 de la loi cadre antitabac portant création de lieux non fumeurs.

- **Ministères de l'intérieur et du commerce**
  - ✓ Créer des lois qui régularisent la production, la commercialisation et la consommation des boissons alcoolisées.
  - ✓ Amener les fabricants de boissons alcoolisées locales à renter dans le secteur formel.

- **Ministères de l'enseignement**
  - ✓ Sensibiliser par des modules de formation les élèves et étudiants sur les méfaits du tabagisme et de la consommation abusive d'alcool.
  - ✓ Initier une recherche pour déterminer les facteurs favorisant la faible prévalence de la TA élevée et de l'obésité chez Peuhls.

- **A l'endroit des agents socio-sanitaires**

- ✓ Introduire la surveillance des 8 principaux facteurs de risque des maladies non transmissibles dans leur pratique quotidienne.
- ✓ Sensibiliser les patients sur les facteurs de risque des MNT en général et sur la consommation de fruits et légumes et l'activité physique en particulier.

- ✓ d'insister sur les aspects bénéfiques de la perte de poids (mieux-être physique, harmonisation du corps sans perdre ses courbes, meilleure image de soi)
- ✓ d'apporter des informations nutritionnelles claires (repas riches en fruits et légumes, pauvres en sel, sucre et graisses). Les spécialistes en

nutrition peuvent être mis à contribution pour élaborer des types de menu en tenant compte des ressources locales.

- ✓ Prendre systématiquement la TA, le poids, la taille, le tour de taille à chaque première consultation.
- ✓ Conseiller aux patients de contrôler leur TA au moins une fois tous les trois mois.
- ✓ Créer un modèle qui promeut la lutte contre l'obésité ;
- ✓ Organiser des séances de dépistage des FDR en population générale ;
- ✓ Inscrire la lutte contre les MNT dans les priorités en matière de Santé ;
- ✓ Doter le PNLMNT de ressources nécessaires pour assurer une lutte efficace contre ces FDR.

- **A l'endroit de la population**

- ✓ Pratiquer une activité physique d'intensité modérée au moins 30 minutes 3 fois par semaine. Cette habitude ne doit pas être abandonnée après les 2 premiers niveaux scolaires (primaire, secondaire) ou avec l'âge ;
- ✓ Dédramatiser l'effort lié à l'activité physique au quotidien et mettre en évidence son caractère naturel, facile et ludique ;
- ✓ Réduire les comportements sédentaires ;
- ✓ Avoir un régime alimentaire sain et riche en fruits et légumes (au moins 5 portions par jour) ;
- ✓ Eduquer dès le jeune âge les enfants à la consommation quotidienne de fruits et légumes et à la pratique régulière de l'activité physique.
- ✓ Prendre la TA au moins une fois tous les trois mois, surtout les sujets menant une activité professionnelle indépendante ;
- ✓ Eviter le plus possible les situations de stress ;

- ✓ Consommer des aliments pauvres en graisses, sucre et sel, mais riches en fruits et légumes ;
- ✓ Pratiquer des activités physiques de 30 minutes par jour, trois fois dans la semaine ;
- ✓ Doser la glycémie au moins une fois par an ;

- ✓ Réduire de moitié, la consommation de l'alcool et du tabac.

- **A l'endroit du PNLMNT**

- ✓ Elaborer des stratégies pour la prévention de la TA élevée et de l'obésité ;
- ✓ Renforcer les compétences en matière de prévention (formation pratique des médecins et paramédicaux pour la prévention et la prise en charge de la TA élevée et l'obésité) ;
- ✓ Organiser des séances de CCC à travers des interviews, des sketches radio ou télédiffusés pour informer la population sur les complications de l'HTA et l'obésité et les facteurs associés ;
- ✓ Promouvoir de façon active, les modes de vie favorables à la santé ;
- ✓ Promouvoir la médecine scolaire, intégrer aux programmes scolaires des modules d'éducation nutritionnelle dès le niveau primaire, promouvoir l'activité physique dans les écoles en collaboration avec les autorités en charge de l'éducation.

6 Références bibliographiques

1. The World Health Report

Global Strategy on Diet, Physical activity

Geneva, World Health Organisation, 2003

2. Report of WHO meeting

Integrate Management of Cardiovascular Risk.

Geneva, World Health Organisation, 2002

3. LE PEN.C.

Prévention des maladies cardiovasculaires : une approche médico-économique. Thérapie 2001, 56, 125 – 13.

4. MS / DNPS / PNLMNT

Rapport de l'enquête STEPS dans le Littoral : Bénin 2007

5. OMS.

Stratégie de Coopération de l'OMS avec les Pays : Bénin : 2004-2008 : 11

6. ANCELLE.T.

Statistique en Epidémiologie. Edit Maloine : Paris : 2002 : 78.

7. **INSAE**

3ème Recensement Général de la Population et de l'Habitat : 2002

8. **OMS**

Manuel STEPS : partie 2 : 2-2-18

Table des matières

Sommaire ... ii
LISTE DES TABLEAUX ... iii
LISTE DES FIGURES ... vi
RESUME ... 1
1 INTRODUCTION ... 2
2 OBJECTIFS ... 5
2.1 Objectif général ... 5
2.2 Objectifs spécifiques ... 5
CADRE DE L'ETUDE ... 6
3 CADRE DE L'ETUDE: (étendue géographique) ... 7
3.1 Description et caractéristique du département de l'Atacora ... 7
3.1.1 Présentation du département ... 7
3.1.2 Les infrastructures sociocommunautaires ... 7
3.2 Description et caractéristique du département de la Donga ... 7
3.2.1 Présentation du département de la Donga ... 7
3.2.2 Infrastructures sociocommunautaires ... 8
3.3 Description et caractéristique du département de l'Atlantique ... 8
3.3.1 Présentation du département ... 8
3.3.2 Infrastructures socio- communautaires ... 8
3.4 Description et caractéristique du département du Littoral ... 9
3.4.1 Présentation du département ... 9
3.4.2 Infrastructures sociocommunautaires ... 9
3.5 Description et caractéristique du département du Mono ... 9
3.5.1 Présentation du département ... 9
3.5.2 Infrastructures sociocommunautaires ... 10
3.6 Description et caractéristique du département du Couffo ... 10
3.6.1 Présentation du département ... 10
3.6.2 Infrastructures sociocommunautaires ... 10

3.7 Description et caractéristique du département du Zou 10
3.7.1 Présentation du département 10
3.7.2 Infrastructures sociocommunautaires 11
3.8 Description et caractéristique du département des collines 11
3.8.1 Présentation du département 11
3.8.2 Infrastructures sociocommunautaires 11
3.9 Description et caractéristique du département de l'Alibori 12
3.9.1 Présentation du département 12
3.9.2 Infrastructures sociocommunautaires 12
3.10 Description et caractéristique du département du Borgou 12
3.10.1 Présentation du département 12
3.10.2 Infrastructures sociocommunautaires. 13
3.11 Description et caractéristique du département du Plateau 13
3.11.1 Présentation du département 13
3.11.2 Infrastructures sociocommunautaires 13
3.12 Description et caractéristique du département de l'Ouémé 13
3.12.1 Présentation du département 13
3.12.2 Infrastructures sociocommunautaires. 14
METHODE DE L'ETUDE 15
4 METHODE 16
4.1 Type d'étude 16
4.2 Population d'étude 16
4.3 Echantillonnage 16
4.3.1 Taille échantillon 16
La taille obtenue est de : 6853 16
4.3.2 Technique d'échantillonnage 17
4.4 Collecte des données 18
4.4.1 L'outil de collecte 18
4.4.2 Le matériel 19
4.4.3 Equipe de collecte 19
4.4.4 Durée de la collecte 19
4.4.5 Déroulement de l'enquête 19
4.4.6 Technique de collecte 20

4.4.7 Difficultés rencontrées 20
4.4.8 Considérations éthiques et consentement éclairé 20
4.5 Analyse des données et test statistiques 20
RESULTATS 22
5 RESULTATS 23
5.1 Description de la population d'étude 23
STEP 1 25
5.2 Prévalence du tabagisme 26
5.2.1 Prévalence de la consommation quotidienne de tabac 26
5.2.2 Prévalence de la consommation de tabac fumé 27
5.2.3 Prévalence de la consommation de tabac non fumé 28
5.2.4 Prévalence du tabagisme et facteurs socioculturels 30
5.3 Prévalence de la consommation d'alcool 32
5.3.1 Consommation d'alcool au cours des douze derniers mois 32
5.3.2 Consommation d'alcool au cours des trente derniers jours 34
5.4 Prévalence de l'inactivité physique 42
5.4.1 Prévalence de l'activité physique selon le niveau d'activité 42
5.4.2 Prévalence de l'inactivité physique selon le type d'activité 43
5.4.3 Nombre moyen de minutes consacrées chaque jour à une activité physique 46
5.4.4 Nombre moyen de minutes consacrées chaque jour à des comportements sédentaires 47
5.5 Prévalence de la consommation insuffisante de fruits et légumes 50
5.5.1 Nombre moyen de portions de fruits ou de légumes consommées par jour 53
5.5.2 Nombre moyen de jours de consommation de fruits ou de légumes54
STEP II 55
5.6 Prévalence de la tension artérielle élevée au Bénin en 2008 55
5.7 Prévalence de la surcharge pondérale et de l'obésité au Bénin en 2008 61
5.7.1 Prévalence de l'obésité 61
Prévalence de l'obésité en fonction des départements au Bénin en 2008 63
5.7.2 Prévalence de la surcharge pondérale 64

5.7.3 Répartition des sujets enquêtés en fonction de l'indice de masse corporelle au Bénin en 2008 66
STEP III **68**
5.8 Prévalence de l'hyperglycémie 69
Prévalence de l'hyperglycémie au Bénin selon l'ethnie **70**
Prévalence l'hyperglycémie selon le niveau d'instruction **71**
5.9 Prévalence de l'hypercholestérolémie 74
Prévalence de l'hypercholestérolémie selon l'ethnie au Bénin en 2008 .... **75**
Prévalence selon le niveau d'instruction au Bénin en 2008 .... **76**
Prévalence selon l'activité professionnelle **77**
- **A l'endroit de la population** **81**
6 Références bibliographiques **83**
Table des matières **85**
ANNEXES .... **a**
7 Annexes .... **a**
Annexe 1 La méthode Kish [8] .... a
Annexe 2 .... d
Annexe 3 .... q
Annexe 4 .... w

ANNEXES

7 Annexes

Annexe 1 La méthode Kish [8]

Introduction

La méthode Kish fournit un procédé d'échantillonnage aléatoire pour sélectionner un individu de chaque ménage.

La méthode Kish peut être utilisée pour la sélection des individus à l'intérieur des ménages quelle que soit la méthode d'échantillonnage choisie pour sélectionner ces ménages.

Echantillonnage supplémentaire pour la tranche d'âge 55-64 ans.

En fonction de la structure de la population dans votre site, il peut se révéler difficile de remplir le quota pour la tranche d'âge 55-64 ans. Pour y remédier, il est possible d'élargir l'échantillonnage pour cette tranche d'âge. Si vous décidez d'utiliser cette technique, vous incluez dans l'échantillon tous les membres des ménages sélectionnés qui ont entre 55 et 64 ans.

Si le ménage sélectionné	Alors vous sélectionnerez
A un adulte dans la tranche d'âge 55-64 ans	2 participants
N'a pas d'adulte dans la tranche d'âge 55-64 ans	1 participant

Note : Si vous décidez d'élargir l'échantillonnage, veuillez le mettre en évidence en ajustant la taille du ménage. S'il y a cinq personnes dans un ménage et que l'une d'elles entre dans la tranche 55-64 ans, la taille du ménage prise en compte pour l'échantillonnage est maintenant de quatre personnes, et l'autre membre considéré a une chance sur quatre d'être sélectionné.

Feuille de couverture Kish des ménages

• Synthèse Kish des huit tableaux	• Liste Kish des ménages
Note : Tous ces documents sont disponibles dans la Partie 6 Section 2 du présent Manuel.	
Suite à la page suivante	

Procédure	Le tableau ci-dessous établit une liste des étapes-clé pour utiliser la méthode Kish pour l'échantillonnage individuel au niveau des ménages.

Étape	Description
1	Enregistrez les informations relatives aux ménages sur la Feuille de couverture Kish des ménages.
2	Utilisez la Liste Kish des ménages pour identifier quel tableau devrait être utilisé pour ce ménage en particulier
3	Utilisez la Synthèse Kish des huit tableaux pour faire apparaître quel individu devrait être sélectionné.

Feuilles de couvertures Kish des ménages

Les Feuilles de couverture Kish des ménages rassemblent les informations sur les ménages nécessaires à la sélection aléatoire des participants issus de ces
ménages. La feuille de couverture contient des informations sur :

- le nombre d'individus au sein du ménage
- leur âge et
- leur âge

Liste Kish des ménages

La Liste Kish des ménages permet de choisir quel tableau Kish utiliser pour chaque ménage.

Le numéro du ménage est son code ou son numéro d'ordre au sein de la grappe.

Ménage	Tableau Kish
1	A
2	A
3	B1
4	B1
5	C
6	C
7	D

Ménage	Tableau Kish
8	D
9	E1
10	E2
11	F
12	F
13	A
Etc.	

Tableau	Si le nombre d'adultes dans le ménage est de :					
	1	2	3	4	5	6 et +
	Sélectionnez l'adulte portant le numéro :					
A	1	1	1	1	1	1
B1	1	1	1	1	2	2
B2	1	1	1	2	2	2
C	1	1	2	2	3	3
D	1	2	2	3	4	4
E1	1	2	3	3	3	5
E2	1	2	3	4	5	5
F	1	2	3	4	5	6

De plus amples détails pour remplir la feuille de couverture sont disponibles dans l »exemple de la partie 6 Section 2

Note : Si vous pratiquez l'échantillonnage supplémentaire des 55 – 64, ces derniers ne devraient pas figurer sur la feuille de couverture du ménage car ils ne font pas partie du processus de sélection aléatoire. Leur participation sera alors de 100%.

Annexe 2

Numéro d'identification du répondant |__|__|__||__|__|__|

Instrument STEPS de l'OMS pour la surveillance des facteurs de risque des maladies chroniques

Information sur l'enquête

**Lieu et Date**		**Réponse**			**Code**
1	Code du District	|__|__|__|			I1
2	Nom du centre/village				I2
3	Code du centre/village	|__|__|__|			I3
4	Identification de l'enquêteur	|__|__|__|			I4
5	Date de l'entretien (à laquelle l'Instrument a été rempli)	|__|__| Jour	|__|__| Mois	|__|__||__|__| Année	I5
**Consentement, Langue utilisée pour l'entretien et Nom**		**Réponse**			**Code**
6	Le formulaire de consentement a été lu au répondant	Oui<br>Non	1<br>2	**Si NON, le lire**	I6
7	Le consentement a été obtenu (oral ou écrit)	Oui<br>Non	1<br>2	**Si NON, FINIR l'entretien**	I7
8	Langue de l'entretien	Français 1<br>Anglais 2<br>Dialecte 3			I8
9	Heure de l'entretien (0-24h)	|__|__| Heure		|__|__| Minutes	I9
10	Nom de famille				I10
11	Prénom				I11
**Informations supplémentaires pouvant être utiles**					
12	Numéro de téléphone (dans la mesure du possible)				I12
13	Spécifier de quel téléphone il s'agit	Travail 1<br>Domicile 2<br>Voisin 3<br>Autres (spécifier) 4			I13

Note : Les informations I6 à I13 permettant l'identification du répondant devraient être enregistrées et archivées séparément de l'Instrument parce qu'elles contiennent des informations confidentielles.

Step 1 Informations démographiques

**MODULE DE BASE : Informations démographiques**				
14	Sexe (entourer le numéro correspondant)	Homme<br>Femme	1<br>2	C1
15	Quelle est votre date de naissance ?<br>Ne sait pas 77 777 7777	I__I__I Jour · I__I__I Mois<br>Si réponse connue, aller à C4	I__I__II__I__I Année	C2
16	Quel âge avez-vous ?	Années	I__I__I	C3
17	En tout, combien d'années avez-vous passé à l'école ou à suivre une formation à plein temps<br>(sans compter la pré-scolarité) ?	Années	I__I__I	C4
**ELARGI : Informations démographiques**		**Réponse**		**Code**
18	A quel milieu socioculturel appartenez-vous ?	Adja et apparentés<br>Fon et apparentés<br>Bariba et apparentés<br>Dendi et apparentés<br>Yom et apparentés<br>Peulh<br>Otamari<br>Yorouba<br>Africain de l'ouest<br>Autre africain<br>Non africain<br>Refuse	1<br>2<br>3<br>4<br>5<br>6<br>7<br>8<br>9<br>10<br>11<br>88	C5
19	Quel est le <u>plus haut niveau</u> d'instruction que vous ayez atteint ?	Aucune instruction officielle<br>Moins que l'école primaire<br>Fin de l'école primaire<br>Fin du secondaire niveau 1<br>Fin du secondaire niveau 2<br>École supérieure, Université<br>Diplôme post-universitaire obtenu	1<br>2<br>3<br>4<br>5<br>6<br>7	C6
		Refuse	88	
20	Laquelle des catégories suivantes décrit le mieux votre activité professionnelle <u>principale</u> ces douze derniers mois ?	Employé(e) de l'État<br>Employé(e) dans le privé<br>Indépendant(e)<br>Bénévole<br>Étudiant(e)<br>Maître (sse) de maison<br>Retraité(e)	1<br>2<br>3<br>4<br>5<br>6<br>7	C7

<table>
<tr><td></td><td></td><td colspan="3">Chômeur (se) 8</td><td></td></tr>
<tr><td></td><td></td><td colspan="3">Invalide 9</td><td></td></tr>
<tr><td></td><td></td><td colspan="3">Refuse 88</td><td></td></tr>
<tr><td>21</td><td>Combien de personnes âgées de <u>plus de dix-huit ans</u>, y compris vous-même, habitent dans votre maison ?</td><td colspan="3">Nombre de personnes I__I__I</td><td>C8</td></tr>
<tr><td rowspan="4">22</td><td rowspan="4">En prenant comme <u>référence l'année passée</u>, pouvez-vous me dire quels ont été les revenus moyens du ménage ?
(NOTER SEULEMENT POUR UNE PERIODE ET PAS LES TROIS)</td><td>Par semaine</td><td>I__I__II__I__I</td><td>Aller à T1</td><td>C9a</td></tr>
<tr><td>Ou par mois</td><td>I__I__II__I__I</td><td>Aller à T1</td><td>C9b</td></tr>
<tr><td>Ou par année</td><td>I__I__II__I__I</td><td>Aller à T1</td><td>C9c</td></tr>
<tr><td>Refuse</td><td>8</td><td></td><td>C9d</td></tr>
<tr><td>23</td><td>Si vous ne connaissez pas le montant, pouvez-vous me donner une estimation du revenu annuel du ménage si je vous lis quelques montants ? Est-ce…</td><td colspan="3">≤ 66 000F 1
[67 000 - 132 000] 2
[133 000 - 198 000] 3
[199 000 - 264 000] 4
> 264 000 5
Ne sait pas 7
Refuse 8</td><td>C10</td></tr>
</table>

Step 1 Mesures comportementales

MODULE DE BASE : Consommation de tabac					
Question		Réponse			Code
24	Fumez-vous actuellement des produits à base de tabac tels que cigarettes, cigares ou pipes ?	Oui	1		T1
		Non	2	*Si Non, aller à T6*	
25	<u>Si Oui,</u> Fumez-vous quotidiennement ?	Oui	1		T2
		Non	2	*Si Non, aller à T6*	
26	A quel âge avez-vous commencé à fumer quotidiennement ?	Age (années)	I__I__II__I	*Si réponse connue aller à T5a*	T3
		Ne se souvient pas		777	
27	Si vous ne vous rappelez pas de votre âge, vous souvenez-vous depuis quand vous avez commencé à fumer quotidiennement ?<br>*(NOTER SEULEMENT POUR UNE PERIODE ET PAS LES TROIS)*<br><br>Ne se souvient pas 777	En nombre d'années	I__I__II__I	*Si réponse connue, aller à T5a*	T4a
		Ou en Mois	I__I__II__I	*Si réponse connue, aller à T5a*	T4b
		Ou en Semaines	I__I__II__I		T4c
28<br><br>▪	Quelle quantité des produits suivants fumez-vous en moyenne chaque jour ?<br><br>*(INSCRIRE LA QUANTITE POUR CHAQUE TYPE DE PRODUIT)*<br><br>Ne se souvient pas 777	Cigarettes industrielles		I__I__II__I	T5a
		Cigarettes roulées		I__I__II__I	T5b
		Pipes		I__I__II__I	T5c
		Cigares, cigarillos		I__I__II__I	T5d
		Autres	I__I__II__I	*Si Autres, aller à T5 other*	T5e
		Autres (à spécifier)	I__I__II__II__I__II__I		T5other

ELARGI : Consommation de tabac					
**Question**		**Réponse**			**Code**
29	Dans le passé, avez-vous déjà fumé quotidiennement ?	Oui 1			T6
		Non 2		*Si Non, aller à T6*	
30	<u>Si Oui,</u> Quel âge aviez-vous quand vous avez arrêté de fumer quotidiennement ?	Age (années)	I__I__II__I	*Si réponse connue aller à T9*	T7
		Ne se souvient pas		777	
31	Si vous ne vous rappelez pas de votre âge, souvenez-vous depuis quand vous avez arrêté de fumer quotidiennement ? *(NOTER SEULEMENT POUR UNE PERIODE ET PAS LES TROIS)* Ne se souvient pas 777	En nombre d'années	I__I__II__I	*Si réponse connue, aller à T9*	T8a
		Ou Nombre de Mois	I__I__II__I	*Si réponse connue, aller à T9*	T8b
		Ou Nombre de Semaines	I__I__II__I		T8c
32	Consommez-vous actuellement du tabac non fumé sous forme de tabac à priser, de tabac à mâcher, de feuilles de bétel ?	Oui 1			T9
		Non 2		*Si Non, aller à T12*	
33	<u>Si Oui,</u> En consommez-vous quotidiennement ?	Oui 1			T10
		Non 2		*Si Non, aller à T12*	
**ELARGI : Consommation de tabac, suite**					
34	En moyenne, combien de fois par jour consommez-vous les produits suivants ? *(INSCRIRE POUR CHAQUE TYPE)* Ne sait pas 777	Tabac à priser (voie orale)		I__I__II__I	T11a
		Tabac à chiquer (voie orale)		I__I__II__I	T11b
		Tabac à mâcher		I__I__II__I	T11c
		Feuilles de bétel		I__I__II__I	T11d
		Autres	I__I__II__I	*Si Autres, Aller à T11 other*	T11e
		Autres (Spécifier)	I__I__II__II__I__II__I		T11other
35	Dans le passé, avez-vous déjà consommé quotidiennement du tabac non fumé tel que [tabac à priser, tabac à mâcher, feuilles de bétel] ?	Oui 1			T12

		Non	2	

MODULE DE BASE : Consommation d'alcool

Question		Réponse			Code
36	Avez-vous consommé une boisson alcoolisée comme de la bière, du vin, de la liqueur, du cidre ou des boissons alcoolisées locales ces <u>douze derniers mois ?</u><br>*UTILISER LES CARTES OU MONTRER DES EXEMPLES*	Oui			A1
		Non	2	*Si Non, aller à D1*	
37<br>▪	Durant les douze derniers mois, à quelle fréquence avez-vous bu au moins une boisson alcoolisée ?	Quotidiennement<br>5-6 jours par semaine<br>1-4 jours par semaine<br>1-3 jours par mois<br>Moins d'1 fois par mois		1<br>2<br>3<br>4<br>5	A2
38	Quand vous buvez de l'alcool, combien de verres prenez-vous en moyenne par jour ?	Nombre<br>Ne sait pas 77		I__I__I	A3
39	Avez-vous consommé une boisson alcoolisée comme de la bière, du vin, de la liqueur, du cidre, <u>ces trente derniers jours</u> ?	Oui	1		A4
		Non	2	*Si Non, aller à A6*	
40	Au cours des 7 derniers jours, combien de verres standards d'alcool avez-vous pris (quelle que soit la boisson alcoolisée) ?	Lundi		I__I__I	A5a
		Mardi		I__I__I	A5b
		Mercredi		I__I__I	A5c
		Jeudi		I__I__I	A5d
		Vendredi		I__I__I	A5e
		Samedi		I__I__I	A5f
		Dimanche		I__I__I	A5g

ELARGI : Consommation d'alcool

Question		Réponse		Code
41	Au cours des 12 derniers mois, quel a été le plus grand nombre de verres que vous ayez bu en une seule fois, en comptant tous les verres standard ?	Le plus grand nombre de verres	I__I__I	A6
42	<u>Pour les hommes uniquement :</u><br>Combien de jours avez-vous bu cinq verres standard ou plus en un seul jour	Nombre de jours	I__I__II__I	A7

	au cours des 12 derniers mois ?			
43	<u>Pour les femmes seulement</u> *:* Combien de jours avez-vous bu quatre verres standard ou plus en un seul jour au cours des 12 derniers mois ?	Nombre de jours	I__I__II__I	A8

**MODULE DE BASE : Hygiène alimentaire**					
44	Habituellement, combien de jours par semaine consommez-vous des fruits ?	Nombre de jours<br>Ne sait pas 77	I__I__I	Si aucun jour, aller à D3	D1
45	Combien de portions de fruits mangez-vous lors d'une de ces journées ?	Nombre de portions<br>Ne sait pas 77	I__I__I		D2
46	Habituellement, combien de jours par semaine consommez-vous des légumes ?	Nombre de jours<br>Ne sait pas 77	I__I__I	Si aucun jour, aller à D5	D3
47	Combien de portions de légumes mangez-vous lors d'une de ces journées ?	Nombre de portions<br>Ne sait pas 77	I__I__I		D4
**ELARGI : Hygiène alimentaire**					
48	Quelle sorte de matière grasse (huile, beurre…) utilisez-vous le plus souvent pour la préparation des repas à la maison ?	Huile végétale<br>Lard ou graisse<br>Beurre ou beurre allégé<br>Margarine<br>Autres 5<br>Aucune en particulier		1<br>2<br>3<br>4<br>*Si Autres, aller à D5othe other*<br>6	D5
		Aucune utilisée<br>Ne sait pas		7<br>77	
		Autres	I__I__II__I__I		D5 other

MODULE DE BASE : Activité physique					
**Question**		**Réponses**			**Code**
**Activités au travail**					
49	Est-ce que votre travail implique des activités physiques de forte intensité qui nécessitent une augmentation conséquente de la respiration ou du rythme cardiaque, comme [soulever des charges lourdes, travailler sur un chantier, effectuer du travail de maçonnerie] pendant au moins 10 minutes d'affilée ?	Oui	1		P1
		Non	2	*Si Non, aller à P4*	
50	Habituellement, combien de jours par semaine effectuez-vous des activités physiques de forte intensité dans le cadre de votre travail ?	Nombre de jours		I__I	P2
51	Lors d'une journée habituelle durant laquelle vous effectuez des activités physiques de forte intensité, combien de temps consacrez-vous à ces activités ?	Heure : Minutes	I__I__I Heure	I__I__I Minutes	P3 (a-b)
52	Est-ce que votre travail implique des activités physiques d'intensité modérée, comme une marche rapide ou [soulever une charge légère] durant au moins 10 minutes d'affilée ?	Oui	1		P4
		Non	2	*Si Non, aller à P7*	
53	Habituellement, combien de jours par semaine effectuez-vous des activités physiques d'intensité modérée dans le cadre de votre travail ?	Nombre de jours		I__I	P5
54	Lors d'une journée habituelle durant laquelle vous effectuez des activités physiques d'intensité modérée, combien de temps consacrez-vous à ces activités ?	Heure : Minutes	I__I__I Heure	I__I__I Minutes	P6 (a-b)
**Se déplacer d'un endroit à l'autre**					
55	Est-ce que vous effectuez des trajets d'au moins 10 minutes à pied ou à vélo ?	Oui	1		P7
		Non	2	*Si Non, aller à P 10*	
56	Habituellement, combien de jours par semaine effectuez-vous des trajets d'au moins 10 minutes à pied ou à vélo ?	Nombre de jours		I__I	P8
57	Lors d'une journée habituelle, combien de temps consacrez-vous à vos déplacements à pied ou à vélo ?	Heure : Minutes	I__I__I Heure	I__I__I Minutes	P9 (a-b)

**Activités de loisirs**					
58	Est-ce que vous pratiquez des sports, du fitness ou des activités de loisirs de forte intensité qui nécessitent une augmentation importante de la respiration ou du rythme cardiaque comme [courir ou jouer au football] pendant au moins dix minutes d'affilée ?	Oui	1		P10
		Non	2	*Si Non, aller à P 13*	
59	Habituellement, combien de jours par semaine pratiquez-vous une activité sportive, du fitness ou d'autres activités de loisirs de forte intensité ?	Nombre de jours		I__I	P11
60	Lors d'une journée habituelle, combien de temps y consacrez-vous ?	Heure : Minutes	I__I__I Heure	I__I__I Minutes	P12 (a-b)
61	Est-ce que vous pratiquez des sports, du fitness ou des activités de loisirs d'intensité modérée qui nécessitent une petite augmentation de la respiration ou du rythme cardiaque comme la marche rapide [faire du vélo, nager, jouer au volley] pendant au moins dix minutes d'affilée ?	Oui	1		P13
		Non	2	*Si Non, aller à P 16*	
62	Habituellement, combien de jours par semaine pratiquez-vous une activité sportive, du fitness ou d'autres activités de loisirs d'intensité modérée ?	Nombre de jours		I__I	P14
63	Lors d'une journée habituelle, combien de temps y consacrez-vous ?	Heure : Minutes	I__I__I Heure	I__I__I Minutes	P15 (a-b)
**Comportement sédentaire**					
64	Combien de temps passez-vous en position assise ou couchée lors d'une journée habituelle ?	Heure : Minutes	I__I__I Heure	I__I__I Minutes	P16 (a-b)

ELARGI : Antécédents de tension artérielle élevée				
**Questions**		**Réponses**		**Code**
65	Quand est-ce que votre tension artérielle a été prise pour la dernière fois par un professionnel de santé ?	Au cours des 12 derniers mois	1	H1
		Entre 1-5 années	2	
		Pas ces 5 dernières années	3	
66	Au cours des 12 derniers mois, est-ce qu'un professionnel de santé vous a dit que vous aviez une tension artérielle élevée ou que vous souffriez d'hypertension ?	Oui	1	H2
		Non	2	
67	Recevez-vous actuellement les traitements suivants prescrits par un professionnel de santé pour une tension artérielle élevée, et/ou les conseils suivants ?			
	Médicaments pris ces deux dernières semaines	Oui	1	H3a
		Non	2	
	Régime spécial prescrit	Oui	1	H3b
		Non	2	
	Conseil ou traitement pour perdre du poids	Oui	1	H3c
		Non	2	
	Conseil ou traitement pour arrêter de fumer	Oui	1	H3d
		Non	2	
	Conseil pour commencer une activité physique ou en augmenter la fréquence	Oui	1	H3e
		Non	2	
68	Au cours des 12 derniers mois avez-vous vu un guérisseur traditionnel pour une tension artérielle élevée ou pour de l'hypertension ?	Oui	1	H4
		Non	2	
69	Prenez-vous actuellement un remède traditionnel ou à base d'herbes pour votre tension artérielle élevée ?	Oui	1	H5
		Non	2	

ELARGI : Antécédents de diabète				
Questions		Réponses		Code
70	A-t-on mesuré votre glycémie ces 12 derniers mois ?	Oui	1	H6
		Non	2	
71	Est-ce qu'un professionnel de santé vous a déjà dit que vous aviez du diabète au cours de ces 12 derniers mois ?	Oui	1	H7
		Non	2	
72	Recevez-vous actuellement les traitements suivants prescrits par un professionnel de santé pour le diabète, et/ou les conseils suivants ?			
	Insuline	Oui	1	H8a
		Non	2	
	Médicaments par voie orale que vous avez pris ces 2 dernières semaines	Oui	1	H8b
		Non	2	
	Régime spécial prescrit	Oui	1	H8c
		Non	2	
	Conseil ou traitement pour perdre du poids	Oui	1	H8d
		Non	2	
	Conseil ou traitement pour arrêter de fumer	Oui	1	H8e
		Non	2	
	Conseil pour commencer une activité physique ou en augmenter la fréquence	Oui	1	H8f
		Non	2	
73	Au cours des 12 derniers mois avez-vous vu un guérisseur traditionnel pour le diabète ?	Oui	1	H9
		Non	2	
74	Prenez-vous actuellement un remède traditionnel ou à base d'herbes pour votre diabète ?	Oui	1	H10
		Non	2	

Step 2 Mesures physiques

<table>
<tr><td colspan="6">MODULE DE BASE : Taille et poids</td></tr>
<tr><td colspan="2">Questions</td><td colspan="3">Réponses</td><td>Code</td></tr>
<tr><td>75</td><td>Code ID de l'enquêteur</td><td colspan="3">|__|__|__|__|</td><td>M1</td></tr>
<tr><td rowspan="2">76</td><td rowspan="2">Code de la toise et du pèse-personne</td><td colspan="2">Taille</td><td>|__|__|</td><td>M2a</td></tr>
<tr><td colspan="2">Poids</td><td>|__|__|</td><td>M2b</td></tr>
<tr><td>77</td><td>Taille</td><td>en Centimètres (cm)</td><td colspan="2">|__|__|__| |__|</td><td>M3</td></tr>
<tr><td>78</td><td>Poids
Si trop lourd pour le pèse-personne, codez 666.6</td><td>en Kilogrammes (kg)</td><td colspan="2">|__|__|__| |__|</td><td>M4</td></tr>
<tr><td rowspan="2">79</td><td rowspan="2">(Pour les femmes) Êtes-vous enceinte ?</td><td>Oui</td><td>1</td><td>Si Oui, aller à M 8</td><td rowspan="2">M5</td></tr>
<tr><td>Non</td><td>2</td><td></td></tr>
<tr><td colspan="6">BASE : Tour de Taille</td></tr>
<tr><td>80</td><td>Code ID pour la taille</td><td colspan="3">|__|__|</td><td>M6</td></tr>
<tr><td>81</td><td>Tour de taille</td><td>en Centimètres (cm)</td><td colspan="2">|__|__|__| |__|</td><td>M7</td></tr>
<tr><td colspan="6">BASE : Tension artérielle</td></tr>
<tr><td>82</td><td>Code ID de l'enquêteur</td><td colspan="3">|__|__|</td><td>M8</td></tr>
<tr><td>83</td><td>Code ID pour la tension artérielle</td><td colspan="3">|__|__|</td><td>M9</td></tr>
<tr><td>84</td><td>Largeur du brassard</td><td colspan="2">Petit
Moyen
Large</td><td>1
2
3</td><td>M10</td></tr>
<tr><td rowspan="2">85</td><td rowspan="2">Mesure 1</td><td colspan="2">Systolique (mmHg)</td><td>|__|__|__|</td><td>M11a</td></tr>
<tr><td colspan="2">Diastolique (mmHg)</td><td>|__|__|__|</td><td>M11b</td></tr>
<tr><td rowspan="2">86</td><td rowspan="2">Mesure 2</td><td colspan="2">Systolique (mmHg)</td><td>|__|__|__|</td><td>M12a</td></tr>
<tr><td colspan="2">Diastolique (mmHg)</td><td>|__|__|__|</td><td>M12b</td></tr>
<tr><td rowspan="2">87</td><td rowspan="2">Mesure 3</td><td colspan="2">Systolique (mmHg)</td><td>|__|__|__|</td><td>M13a</td></tr>
<tr><td colspan="2">Diastolique (mmHg)</td><td>|__|__|__|</td><td>M13b</td></tr>
<tr><td rowspan="2">88</td><td rowspan="2">Au cours des deux dernières semaines, avez-vous suivi un traitement, prescrit par un professionnel de santé, pour une tension artérielle élevée ?</td><td colspan="2">Oui</td><td>1</td><td rowspan="2">M14</td></tr>
<tr><td colspan="2">Non</td><td>2</td></tr>
<tr><td colspan="6">ELARGI : Tour de hanches et Rythme cardiaque</td></tr>
<tr><td>89</td><td>Tour de hanches</td><td>en Centimètres (cm)</td><td colspan="2">|__|__|__| |__|</td><td>M15</td></tr>
<tr><td rowspan="2">90</td><td colspan="5">Rythme cardiaque (Indiquez si un appareil automatique de tension artérielle est utilisé)</td></tr>
<tr><td>Mesure 1</td><td colspan="2">Battements par minute</td><td>|__|__|__|</td><td>M16a</td></tr>
</table>

	Mesure 2	Battements par minute	I__I__I__I	M16b
	Mesure 3	Battements par minute	I__I__I__I	M16c

Step 3 Mesures biochimiques

**BASE : Glycémie**					
**Questions**		**Réponses**			**Code**
91	Au cours des 12 dernières heures, avez-vous bu ou mangé quelque chose, autre que de l'eau ?	Oui	1		B1
		Non	2		
92	Code ID du technicien	I__I__I__I			B2
93	Code ID de l'appareil	I__I__I__I			B3
94	Heure de la prise de sang (0-24 heures)	Heure : Minutes	I__I__I Heure	I__I__I Minutes	B4
95	Glycémie à jeun	mmol/l	I__I__I	I__I__I	B5
**BASE : Lipides sanguins**					
96	Code ID de l'appareil	I__I__I__I			B6
97	Cholestérol total	mmol/l	I__I__I	I__I__I	B7
**ELARGI : Triglycérides et Cholestérol HDL**					
98	Triglycérides	mmol/l	I__I__I	I__I__I	B8
99	Cholestérol HDL	mmol/l	I__I__I	I__I__I	B9

Annexe 3

Evaluation de la consommation d'alcool

La consommation d'alcool a porté sur la fréquence de consommation et la quantité consommée.

La quantification de la consommation d'alcool s'est faite en nombre de verres standards. Les verres standards diffèrent d'une boisson à une autre en fonction de leur titre alcoolique. Le titre alcoolique des bières varient entre 4,5° et 7,5° (Annexe VII). Nous avons considéré dans cette étude 5° pour toutes les bières. Les boissons alcoolisées locales telles que le TCHOUKOUTOU (shukutu), le ATAN et le SODABI titrent respectivement 8°,5° et 45° [54]. Nous avons considéré dans cette étude un titre alcoolique de 5° pour le TCHOUKOUTOU.

Les volumes des verres standards des boissons figurent à l'annexe V. [55]. Les volumes des verres standards des boissons variant en fonction de leur degré alcoolique, les enquêteurs étaient munis d'une table de conversion (Voir Annexe V). Le nombre de verre standard d'alcool consommé par un sujet a été obtenu en divisant le volume de la boisson consommée par le volume de son verre standard.

Pour les bières, le volume consommé a été obtenu en multipliant le nombre de bouteille consommé (½ bouteille, 1 bouteille, 2 bouteilles, etc.) par le volume de la bouteille (Annexe VII). Le nombre de verres standards a été obtenu en divisant le volume ainsi calculé (en ml) par 250.

Pour le SODABI, le volume consommé a été déterminé à partir du volume du TALOKPEMI. Les TALOKPEMI ont une capacité de 40 à 45ml. Nous avons considéré la moyenne de 42,5ml.

$$1\ \textbf{TALOPKPEMI de}\ SODABI = 42,\frac{5}{25} = 1,7\ \text{verre standard}$$

Le nombre de verres standards consommé est obtenu en multipliant le nombre de TALOKPEMI consommé par 1,7.

Pour les boissons autres que les bières ou celles qui ne sont pas consommées par le TALOKPEMI (TCHOUKOUTOU, ATAN etc.), le volume consommé par le sujet a été déterminé à partir du bol gradué. Il a été demandé pour ce faire au sujet d'estimer le volume consommé à partir du gradué (1 bol gradué, 2 bols gradués, etc.).

Lorsque le sujet a un bol ou un verre fixe qu'il utilise habituellement, le volume de ce bol ou verre est déterminé sur place par l'enquêteur à l'aide du bol gradué. Le nombre de verres standards est obtenu en divisant le volume consommé ainsi calculé (en ml) par 25 pour les boissons titrant 45° et par 30 pour celles titrant 40°.

Annexe 4

GRAPPES SELECTIONNEES/STRATIFICATION

N°	DEPARTEMENTS	ARRONDISSEMENTS	GRAPPES	POP. ARR	QUARTIERS/ VILLAGES	POP. VILLAGE	NOMBRE PERS
1	PLATEAU	IKPINLE	1	**16 372**	ATAN-EWE	1 288	12
					IGBO-IROKO	641	7
					IKPINLE	7 348	68
					ITABOLARINWA	3325	30
		LAGBE	2	**9 138**	HOUMBODJEDJE	1285	35
					KOUYE	664	18
					LAGBE	863	23
					SOKOU	1 472	40
		KETOU	3	**25102**	ATCHOUBI	4642	38
					IDJABO	1299	11
					MASSAFE	6957	58
					ODIARO	705	6
					OGUIDIGBO	185	2
		AGUIDI	4	**12 739**	AGADA-HOUNME	1015	22
					ASSAIDIOTCHE	1967	24
					ILAKOIDIORO	1102	25
					KOBEDJO	955	22
					MODOGAN	994	22
2	ATLANTIQUE	GODOMEY	5 / 6	**153 447**	COCOCODJI	14856	31
					GODOMEY-N'GBEHO	1223	3
					SALAMEY	23219	50
					GODOMEY TOGOUDO	21468	93

w

N°	DEPARTEMENTS	ARRONDISSEMENTS	GRAPPES	POP. ARR	QUARTIERS/ VILLAGES	POP. VILLAGE	NOMBRE PERS
					YLOMAHOUTO	24396	53
		ABOMEY- CALAVI	7	**61450**	AGORI	28534	73
					KANSOUKPA	2412	7
					SEME	13695	35
		SEKOU	8	**16 124**	HOUINDJANAWA	1431	16
					DODJI ALIHO	2390	28
					HOUNDADJA	758	8
					SEKOU CENTRE	4392	50
					SOHON	561	7
					VEHOUI	432	5
		GAKPE	9	4776	AMOULEHOUE	680	40
					FONKOUNME	1286	75
		HOUEDO-AGUEKON	10	10610	GANVIECOMEY	1704	34
					GBEGODO	2322	47
					GBESSOU	1611	33
		SEHOUE	11	12081	ACLONME	2512	52
					AKLISSA	1026	22
					SOME	1969	41
		TANGBODJEVIE	12	9604	ANAVIE	537	12
					GLEGBODJI II	1086	25
					HOUEZE	861	19
					TANGBO AGA	1842	42
					YEVI	680	15
3	ALIBORI	OUNET	13	11897	OUNET A	1832	30
					OUNETB	2317	37
					SENNOU PEULH	2934	48
		SORI	14	21735	GAMAGOU	1761	21
					OUESSENE PEULH	1229	16
					PIGOUROU	2402	30

N°	DEPARTEMENTS	ARRONDISSEMENTS	GRAPPES	POP. ARR	QUARTIERS/ VILLAGES	POP. VILLAGE	NOMBRE PERS
					SORI PEULH	3969	49
		KANDI 1	15	8172	DAMADI	1401	39
					GANDO KOSSIKANA	1283	35
					GANSOSSO	1497	41
		MADECALI	16	14105	KASSA	3507	42
					MADECALI	6202	74
4	ZOU	AGBOKPA	17	5042	DOKON	1457	45
					GNANSATA	1147	36
					SONOUAKOUTA	1091	34
		TANVE	18	8034	DEKANME	1324	28
					HOUALA	1229	27
					KPODJI-AGA	1161	25
					TANVE	1684	36
		BOHICON 2	19	37546	AGONVEZOUN	3733	25
					GANKONPONSA	3018	20
					GBANHICON	4683	31
					KPOKON	5507	38
		DJIDJA	20	15549	AGONHOHOUN	692	13
					DONA	803	15
					KOME	960	18
					SOVLEGNI	1510	29
					WOGBAYE	869	16
					ZINKANME	1229	23
		KPAKPAME	21	10254	AFFOSSOWOGBA	907	19
					DRAME	1985	42
					GUINGNIN-MLINKPIN	730	16
					SOME	1805	38
		ZOUKOU	22	6108	AGRIMEY	842	36

N°	DEPARTEMENTS	ARRONDISSEMENTS	GRAPPES	POP. ARR	QUARTIERS/ VILLAGES	POP. VILLAGE	NOMBRE PERS
					BOGNONGON	969	40
					KOTOAYIVEDJI	915	38
5	LITTORAL	3ème ARRONDISSEMENT	23	59830	ADOGLETA	5500	19
					GBENONKPO	3171	10
					KPANKPAN	5637	20
					SEGBEYA SUD	2133	7
					AGBATO	6143	21
					AGBODJEDO	4930	16
					AYELAWADJE I	7181	24
		5ème ARRONDISSEMENT	24	32864	TOKPA HOHO	1899	14
					GBETO	914	7
					MIFONGOU	2001	15
					JONQUET	2167	16
					BOCOSSI TOKPA	1799	13
					GBEDOKPO	2606	19
					MISSEBO	1539	11
					MISSITE	2483	19
		8ème ARRONDISSEMENT	25	37631	AGONTIKON	4558	25
					HOUEHOUN	5017	27
					HOUENOUSSOU	6095	34
					MINONKPO	5160	29
		10ème ARRONDISSEMENT	26	41806	KOUHOUNOU	11670	48
					MISSEPLE	4336	18
					MISSOGBE	3129	13
					YENAWA	8911	37
		13ème ARRONDISSEMENT	27	63572	AGLA	37880	86
					AIBATIN II	10128	23
					HOUENOUSSOU	3209	7
6	MONO	LOBOGO	28	17622	ADJAME	415	6

z

N°	DEPARTEMENTS	ARRONDISSEMENTS	GRAPPES	POP. ARR	QUARTIERS/ VILLAGES	POP. VILLAGE	NOMBRE PERS
					DJOFOUN	898	14
					LABOGO GBETOCOME	4059	60
					HANGNANME	924	14
					ATOHOUE	444	7
					YONOUHOUE	914	14
		AVLOH	29	3416	AVLOH PLAGE	590	27
					HOHOUE	1129	50
					KOENTA	861	38
		KOUDO	30	12427	KOUDO	2761	42
					TINOU	1425	22
					TOZOUME	3385	51
7	OUEME	AKPADANOU	31	6158	ALLANDOHOU I	395	16
					ALLANDOHOU II	630	27
					HOUEDO-AGUE	332	14
					KPATINSA	694	29
					SOKPETINKON	677	29
		VAKON	32	20541	GOUAKO, KOTOCLOME	2290	23
					VAAKON, AZOHOUE	4719	48
					VAKON, GBO	4299	44
		AVRANKOU	33	13734	AVALIGBO	950	16
					DANGBODJI	939	16
					HOUEZE	2314	40
					KOGBOME	802	14
					LATCHE OUEZOUNME	1659	29
		1er ARRONDISSEMENT PORTO-NOVO	34	33830	ACCRON GOGANKOME	3855	18
					ADJEGOUNLE	2205	10
					AHOUANTINKOMEY	2381	11
					AVASSA BAGORO AGBOKOME	1239	5

N°	DEPARTEMENTS	ARRONDISSEMENTS	GRAPPES	POP. ARR	QUARTIERS/ VILLAGES	POP. VILLAGE	NOMBRE PERS
					FLEKOMEY-ILEFIE	946	4
					GBEKON	3170	15
					HONDJI HONNOU FILLA	1002	4
					HOUEGBO HLINKOMEY	1400	7
					HOUEYOGBE GBEDJI	1183	5
					KPOTA SANDODO	951	4
					OGANLA GARE EST	3643	17
					SADOGNON ADJEGOUNLE	1362	7
					SADOGNON WOUSSA	1363	7
		4ème ARRONDISSEMENT PORTO-NOVO	35	57311	ANAVIE	6449	21
					DJEGAN KPEVI	5234	18
					DODJI	5097	17
					GBEDJROMEDE	6612	21
					GUEVIE	3653	12
					HLOMGOU	3108	10
					KANDEVIE-MISSOGBE	4759	16
		AGBLANGANDAN	36	30716	AGBALILAME	5242	32
					AGLANGANDAN	6164	37
					DAVATIN	613	3
					LOKOKOUCOUME	5921	36
					MOUDOKOME	1055	7
8	COUFFO	AZOVE	37	22853	AVEGODOUI	3084	22
					AZOVE	5031	35
					DJIMADOHOUE	2094	15
					EKINHOUE	3173	23
					YEHOUEMEY	2693	20
		ADJINTINMEY	38	10691	AGOHOUE-BALLIMEY	2140	44
					GBOTOHOUE	1331	26
					HEKPE	2349	44

N°	DEPARTEMENTS	ARRONDISSEMENTS	GRAPPES	POP. ARR	QUARTIERS/ VILLAGES	POP. VILLAGE	NOMBRE PERS
		HONTON	39	5814	ATCHANHOUE	1262	47
					KOUTIME	871	32
					KPOHA	979	36
		HONDJI	40	6873	HONDJIN AKPAHOUE	2342	63
					KOGBETOHOUE	1324	35
					KOME	597	16
		LALO	41	10038	LALO CENTRE	4286	66
					GOULOKO	1442	22
					ZONMONDJI	1768	27
9	COLLINES	GOUKA	42	13765	GALATA	2083	26
					GOUKA	3448	44
					MAMATCHOKPE	1179	15
					MAYAMON	2420	30
			43		ESSEBRE	928	23
		DASSA1			ESSEKPA	1784	43
					LATIN	1973	48
		GLAZOUE	44	12822	AFFECIA	3550	47
					OROKOTO	1140	14
					ZONGO	4119	54
		DOUME	45	13592	ABALA	1258	18
					AFE-ZOUNGO	2596	37
					DOUME-LAKOUN	1882	26
					KANAOUN	1565	22
					KOFFE AGBALA	858	12
		OFE	46	9926	AYEDJOKO	2153	34
					DANI	2689	42
					GOBE	2420	38
10	BORGOU	INA	47	16558	GUESSOU-SUD	4613	45

N°	DEPARTEMENTS	ARRONDISSEMENTS	GRAPPES	POP. ARR	QUARTIERS/ VILLAGES	POP. VILLAGE	NOMBRE PERS
					INA I	3188	31
					INA II	1973	20
					INA PEULH	1231	13
					KONOU	618	6
		N'DALI	48	15314	SAKAROU	928	8
					SUANIN	4943	42
					WOBA KAROU	7767	65
		GNONKOURAKALI	49	9130	GBARI	2054	34
					GNONKOURAKALI	2304	38
					GUINROU PEULH	1082	18
					SOUBO	1530	25
		1er ARRONDISSEMENT PARAKOU	50	66994	ALAGA	5042	12
					ALBARIKA	6817	16
					TOUROU I	2081	5
					TOUROU IV	4076	10
					TOUROU V	2668	7
					BAPEROU	2441	6
					KADERA	1010	3
					MADINA	3612	9
					SAWARAROU	189	0
					SINAGOUROU	5290	13
					TITIROU	9066	23
					ZAZIRA	3437	9
		3ème ARRONDISSEMENT PARAKOU	51	37060	AMAOUIGNON DEKPAROU	3589	28
					GAH CENTRE	3878	31
					TRANZA	3861	31
					SWINROU	3076	25
		SINENDE	52	25984	DIADIA	2147	15

dd

N°	DEPARTEMENTS	ARRONDISSEMENTS	GRAPPES	POP. ARR	QUARTIERS/ VILLAGES	POP. VILLAGE	NOMBRE PERS
					GNANRO-BARIBA	4331	28
					GOUROU	3717	24
					GUESSOU BANI	3939	25
					LEMANOU	3381	22
11	ATACORA	MANTA	53	10683	DIKON HEIN	1385	23
					DIPOKOR I	1223	20
					KOUHINGOU	592	9
					KOUKOUANGOU	1580	26
					KOUTANGOU	1161	18
					TATCHADIETA	1083	18
		COBLY	54	17809	COBLY	4646	43
					KOUKONTOUGA	946	9
					NANAGADE	4036	37
					OUOROU	1642	16
					TOUGA	1220	11
		OROUKAYO	55	15832	BOROUKOU-PEULH	237	4
					DEKEROU	1427	20
					GANIKPEROU	1800	26
					NIARO-GNINON	1302	19
					NIEKENE BANSOU	3171	46
		MATERI	56	17887	KANKINI-SER	1898	19
					MATERI	4255	44
					NAGASSEGA-KANI	1523	16
					SOMOU	1840	19
					YONDISSERI	1570	16
		PEHUNCO	57	27101	PEHUNCO I	4045	30
					PEHUNCO II	3194	24
					SINAOURAROU	3462	26
					SOAODOU	2790	21

ee

N°	DEPARTEMENTS	ARRONDISSEMENTS	GRAPPES	POP. ARR	QUARTIERS/ VILLAGES	POP. VILLAGE	NOMBRE PERS
					SOASSARAROU	956	7
					SOMPAREROU-GAH	964	7
12	DONGA	ALEDJO	58	11988	ALEDJO	4499	58
					BOUTOU	971	12
					PARTAGO	2763	37
					TCHIMBERI	583	8
		COPARGO	59	19020	GALORA	3537	38
					PASSABIA	3246	35
					SATIEKA	3901	42
		SEROU	60	10382	ALFA-KPARA	2866	49
					BOUNVARI	1854	32
					SEROU	2026	34

ff
